# Hypoxia Reprograms a Ciliary Scaffold into a Metabolic-Epigenetic Trafficking Hub via a Succinate-Driven DNMT3L-ASXL2 Axis

**DOI:** 10.7150/ijbs.137355

**Published:** 2026-10-09

**Authors:** Jun Yin, Zhifeng Zhong, Mengjie Zhang, Debao Li, Aiping Wang, Qianying Huang, Qinghua Zhang, Bing Ni, Wei He

**Affiliations:** 1 Department of Pathophysiology, Army Medical University, Chongqing 400038, China.; 2 Key Laboratory of Extreme Environmental Medicine, Ministry of Education of China, Chongqing 400038, China.; 3 Key Laboratory of High Altitude Medicine, PLA, Chongqing 400038, China.; 4 Department of High Altitude Operational Medicine, College of High Altitude Military Medicine, Army Medical University, Chongqing 400038, China.; 5 Department of Immunology, School of Basic Medical Sciences, Southern Medical University, Guangzhou 510515, China.; 6 Department of Frigid Zone Medicine, College of High Altitude Military Medicine, Army Medical University, Chongqing 400038, China.; 7 The Clinical Research Center, Southwest Hospital, Army Medical University, Chongqing 400038, China.; 8 Department of Obstetrics and Gynecology, Daping Hospital, Army Medical University, Chongqing 400042, China.; 9 Reproductive Medical Center, Southwest Hospital, Army Medical University, Chongqing 400038, China.

**Keywords:** environmental hypoxia, asthenozoospermia, CEP162, DNMT3L, Rab1a

## Abstract

Environmental hypoxia is a major yet mechanistically unresolved driver of male infertility. While the canonical hypoxia-inducible factor (HIF) and reactive oxygen species (ROS) pathways dominate current models, how chronic oxygen deprivation persistently rewires spermatogenic epigenetics and flagellar architecture remains unknown. Here, we identify CEP162—traditionally defined as a static ciliary transition zone scaffold—as a dynamic oxygen-sensitive signaling hub that orchestrates a pathogenic metabolic-epigenetic-trafficking axis. In human asthenozoospermia and murine hypoxia models, CEP162 is aberrantly upregulated and strongly correlates with flagellar malformation and motility loss. Mechanistically, hypoxia inhibits succinate dehydrogenase, driving the accumulation of intracellular succinate, which disrupts the DNA methyltransferase 3-like (DNMT3L)-additional sex combs-like 2 (ASXL2) epigenetic repressor complex in a HIF-independent manner. This triggers CEP162 promoter hypomethylation and transcriptional derepression, establishing a stable epigenetic switch. Functionally, elevated levels of CEP162 act as a dominant negative regulator of ciliary protein delivery: biolayer interferometry and nucleotide exchange assays revealed that CEP162 spatially sequesters Rab1a and noncompetitively inhibits TRAPPC9-mediated GTP loading, thereby uncoupling vesicular trafficking from axonemal cargo delivery. Consequently, TEKT2 is not incorporated into the growing flagellum, leading to microtubule doublet destabilization and structural collapse. Genetic silencing of CEP162 or restoration of DNMT3L/ASXL2 activity fully rescues Rab1a activation, vesicular targeting, and sperm motility. Clinically, sperm CEP162 levels showed promising diagnostic potential in our initial cohort (area under the curve [AUC] >0.9) and were inversely correlated with progressive motility. This study redefines CEP162 from a passive structural component to an active metabolic-epigenetic sensor, reveals a noncanonical mechanism of Rab guanosine triphosphatase (GTPase) regulation and establishes a reversible succinate-driven epigenetic trajectory in environmentally induced infertility. These findings provide a mechanistic framework for metabolic-epigenetic coupling in polarized cells, identify actionable targets for environmentally induced male infertility and highlight a pathogenic axis that may warrant further investigation in the context of broader ciliopathies.

## Introduction

Male infertility affects approximately 7% of men worldwide, with impaired sperm motility (asthenozoospermia) and abnormal sperm morphology (teratozoospermia) being among the most common diagnostic features^1^. Asthenoteratozoospermia, a severe form of male infertility characterized by both reduced sperm motility and structural flagellar defects, often lacks a clear etiology, posing significant challenges for diagnosis and treatment^2^. While genetic mutations in ciliary and flagellar components have been increasingly linked to sperm dysfunction, environmental stressors, particularly hypoxia, are emerging as critical contributors to defective spermatogenesis and impaired sperm quality.

Hypoxia, either pathological (e.g., varicocele or obstructive sleep apnea) 3, 4 or environmental (e.g., high-altitude exposure)^5^, has been consistently associated with impaired male fertility. Clinical and animal studies have demonstrated that chronic or intermittent hypoxia disrupts spermatogenesis, induces oxidative stress, and decreases sperm motility and viability^6^, ^7^. However, the molecular mechanisms by which low oxygen tension translates into structural and functional sperm defects remain incompletely understood. Notably, while hypoxia-inducible factors (HIFs) are well-established mediators of cellular adaptation to low oxygen conditions, recent evidence suggests that epigenetic pathways may play an equally pivotal role in regulating germ cell development under hypoxic stress^8^.

While reactive oxygen species (ROS) have long been considered primary mediators of hypoxic stress, emerging evidence indicates that metabolic reprogramming, particularly the accumulation of tricarboxylic acid (TCA) cycle intermediates, may serve as equally critical and more persistent signaling mechanisms under chronic hypoxia^9^. Notably, succinate, a key TCA metabolite, accumulates when hypoxia inhibits succinate dehydrogenase (SDH) complex activity, leading to reversed electron transport at mitochondrial complex I^10^. Beyond its bioenergetic role, succinate functions as an oncometabolite that competitively inhibits α-ketoglutarate (α-KG)-dependent dioxygenases, including the TET family of DNA demethylases and JmjC-domain histone demethylases, thereby directly reprogramming the epigenetic landscape^11^, ^12^. Intriguingly, recent studies in germ cells have revealed that metabolic‒epigenetic coupling governs critical transitions during spermiogenesis, suggesting that hypoxia-induced succinate accumulation may represent an unexplored link between environmental stress and epigenetic dysregulation in the context of male infertility^13^.

Epigenetic regulation, including DNA methylation and histone modifications, is essential for proper spermatogenesis, particularly during the postmeiotic phase of spermiogenesis when dramatic chromatin remodeling and flagellar assembly occur^14^. DNA methyltransferase 3-like (DNMT3L), a catalytically inactive member of the DNMT3 family, acts as a crucial regulator of de novo DNA methylation in germ cells and has been implicated in transposon silencing and genomic imprinting^15^. DNMT3L loss leads to widespread hypomethylation and sterility in male mice, underscoring its importance in male germ cell development. However, whether and how DNMT3L expression is modulated by environmental cues such as hypoxia have not yet been explored.

Recent studies have highlighted the role of epigenetic scaffolding proteins, such as those in the additional sex combs-like (ASXL) family, in fine-tuning chromatin states during development and disease^16^. In particular, ASXL2 forms complexes with polycomb repressive complexes and modulates histone H2A deubiquitination, influencing gene expression programs that are critical for cell differentiation^17^. Although ASXL1/2 mutations have been studied primarily in hematopoietic malignancies, their potential role in germ cell biology and male fertility has not been investigated.

Importantly, sperm ciliogenesis and flagellar assembly are tightly regulated by a conserved set of proteins localized at the ciliary transition zone (TZ), a gatekeeping structure that controls protein entry into the cilium^18^. Centrosomal protein 162 kDa (CEP162) is a key TZ component that is required for the initiation of ciliogenesis and the recruitment of the intraflagellar transport (IFT) machinery^19^. Mutations in CEP162 have recently been linked to retinal degeneration and ciliopathies, and our preliminary data indicate that CEP162 is aberrantly expressed in patients with asthenoteratozoospermia^20^. However, the upstream regulatory mechanisms governing CEP162 expression, especially under hypoxic conditions, are unknown.

In this study, we integrate clinical observations with *in vitro* and *in vivo* models to investigate the epigenetic regulation of CEP162 under hypoxia and its functional consequences for sperm development and motility. We demonstrate that hypoxia induces CEP162 overexpression through the coordinated silencing of DNMT3L and ASXL2, revealing a novel oxygen-sensitive epigenetic axis. Using CUT&Tag sequencing, coimmunoprecipitation, and functional assays in germ cell lines and patient samples, we show that this DNMT3L/ASXL2-CEP162 pathway disrupts vesicular transport and impairs sperm motility. Our findings reveal a previously unrecognized mechanism linking environmental hypoxia to epigenetic dysregulation and structural defects in sperm, offering new insights into the pathogenesis of asthenoteratozoospermia and potential targets for therapeutic intervention.

## Results

### CEP162 expression is upregulated in clinical patients with asthenozoospermia

Centrosomal and transition zone (TZ) genes are critically involved in asthenoteratozoospermia, a severe form of male infertility marked by both sperm motility and structural defects. Increasing evidence has demonstrated that dysfunction of these evolutionarily conserved components disrupts flagellar assembly and ciliary integrity, directly linking TZ/centrosomal defects to impaired spermatogenesis across species 21, 22. Given this context, we sought to identify dysregulated TZ/centrosomal genes related to human male infertility by performing an integrative analysis of five independent transcriptomic datasets encompassing distinct etiologies: GSE6872 and GSE6969 (teratozoospermia), GSE241326 (nonobstructive azoospermia), and SRP418387 and SRP418442 (asthenozoospermia). Strikingly, while no TZ/centrosomal-related genes were consistently downregulated across all cohorts, 24 genes were robustly and consistently upregulated in every dataset (**Supplementary Table 1, Supplementary Figure 1A-D**). Gene Ontology (GO) and Reactome Gene Set Enrichment Analysis (GSEA) of this commonly upregulated gene set revealed significant enrichment in pathways governing olfactory signaling, cilium assembly and mRNA splicing, with the top five pathways meeting the significance threshold (**Supplementary Table 2, Supplementary Figure 1E**).

To prioritize candidates for mechanistic validation, we applied a multidimensional evaluation framework integrating (i) cross-cohort transcriptional consistency, (ii) established ciliary/centrosomal function, (iii) prior evidence of environmental sensitivity, and (iv) clinical diagnostic potential. Within this framework, CEP162 emerged as a high-priority candidate. Although not the single most statistically extreme hit on the basis of the fold change or P value alone, CEP162 exhibited robust upregulation across all five cohorts and was previously identified by our group as a hypoxia-responsive transcript linked to microtubule dynamics 23. This cross-cohort consistency was validated by meta-analysis, which yielded a pooled P value of 0.0443 (**Supplementary Table 3, Supplementary Figure 1F**), confirming the consistent dysregulation of CEP162 across diverse patient populations. On the basis of these findings, we selected CEP162 for in-depth mechanistic and clinical investigation.

To determine the clinical relevance of CEP162 upregulation, we analyzed a cohort of 55 men (30 with asthenozoospermia and 25 normozoospermic controls). Semiquantitative immunoblotting revealed that CEP162 protein levels were significantly greater in asthenozoospermic patients than in controls (**Figure 1A, Supplementary Table 4**). Importantly, CEP162 expression was significantly negatively correlated with progressive motility (**Figure 1B**) and demonstrated high diagnostic accuracy for distinguishing patients from controls (AUC > 0.9; **Figure 1C**).

Consistent with this rationale, among the sperm samples from asthenozoospermic patients with the highest CEP162 expression, we observed frequent flagellar malformations characterized by the loss of doublet microtubules (**Figure 1D** and **1E**). Immunofluorescence staining revealed that CEP162 colocalized with both proximal and distal centrioles, whereas coimmunoprecipitation assays confirmed the physical interaction between CEP162 and transition zone (TZ) proteins, including MKS5 and NPHP5 (**Supplementary Figure 1G, Figure 1F-I**). Collectively, these findings strongly suggest that upregulated CEP162 expression in clinical asthenozoospermia patients may underlie flagellar structural abnormalities and contribute to impaired spermatogenesis.

### Severe hypoxia induces progressive motility impairment with marked CEP162 upregulation in mice

In this study, we established a normoxia‒hypoxia‒reoxygenation-induced asthenozoospermia mouse model by simulating exposure to an altitude of 5800 m (approximately 11.7% oxygen concentration) for 10 weeks, followed by a recovery period with 21% oxygen for 4 months. We found that the motility of the sperm in both the hypoxic and reoxygenated groups was significantly lower than that in the normoxic control group, as assessed by a computer-assisted sperm analysis (CASA) system. Motility reduction was evident in terms of multiple parameters, including progressive motility, curvilinear velocity (VCL), straight-line velocity (VSL), average path velocity (VAP), linearity (LIN), and straightness (STR) **(Figure 2A)**. Furthermore, we found that the sperm flagella in the hypoxic and reoxygenated groups were prone to morphological abnormalities and exhibited a loss of doublet microtubules (DMTs) **(Supplementary Figure 2A, Figure 2B-C)**. Notably, microtubule regrowth assays in G2 cells revealed that microtubule regrowth capacity was impaired under hypoxic conditions **(Figure 2D-E)**. Consistent with these findings, the levels of biochemical markers of stable microtubules (Ac-Tub and Glu-Tub) were also reduced **(Figure 2F)**, indicating compromised microtubule stability.

Our single-cell transcriptomic analysis revealed significant upregulation of Cep162 mRNA expression in elongating spermatids from rats under hypoxic conditions^23^. In the present study, we demonstrated that hypoxia markedly increased CEP162 protein expression in mouse sperm. Notably, compared with those in the normoxic control group, CEP162 levels in the reoxygenation group remained elevated** (Figure 2F)**. Immunofluorescence analysis of the localization of CEP162 revealed that it localized predominantly to distal/proximal centrioles and physically interacted with the transition zone (TZ) proteins MKS5 and NPHP5, as well as microtubules, under hypoxic conditions **(Supplementary Figure 2B-E)**. Collectively, these data demonstrate that the hypoxia-driven impairment of sperm motility is accompanied by the sustained upregulation of CEP162 expression and the concomitant mislocalization of axonemal components.

### CEP162 overexpression reduces progressive sperm motility in mice

We next investigated the functional significance of CEP162 in sperm motility defects. Using adeno-associated virus-mediated overexpression, we observed that elevated CEP162 expression significantly impaired sperm motility parameters, as measured by computer-assisted sperm analysis (CASA), including progressive motility, curvilinear velocity (VCL), straight-line velocity (VSL), average path velocity (VAP), linearity (LIN), wobble (WOB) and straightness (STR), under normoxic conditions** (Supplementary Figure 3aA-C, Figure 3A)**. Notably, CEP162 overexpression alone recapitulated the motility defects caused by hypoxia, confirming its ability to disrupt sperm function. Morphological analysis revealed more sperm tail deformities and axonemal doublet microtubule (DMT) defects **(Supplementary Figure 3aD, Figure 3B)**. Consistent with these findings, CEP162-overexpressing mice exhibited reduced levels of acetylated α-tubulin (Ac-Tub) and detyrosinated tubulin (Glu-Tub), the established markers of stable microtubules, in isolated flagella after 10 weeks of normoxia (21% O_2_) or hypoxia (11.7% O_2_), indicating that CEP162 induced axonemal microtubule destabilization **(Supplementary Figure 3bF)**. This finding was supported by the impaired microtubule nucleation and regrowth following nocodazole washout in CEP162-overexpressing G2 cells **(Supplementary Figure 3bE, Figure 3C)**. Under hypoxia, CEP162 overexpression did not further worsen most motility parameters, likely because of a floor effect from severe baseline impairment.

To identify the molecular basis of CEP162-induced structural and functional defects, we performed quantitative proteomic profiling of isolated sperm flagella. This revealed significant reductions in the expression levels of TEKT1-5 and EFHC1, the reported core structural and functional components of the axonemal machinery essential for proper ciliogenesis and motility, with TEKT2 showing the most pronounced decrease (lowest log_2_FC and most significant *P* value; **Supplementary Figure 3bI, Figure 3D, Supplementary Table 5**). To validate its microtubule-stabilizing capacity, we found that the purified recombinant TEKT2 protein (**Supplementary Figure 3bG**) significantly suppressed cold-induced microtubule depolymerization *in vitro* under both normoxic and hypoxic conditions, as shown by real-time turbidity assays (**Supplementary Figure 3bH**), demonstrating its stabilizing role. Consistent with the proteomic findings, immunofluorescence analysis confirmed that TEKT2 distribution was markedly reduced within the flagella under hypoxia (**Supplementary Figure 3bJ**). Moreover, TEKT2 mislocalization strongly correlated with DMT disorganization and flagellar bending (**Figures 2B-C, 3B**). Among these factors, TEKT2 was prioritized for in-depth mechanistic investigation because of its most pronounced downregulation, strongest correlation with clinical axonemal defects, and validated microtubule-stabilizing capacity. While multiple TEKT family members are coordinately affected, TEKT2 serves as the optimal representative factor to dissect the broader trafficking blockade.

We therefore determined whether CEP162 overexpression blocks TEKT2 entry into the flagellum. Using an *in vivo* diffusion-barrier assay, we delivered AAV9-RFP-TEKT2-FKBP into mouse testes and analyzed the sperm 4 weeks later. In normoxic controls, RFP-TEKT2-FKBP efficiently incorporated into the flagellum and colocalized with acetylated α-tubulin. In contrast, CEP162 overexpression, under both normoxia and hypoxia, markedly reduced the flagellar entry of RFP-TEKT2-FKBP and increased TEKT2 accumulation in the sperm head (**Figure 3E-G**).

### Silencing CEP162 rescues flagellar malformation in hypoxic mice

Given that CEP162 overexpression impaired the progressive motility of sperm under both normoxic and hypoxic conditions (**Figure 3A**), we next investigated whether silencing CEP162 rescues the flagellar malformations and motility defects induced by chronic hypoxia in mouse sperm. First, we confirmed that testicular in situ injection of CEP162-targeting shRNA effectively reduced CEP162 expression at both the protein and mRNA levels, and modulated related ciliary/centrosomal gene transcription in spermatogenic cells under hypoxia (**Supplementary Figure 4aA-C**). Subsequently, testicular in situ injection of CEP162-targeting shRNA under 11.7% O₂ significantly improved sperm motility parameters, including progressive motility, VCL, VSL, VAP, LIN, WOB, and STR, compared with those in vector shRNA-treated controls (**Supplementary Figure 4bD**). Subsequent morphological assessments indicated that CEP162 knockdown led to a significant reduction in sperm tail deformation rates and defects within DMTs (**Supplementary Figure 4bE, Figure 4A**-**B**). Additionally, we found that CEP162 knockdown under hypoxic conditions led to a significant reduction in the proportions of free tubulin and polymerized microtubule proteins in spermatogenic cells (**Figure 4C**).

Atomic force microscopy (AFM) of flagellar surface mechanics revealed increased rigidity and elasticity in *CEP162*-knockdown mice exposed to hypoxia, indicating fewer structural abnormalities and improved integrity of the flagellar cytoskeleton (**Figure 4D**). Microtubule regrowth assays demonstrated that CEP162 knockdown under hypoxic conditions increased microtubule regrowth capacity in spermatogenic cell lines (**Supplementary Figure 4bF**, **Figure 4E**) and restored the levels of stable microtubule markers in the flagella **(Figure 4G)**. Furthermore, immunofluorescence colocalization analysis revealed that CEP162 knockdown in sperm led to increased colocalization of TEKT2 with both γ-tubulin and pericentrin in the centrosomal region (**Supplementary Figure 4bG**, **Figure 4F**). Notably, we observed increased Tekt2 localization in the sperm flagella of *CEP162*-knockdown mice (**Figure 4G**). Taken together, these findings indicate that CEP162 silencing ameliorates hypoxia-induced flagellar malformations and motility impairment by restoring microtubule stability, increasing the centrosomal colocalization of TEKT2 with γ-tubulin and pericentrin, and promoting proper TEKT2 localization in sperm flagella.

### CEP162 restricts TEKT2 distribution in flagella by impairing Rab1a activation

While CEP162 silencing ameliorates hypoxia-induced flagellar defects by restoring microtubule stability and TEKT2 localization, the molecular mechanisms through which CEP162 regulates the trafficking and spatial distribution of key flagellar proteins remain unclear. Given that CEP162 silencing rescues flagellar integrity and TEKT2 localization under hypoxia (**Figure 4**), we investigated how CEP162 overexpression disrupts the ciliary targeting of TEKT family proteins. We first analyzed its interaction profile. Immunoprecipitation of CEP162 exhibited enhanced binding to these TEKT proteins as well as Rab1a, Rab1b, and Rab14, whereas co-IP of TEKT2 markedly reduced the association with all three Rab1 isoforms **(Supplementary Figure 5A-C, Figure 5A-B)**. These findings suggest that CEP162 sequesters both structural and regulatory proteins into nonfunctional complexes, thereby uncoupling the cargo from the vesicular trafficking machinery required for ciliary targeting.

We next explored the potential mechanisms underlying this trafficking defect. In IMCD3 cells, CEP162 overexpression disrupted TEKT2 localization within cilia, a defect partially rescued by taxol and exacerbated by nocodazole **(Supplementary Figure 5D)**, indicating that CEP162 compromises microtubule stability and impairs cytoskeleton-dependent transport. Furthermore, flow cytometry confirmed a significant decrease in both intracellular and cilia-localized AP2⁺/SSTR3⁺ and Rab1a⁺/SSTR3⁺ vesicles **(Figure 5C-D)**, supporting defective vesicle docking and ciliary delivery. These results indicate that CEP162 overexpression disrupts membrane trafficking and ciliary cargo delivery.

To determine how CEP162 disrupts vesicle dynamics, we evaluated its effect on Rab1a activation. Venn diagram analysis of interactomes revealed that TRAPPC9, a specific guanine nucleotide exchange factor (GEF) for Rab1a, was depleted from the Rab1a complex upon CEP162 overexpression despite not directly interacting with CEP162 **(Figure 5E)**. Coimmunoprecipitation confirmed that TRAPPC9 selectively interacted with Rab1a under nucleotide-free conditions **(Figure 5F-G)**. Biolayer interferometry revealed high-affinity binding between CEP162 and Rab1a, but a competitive ELISA demonstrated that TRAPPC9 did not displace Rab1a from CEP162 **(Supplementary Figure 5E-G)**, suggesting the existence of noncompetitive, likely allosteric or steric, interference rather than direct competition.

Functional assays validated this inhibitory mechanism: TRAPPC9 robustly stimulated [³H]GDP release and [³⁵S]GTPγS loading onto Rab1a, whereas coincubation with CEP162 significantly suppressed both nucleotide exchange activities **(Figure 5H-I)**. These findings confirm that CEP162 impairs TRAPPC9-mediated Rab1a activation. Notably, ciliary metabolite profiling revealed that CEP162 overexpression led to significant reductions in the levels of both ATP and GTP **(Supplementary Figure 5H-I)**, indicating a local energy deficit that further compromised Rab GTPase cycling and vesicle trafficking efficiency.

Taken together, our findings demonstrate that CEP162 acts as a critical negative regulator of ciliary protein trafficking through dual mechanisms: the destabilization of microtubule-based transport and the suppression of Rab1a activation via the allosteric inhibition of TRAPPC9. By sequestering key cargo and disrupting GEF-dependent GTP loading, CEP162 impairs vesicle recycling, reduces ciliary metabolic capacity, and ultimately prevents the proper delivery of axonemal components such as TEKT2. These results position CEP162 as a central modulator of ciliogenesis through the coordinated control of cytoskeletal integrity and membrane trafficking.

### Hypoxia upregulates CEP162 expression via suppression of the DNMT3L-ASXL2 complex

To identify transcriptional regulators of CEP162 in spermatogenic cells under hypoxic stress, we used a dCas9-based chromatin affinity purification approach (CAPTURE) to profile protein interactions at the CEP162 promoter in round spermatids (RS) and elongating spermatids (ES) under normoxia (21% O_2_) and hypoxia (11.7% O_2_). The system utilized an FB-dCas9 fusion protein and sgRNAs targeting the CEP162 promoter, enabling the *in vivo* biotinylation and streptavidin-based pulldown of chromatin-bound complexes **(Figure 6A-B)**. Proteomic analysis revealed distinct protein interactomes under the two different conditions. Proteomic analysis of dCas9-captured interactors revealed that DNMT3L, which functions as a critical regulatory factor that stimulates the catalytic activity of DNMT3A^25^, is a top candidate regulator, with a significant decrease in its enrichment at the CEP162 promoter under hypoxia in both RS and ES **(Supplementary Figure 6A-C, Figure 6C-E)**. Western blotting confirmed that DNMT3L protein levels were markedly reduced in both RS and ES under chronic hypoxia **(Figure 6F)**, suggesting oxygen-dependent regulation of this epigenetic modulator.

We next investigated the functional role of DNMT3L in regulating CEP162 expression. CUT&Tag-seq and ChIP‒qPCR revealed that DNMT3L bound directly to the CEP162 promoter under normoxia; however, this binding was significantly diminished under hypoxia in both RS and ES **(Figure 6G-I, Supplementary Figure 6F)**. EMSAs validated the specific binding of DNMT3L to a region +222 to +251 bp downstream of the transcription start site (TSS), but this binding was abolished under hypoxic conditions **(Supplementary Figure 6D, Figure 6H)**. In Dnmt3l conditional knockout (cKO) mice, which were generated using a floxed allele with exons 6-10 flanked by loxP sites **(Supplementary Figure 6E)**, Cep162 mRNA expression was significantly upregulated in both RS and ES under normoxia and further induced under hypoxia **(Supplementary Figure 6I, Figure 6J)**. Consistent with these findings, the siRNA-mediated knockdown of DNMT3L in G2 cells led to significant induction of CEP162 transcription **(Figure 6K)**, supporting that DNMT3L suppresses CEP162 expression.

Given that ASXL2 has been established to be involved in epigenetic regulation and has been reported to interact with DNMT3L to modulate chromatin states, we examined its functional interplay in the context of CEP162 repression. Co-IP confirmed that DNMT3L and ASXL2 physically interact in G2 cells under both normoxic and hypoxic conditions **(Figure 6L)**, supporting the formation of a repressive complex that may regulate CEP162 expression. ChIP‒qPCR revealed that both DNMT3L and ASXL2 were enriched at the CEP162 promoter under normoxia and that their occupancy was reduced upon knockdown of either protein or under hypoxic conditions **(Figure 6M-O)**. Sequential ChIP (ReChIP) confirmed the cooccupancy of DNMT3L and ASXL2 at the CEP162 promoter under normoxia, which was disrupted by hypoxia or siRNA-mediated knockdown **(Supplementary Figure 6J-K)**, indicating that these proteins function as a complex to maintain CEP162 repression. Moreover, DNMT3L and ASXL2 expression was lower in human teratozoospermic samples than in normozoospermic controls, as determined by analyses of publicly available transcriptomic data from the GEO dataset GSE6969, and this lower expression correlated with poor semen quality **(Supplementary Figure 6G-H)**, underscoring the clinical relevance of these factors in male infertility.

To elucidate the upstream molecular mechanism through which hypoxia suppresses the DNMT3L-ASXL2 complex, we first ruled out canonical hypoxia-inducible factor (HIF) signaling. siRNA-mediated knockdown of HIF-1α, HIF-2α, or both under 1% O_2_ failed to attenuate the downregulation of DNMT3L and ASXL2 or the upregulation of CEP162 (**Supplementary Figure 6L**). Instead, we hypothesized that hypoxia-driven metabolic reprogramming serves as the primary upstream signal. Indeed, treating normoxic cells with dimethyl succinate (DM-Succ, 5 mM), a cell-permeable succinate precursor, fully recapitulated the hypoxia-induced suppression of the DNMT3L/ASXL2 complex and subsequent induction of CEP162 (**Supplementary Figure 6M-O**). Conversely, treating hypoxic cells with dimethyl fumarate (DMF; 100 μM), a prodrug that drives the forward flux of succinate dehydrogenase via mass-action kinetics, effectively attenuated the hypoxia-induced dissociation of the complex and restored CEP162 repression (**Supplementary Figure 6P-R**).

To establish direct mechanistic causality between this metabolic shift and epigenetic remodeling, we further assessed their direct chromatin-level consequences. MeDIP-qPCR revealed that DM-Succ significantly reduced 5-methylcytosine (5-mC) enrichment at the CEP162 promoter, faithfully mimicking the hypoxic phenotype, whereas DMF-mediated succinate clearance robustly restored promoter methylation (**Supplementary Figure 6S**). Furthermore, ChIP‒qPCR demonstrated that DM-Succ treatment significantly decreased the chromatin occupancy of both DNMT3L and ASXL2 at the CEP162 promoter under normoxic conditions. Crucially, DMF treatment under hypoxia successfully restored the recruitment of the DNMT3L-ASXL2 repressor complex to chromatin (**Supplementary Figure 6P-Q**).

Collectively, these data establish a direct mechanistic link: hypoxia suppresses the DNMT3L-ASXL2 complex through a HIF-independent, succinate-driven metabolic-epigenetic pathway. Specifically, intracellular succinate accumulation induces the dissociation of the DNMT3L-ASXL2 repressor complex from *CEP162* promoter chromatin, which subsequently drives CEP162 promoter hypomethylation, thereby derepressing *CEP162* transcription.

### Activation of DNMT3L/ASXL2 effectively rescues the CEP162-mediated impairment of vesicular transport under hypoxia

To investigate whether activation of DNMT3L or ASXL2 rescues hypoxia-induced defects in vesicular trafficking, we first assessed clathrin-coated vesicle dynamics in IMCD3 cells under hypoxic conditions. Flow cytometry analysis revealed that compared with the *in vitro* control, the overexpression of DNMT3L-3×Flag or ASXL2-3×Flag significantly increased the proportion of Clathrin/SSTR3 double-positive cells and cilia-localized vesicles, indicating increases in vesicle docking and ciliary targeting **(Supplementary Figure 7A-E, Figure 7A)**. Similarly, Rab1a/SSTR3 colocalization analysis demonstrated that overexpression of either DNMT3L or ASXL2 restored Rab1a-positive vesicle trafficking to the primary cilium **(Supplementary Figure 7F, Figure 7B)**, which is consistent with functional recovery of the vesicular transport machinery.

To elucidate the molecular mechanism underlying the rescue of vesicular transport, we examined the activation status of Rab1a using a GST pull-down assay with full-length TRAPPC9, which functions as a guanine nucleotide exchange factor (GEF) for Rab1a within the TRAPP II complex. Lysates from G2 cells overexpressing DNMT3L-3×Flag or ASXL2-3×Flag under hypoxia presented significantly higher levels of active Rab1a-GTP in the pull-down fraction than the lysates from G2 cells in the *in vitro* control group did **(Figure 7C)**. These findings indicate that the activation of DNMT3L or ASXL2 restores the GEF activity of TRAPPC9 toward Rab1a, thereby reactivating the Rab1a-dependent vesicular transport pathway that is impaired under hypoxic stress.

We next evaluated the expression of CEP162, the key downstream effector, at both the protein and transcriptional levels. Western blot analysis demonstrated that CEP162 protein levels were markedly elevated in hypoxic control mouse round spermatids, whereas the overexpression of DNMT3L-EGFP or ASXL2-3×Flag led to a significant reduction in CEP162 expression **(Figure 7D)**. Consistent with these findings, in G2 cells, the overexpression of both DNMT3L-3×Flag and ASXL2-3×Flag under hypoxia resulted in the pronounced downregulation of CEP162 protein expression **(Figure 7E)**. ChIP‒qPCR revealed increased enrichment of 5-methylcytosine (5-mC) at the CEP162 promoter in the DNMT3L-3×Flag and ASXL2-3×Flag groups** (Figure 7F)**, indicating that DNMT3L/ASXL2 activation promotes DNA methylation-mediated epigenetic silencing of CEP162 under hypoxia.

At the transcriptional level, qRT‒PCR confirmed that CEP162 mRNA expression was upregulated under hypoxia in the controls but was significantly suppressed upon overexpression of DNMT3L or ASXL2 in both mouse round spermatids and G2 cells **(Supplementary Figure 7G-H)**. ChIP‒qPCR confirmed the enhanced binding of both DNMT3L and ASXL2 to the CEP162 promoter upon overexpression, supporting their direct role in transcriptional regulation **(Supplementary Figure 7I-J)**. Furthermore, combined immunofluorescence and single-molecule FISH (IF/smFISH) codetection of CEP162 pre-mRNA (intron and exon probes) and the CEP162 protein revealed a coordinated decrease in both nascent transcription and mature mRNA signals in cells overexpressing DNMT3L or ASXL2 **(Supplementary Figure 7K-L, Figure 7G)**, a finding that is consistent with transcriptional repression rather than posttranscriptional stabilization.

To rigorously elucidate the mechanistic dependency of CEP162-mediated defects on Rab1a inactivation and TEKT2 deprivation *in vivo*, we performed targeted genetic rescue experiments. First, we utilized a constitutively active Rab1a mutant (Rab1a-Q70L), which is locked in the GTP-bound state and does not depend on GEF (TRAPPC9) activity. Testicular in situ injection of AAV9-Rab1a-Q70L into CEP162-overexpressing mice significantly restored the proper distribution of TEKT2 within the sperm flagella and the microtubule regrowth capacity (**Supplementary Figure 7M-N**). Crucially, wild-type Rab1a failed to achieve this response, providing definitive *in vivo* evidence that restoring Rab1a-GTP signaling is sufficient to bypass CEP162-mediated inhibition. Furthermore, to establish a direct causal link between impaired TEKT2 delivery and flagellar structural collapse, we co-overexpressed TEKT2 in CEP162-overexpressing mice via AAV9. This massive replenishment of the axonemal cargo significantly ameliorated the pathological phenotypes, effectively restoring progressive sperm motility and axonemal integrity (**Supplementary Figure 7O-P**). These *in vivo* rescue data conclusively demonstrate that CEP162 acts as a dominant negative signaling hub and that the restoration of Rab1a activity and subsequent TEKT2 delivery is sufficient to reverse downstream structural and functional defects. Together, these results demonstrate that the activation of DNMT3L or ASXL2 effectively rescues the hypoxia-induced impairment of vesicular transport by epigenetically silencing CEP162 and restoring TRAPPC9-mediated Rab1a activation. Critically, the schematic model integrates these findings into a unified epigenetic-trafficking axis: under normoxia, the DNMT3L-ASXL2 complex maintains CEP162 promoter methylation and transcriptional repression, permitting unimpeded TRAPPC9-Rab1a-GTP signaling for TEKT2 delivery and flagellar assembly; under hypoxia, the dissociation of this repressor complex triggers CEP162 derepression, after which CEP162 noncompetitively sequesters Rab1a at the basal body and sterically inhibits TRAPPC9 GEF activity, thereby uncoupling vesicular trafficking from axonemal cargo delivery and inducing microtubule instability and flagellar malformation, establishing CEP162 not only as a structural component but also as a central oxygen-sensitive epigenetic sensor and signaling hub whose dysregulation directly links environmental stress to male infertility **(Figure 7H)**.

## Discussion

The transformation of round spermatids into elongated spermatids represents a critical phase in spermatogenesis characterized by extensive cytoskeletal remodeling and the precise assembly of the flagellar axoneme. Our study established a comprehensive pathogenic axis that links environmental hypoxia to asthenoteratozoospermia through the epigenetic dysregulation of CEP162, a ciliary transition zone (TZ) protein previously implicated in axonemal DMT defects and microtubule organization. Although the results of the transcriptomic meta-analysis revealed multiple genes whose expression was coupregulated in the centrosomal and transition zone (TZ), including NEK4, CEP162 was prioritized on the basis of three convergent findings: (i) it exhibited a statistically significant negative correlation with progressive sperm motility; (ii) it demonstrated high diagnostic accuracy for pathological conditions; and (iii) its ectopic overexpression alone was sufficient to recapitulate key pathological phenotypes, including TEKT2 mislocalization and axonemal ultrastructural defects. While CEP162 has been well established as a central structural scaffold at the ciliary gate, orchestrating the assembly and function of TZ components to ensure proper ciliogenesis and ciliary maintenance^26^-^28^, our findings reveal a previously unrecognized, nonstructural role for CEP162 as a signaling hub that governs intracellular trafficking. We demonstrate that hypoxia-induced suppression of the DNMT3L-ASXL2 complex leads to CEP162 promoter hypomethylation and transcriptional derepression, culminating in aberrant CEP162 accumulation. This overexpression disrupts the spatiotemporal regulation of Rab1a-dependent vesicular transport, impairs the ciliary delivery of axonemal components such as TEKT2, and ultimately compromises flagellar ultrastructure and sperm motility.

Importantly, probe-level discordance was found for CEP162 in the GSE6872/GPL570 dataset, where the 3'-proximal representative probe (206006_s_at) indicated upregulation, whereas the more 5'-distal probe (206005_s_at) suggested downregulation. This observation likely reflects platform-specific technical factors, including differential hybridization efficiency, transcript isoform coverage, or 3'-end amplification bias inherent to Affymetrix 3' IVT chemistry, rather than biological contradiction. By applying predefined, direction-independent selection criteria (RefSeq exemplar annotation and 3' proximity), we minimized the risk of selection bias while transparently reporting probe-level heterogeneity. The consistent positive direction of the representative probe across all five harmonized datasets, together with the positive random-effects pooled estimate from the meta-analysis, supports CEP162 as a cross-dataset upregulated candidate despite substantial between-study heterogeneity (I² = 96.2%). Such heterogeneity is expected in an integrated analysis combining distinct technologies (Affymetrix/Illumina microarrays and RNA-seq), normalization strategies, and independent clinical cohorts. Our use of a random-effects model explicitly accommodates such variability, allowing the true effect size to vary across studies while estimating the average direction of effect.

A pivotal finding of our work is the noncanonical regulation of Rab1a by CEP162. Traditionally, the activation of Rab GTPases is governed by guanine nucleotide exchange factors (GEFs), such as TRAPPC9, which catalyze GDP-to-GTP exchange—a process fundamental to membrane trafficking that is tightly controlled in both space and time to ensure precise Rab activity within specific cellular compartments^29^. GEFs such as TRAPPC9 achieve substrate specificity and membrane targeting through structural adaptations that allow recognition of distinct organelle membranes and selective activation of cognate Rabs, thereby maintaining the fidelity of intracellular transport^30^. Our data revealed that CEP162 overexpression impaired the TRAPPC9-mediated loading of GTP onto Rab1a; however, biolayer interferometry (BLI) revealed that CEP162 and TRAPPC9 did not compete for Rab1a binding. This lack of competitive binding is not a technical artifact but a biologically significant observation, suggesting a spatially segregated, noncompetitive regulatory mechanism. We propose a model in which CEP162 acts as a membrane-tethered scaffold that sequesters Rab1a-GDP at the basal body, thereby suppressing its activation without altering total Rab1a or Clathrin protein levels or their overall subcellular distribution. Consequently, general vesicle markers such as Rab1a and Clathrin remained detectable in both the cell body and cilia under basal hypoxia, explaining the lack of apparent relocalization in untransfected cells. In contrast, cargo proteins such as TEKT2, whose ciliary targeting is highly dependent on Rab1a-GTP, exhibit early and pronounced mislocalization, making them sensitive reporters of trafficking stress even when the bulk trafficking machinery appears intact.

This “molecular sink” function was supported by proteomic and coimmunoprecipitation analyses, which revealed that CEP162 directly interacts with Rab1a, Rab1b, Rab14, and multiple TEKT family proteins. Notably, CEP162 overexpression enhances its binding to these cargoes while simultaneously reducing their association with Rab1a, effectively trapping both regulatory GTPases and structural components in nonproductive complexes and uncoupling vesicle transport from cargo delivery. This sequestration mechanism aligns with the fundamental principles of vesicle trafficking, where the spatiotemporal regulation of GTPase activity and cargo recognition is critical for fidelity. For instance, GTP hydrolysis by GAPs occurs prior to vesicle formation and is essential for proper cargo sorting, indicating that the dysregulation of GTPase dynamics can directly impair cargo selection^31^. Furthermore, structural studies have shown that coat protein complexes, such as coatomers, can physically sequester cargo molecules, preventing premature interactions, a process analogous to the proposed sink function^32^, ^33^. The formation of such stable, nonproductive complexes by CEP162 may act as a decoy mechanism, effectively diverting key components such as Rab1a and TEKT2 away from the functional vesicular transport machinery. The functional consequence is a failure to deliver critical axonemal building blocks, such as TEKT2, to the growing flagellum, leading to microtubule doublet defects and flagellar malformation.

Importantly, while our study focused on TEKT2 as a representative axonemal cargo, our proteomic data revealed that CEP162 overexpression coordinately reduced the expression of multiple TEKT family members (TEKT1, TEKT3, TEKT4, and TEKT5) in addition to TEKT2 (Figure 3D, Supplementary Figure 3bI). This broad impact on the TEKT family likely reflects the general nature of blockade of Rab1a-dependent trafficking rather than cargo-specific effects. TEKT proteins function cooperatively as heteromeric complexes within axonemal microtubule inner proteins (MIPs), where they collectively stabilize doublet microtubules through lateral interactions^34^. The simultaneous reduction in the expression of multiple TEKTs likely results in synergistic destabilization of the axonemal architecture, amplifying the structural defects beyond what would be expected from TEKT2 loss alone. Future studies employing a systematic functional screen of individual TEKT family members, potentially using CRISPR-based knockout approaches or cargo-specific trafficking assays, will be essential for dissecting the relative contributions of different TEKTs to CEP162-mediated flagellar defects and identifying potential therapeutic targets that could restore axonemal integrity. Nevertheless, TEKT2 serves as an excellent sentinel marker for broader trafficking dysfunction, given its pronounced dysregulation, strong correlation with structural defects, and experimentally validated role in microtubule stabilization.

Our investigation into the downstream effects of disrupted vesicular transport reveals a novel link between membrane dynamics and cytoskeletal integrity. We observed that CEP162 overexpression reduced microtubule stability, as evidenced by increased soluble tubulin levels and impaired aster regrowth after nocodazole washout (Figure 4E). However, CEP162 does not directly bind or cleave tubulin, indicating that microtubule destabilization is a secondary consequence of defective cargo delivery. This highlights a hierarchical organization in flagellar assembly: proper vesicular trafficking must precede and support cytoskeletal remodeling. This principle is conserved across ciliated cells, where actin and the microtubule cytoskeletons rely on coordinated vesicular transport, particularly from the Golgi apparatus, for the timely delivery of structural and functional components to the ciliary base^35^. The actin cytoskeleton itself plays a key role in mediating membrane trafficking events essential for ciliogenesis, underscoring that cytoskeletal remodeling is contingent upon the establishment of efficient trafficking pathways^36^. Furthermore, temporal studies of ciliary dynamics have shown that structural disassembly events are preceded by specific trafficking and organizational cues, reinforcing the sequential nature of this process^37^. In this context, CEP162 functions as a gatekeeper of compartmentalized transport, ensuring that structural proteins are delivered in a coordinated manner to support axonemal elongation.

Our identification of succinate as a hypoxia-induced epigenetic modulator extends the canonical ROS-centered paradigm of hypoxic signaling. While acute hypoxia may trigger transient ROS bursts, chronic oxygen deprivation promotes metabolic adaptation, wherein succinate accumulation provides a stable, concentration-dependent signal capable of persisting beyond the hypoxic episode, a feature that may explain the “epigenetic memory” observed in reoxygenated sperm (Figure 2F). The competitive inhibition of α-KG-dependent dioxygenases by succinate represents a direct biochemical link between mitochondrial metabolism and chromatin regulation, positioning the α-ketoglutarate/succinate ratio as a tunable rheostat for epigenetic plasticity in germ cells^38^, ^39^. Critically, our MeDIP-qPCR data (Supplementary Figure 6S), combined with ChIP‒qPCR evidence (Supplementary Figures 6P-6Q), delineate the precise sequence of this epigenetic switch. This succinate-driven metabolic shift triggers the dissociation of the DNMT3L-ASXL2 repressor complex from *CEP162* promoter chromatin. As a direct consequence of the loss of this epigenetic scaffolding machinery, the local chromatin landscape undergoes promoter hypomethylation, converting transient metabolic stress into a stable, transcriptionally active epigenetic state that drives pathological CEP162 overexpression. Notably, this mechanism operates orthogonally to HIF signaling, as confirmed by our siRNA experiments (Supplementary Figure 6L), and may account for the HIF-independent phenotypes reported in other hypoxia-associated infertility models^40^, ^41^.

The resistance of specific chromatin modifications to reprogramming suggests that the hypoxia-induced disruption of the DNMT3L-ASXL2 complex and the consequent loss of repressive marks (e.g., DNA methylation and H3K27me3) at the CEP162 promoter may represent a similarly stable, and potentially irreversible, epigenetic switch in male germ cells. Mechanistically, we revealed that the DNMT3L-ASXL2 complex acts as a critical oxygen-sensitive repressor of CEP162. Under normoxia, this complex binds to the CEP162 promoter and maintains a repressive chromatin state, likely through H3K27me3 deposition and DNA methylation. Hypoxia disrupts this complex, leading to promoter demethylation and transcriptional activation. This places CEP162 as a central node in an epigenetic sensor network that translates redox imbalance into structural cellular dysfunction.

The epigenetic regulation of CEP162 through DNMT3L and ASXL2 represents another novel aspect of our findings. While DNMT3L is known to be involved in de novo DNA methylation, functioning as a critical regulatory factor that stimulates the catalytic activity of DNMT3A through direct interaction and links histone modifications (such as unmodified H3K4) to DNA methylation^25^, it has not been previously implicated in the repression of centrosomal or ciliary genes. Our CUT&Tag-seq data revealed that DNMT3L and ASXL2 cooccupy the CEP162 promoter region, with hypoxia significantly reducing their binding occupancy (Figure 6I, 6M and 6N; Supplementary Figure 6G). This hypoxia-induced dissociation allows for aberrant CEP162 transcription, ultimately leading to protein overexpression that disrupts microtubule stability. The clinical relevance of this pathway is underscored by our analysis of a GEO dataset (GSE6969), which revealed significant downregulation of DNMT3L and ASXL2 expression in teratozoospermic patients.

Interestingly, the results of our sequential ChIP‒qPCR experiments demonstrated that the relationship between DNMT3L and ASXL2 is interdependent at the CEP162 promoter (Supplementary Figure 6K and 6L). Knockdown of either protein reduced the chromatin occupancy of both factors (Figure 6M and 6N), suggesting the formation of a cooperative complex rather than a hierarchical, sequential recruitment mechanism. This mutual dependency contrasts with the canonical roles of DNMT3L in recruiting DNMT3A/3B or of ASXL2 in stabilizing PRC2, where the loss of the recruited component typically does not impair the binding of the recruiter. In those well-established paradigms, DNMT3L acts as an upstream facilitator that increases de novo methylation by recruiting DNMT3A/3B without requiring its own chromatin association^42^, whereas ASXL2 functions after recruitment to stabilize PRC2 integrity and optimize H3K27me3 activity without being essential for initially targeting the complex^43^. Instead, our data indicate that DNMT3L and ASXL2 function as obligate repressive units at the CEP162 locus, potentially enabling a highly sensitive, switch-like epigenetic response to hypoxic stress. This cooperative mode of action may represent a spermatid-specific regulatory mechanism that ensures the robust silencing of ciliogenesis-related genes during normal flagellar assembly, with its disruption under hypoxia leading to pathological CEP162 overexpression and subsequent defects in sperm motility. While HIF-regulated prolyl hydroxylases (PHDs) have been implicated in the regulation of the stability of other centrosomal proteins (e.g., CEP192)^44^, our data indicate that CEP162 is regulated primarily at the transcriptional level via DNA methylation. The dissociation of HIF signaling from the DNMT3L-ASXL2 axis in our siRNA experiments (Supplementary Figure 6L) further supports this epigenetic mechanism over posttranslational stability control.

The implications of our findings extend beyond male infertility. The DNMT3L-ASXL2-CEP162-Rab1a axis may represent a conserved pathway for sensing metabolic stress in ciliated cells. DNMT3L, although catalytically inactive, plays crucial roles in genomic imprinting and retrotransposon silencing during gametogenesis^45^. It functions as a key adaptor protein that recruits and stimulates catalytically active methyltransferases such as DNMT3A to establish de novo DNA methylation at imprinted germline differentially methylated regions (gDMRs) and transposable elements, thereby ensuring epigenetic fidelity and genome stability^15^. The loss of DNMT3L leads to widespread hypomethylation, aberrant transposon activation, and sterility in male mice, highlighting its indispensable role in germ cell development^15^, ^46^. The specialized function of DNMT3L in germline epigenetic programming, which is distinct from that in somatic cells, is further evidenced by its interaction with histone modifications such as H3K9 methylation, which are critical for chromatin remodeling and meiotic progression^47^. Its sensitivity to hypoxic conditions suggests a broader role in environmental adaptation, where it may act as a sentinel for redox imbalance, dynamically modulating the epigenetic landscape in response to stress. Similarly, TRAPPC9 has been linked to neurodevelopmental disorders, and Rab1a is essential for ER-Golgi trafficking. TRAPPC9 functions as a subunit of the TRAPPII complex, a conserved oligomeric guanine nucleotide exchange factor (GEF) that activates Rab1a to regulate vesicular transport from the ER to the Golgi and intra-Golgi trafficking, processes that are fundamental to protein sorting and secretory pathway integrity^48^, ^49^. Disruptions in this pathway, such as those induced by TRAPPC9 mutations, are associated with neurodevelopmental abnormalities, underscoring the critical importance of precise Rab1a activation in neuronal development and function^50^. Our work positions CEP162 as a context-specific modulator of this fundamental trafficking machinery, particularly in polarized cells with high secretory demands, such as spermatids and neurons.

The negative result from the BLI competition assay, while initially unexpected, provides a compelling argument for the complexity of Rab regulation in specialized cellular compartments. Unlike soluble GTPases, ciliary Rabs are subject to stringent spatial control, functioning as markers to identify distinct membrane domains and organizing specific effector complexes to ensure fidelity in ciliary trafficking and signaling^51^-^53^. This spatial precision is essential for processes such as Hedgehog signaling and intraflagellar transport (IFT), where Rabs such as Rab23 and IFT27 govern cargo entry, exit, and signal transduction within the cilium^54^. Their localization and activity are further fine-tuned by the IFT machinery, the exocyst complex, and posttranslational modifications such as phosphorylation, all of which contribute to the maintenance of ciliary composition and functional integrity^55^, ^56^. Our data suggest that CEP162 imposes an additional layer of regulation beyond the classical GEF-GAP cycle, possibly through phase-separated condensates or lipid raft partitioning at the transition zone. Future studies using superresolution imaging (e.g., STORM) and *in vitro* reconstitution assays will be essential for visualizing the spatial organization of the CEP162-Rab1a-TRAPPC9 triad.

While our study focused primarily on RAB1A, the central GTPase impaired by CEP162 overexpression, it is conceivable that other RAB family members that regulate vesicular trafficking, particularly those involved in intraflagellar transport (IFT), may also be affected, and the partial rescue of microtubule stability upon the reactivation of DNMT3L or ASXL2 suggests that additional hypoxia-sensitive pathways may contribute to cytoskeletal disorganization. This possibility is supported by the established role of epigenetic regulators such as DNMT3L and ASXL2 in orchestrating chromatin states in response to environmental stress, where their dysregulation can lead to widespread alterations in gene expression programs that govern cellular structure and function. The complexity of protein‒protein interaction networks, as revealed by interactome studies, suggests that DNMT3L and ASXL2 may interact with broader signaling cascades beyond the CEP162-Rab1a axis, potentially influencing cytoskeletal dynamics through interconnected pathways involving histone modifications, transcriptional regulation, and metabolic sensing^57^. Notably, our functional rescue experiments highlight the therapeutic promise of targeting DNMT3L/ASXL2-CEP162-RAB1A axis, as the genetic silencing of CEP162 or restoration of DNMT3L/ASXL2 activity in hypoxic models effectively reinstates RAB1A-GTP levels, rescues the ciliary localization of critical axonemal components such as TEKT2, and restores sperm motility, indicating that hypoxia-induced spermatogenic defects are reversible at the molecular level during early spermiogenesis; these findings not only lay a foundation for novel therapeutic strategies for asthenoteratozoospermia but also open avenues for developing precision interventions such as small-molecule inhibitors that disrupt the CEP162-RAB1A interaction or pharmacological agents that stabilize the DNMT3L-ASXL2 complex, offering targeted correction of the underlying pathogenic cascade of male infertility linked to hypoxic stress.

Despite these compelling findings, our study has several limitations that warrant careful consideration. First, while our initial clinical cohort (n = 55) demonstrated the promising diagnostic potential of sperm CEP162 levels (AUC > 0.9), this sample size is insufficient to establish definitive clinical utility or generalizability. Future multicenter studies involving larger, independent, and ethnically diverse patient cohorts are essential to rigorously validate the sensitivity, specificity, and predictive value of CEP162 as a diagnostic biomarker for asthenoteratozoospermia. Second, while our mechanistic insights into CEP162-driven trafficking defects may have broader implications for ciliopathies, given the established role of CEP162 as a ciliary transition zone gatekeeper, our current model specifically addresses environmentally induced (hypoxia-driven) epigenetic dysregulation in male germ cells. Direct extrapolation of these findings to genetic ciliopathies requires dedicated investigation in relevant disease-specific models. Finally, while our rescue experiments strongly support the proposed axis, the translation of these findings into targeted pharmacological interventions (e.g., small-molecule stabilizers of the DNMT3L-ASXL2 complex) remains a goal for future preclinical development.

## Conclusions

We identified a novel pathogenic cascade in which environmental hypoxia inhibits succinate dehydrogenase (SDH) activity, driving intracellular succinate accumulation that functionally disrupts the DNMT3L-ASXL2 epigenetic repressor complex through metabolic-epigenetic crosstalk. This metabolic-epigenetic switch triggers promoter hypomethylation and the transcriptional derepression of CEP162. The resulting aberrant overexpression of CEP162 in spermatids functions not only as a structural scaffold but also as a dominant negative signaling hub that spatially sequesters Rab1a-GDP to the basal body, thereby impairing TRAPPC9-mediated GTP loading and disrupting Rab1a-dependent vesicular trafficking. This consequently prevents the ciliary delivery of critical axonemal components, notably TEKT2, culminating in microtubule instability, flagellar malformation, and ultimately asthenoteratozoospermia. This work redefines CEP162 as a pivotal integrator of metabolic, epigenetic, and trafficking signals essential for faithful flagellar assembly, provides a mechanistic framework for environmentally induced male infertility, and highlights a reversible pathogenic axis that may offer new perspectives for future research into idiopathic asthenozoospermia and related ciliopathies.

## Materials and Methods

### Research ethics

All clinical procedures involving human participants were authorized by the Institutional Ethics Committee of the Second Affiliated Hospital of Army Medical University (Approval No. 2025-135-01) and strictly adhered to the ethical principles of the Declaration of Helsinki. Before enrollment, written informed consent was secured from every individual. Additionally, all animal protocols received ethical clearance from the Institutional Animal Care and Use Committee of the Army Medical University (Permit No. AMUWEC20230266). The care and handling of laboratory animals strictly complied with the National Institutes of Health Guide for the Care and Use of Laboratory Animals (NIH Publication No. 8023, revised 1978).

For human sample collection, 55 individuals, 30 patients diagnosed with asthenozoospermia and 25 men with normozoospermic parameters, were included. Semen samples were processed and evaluated following the most recent World Health Organization (WHO) criteria to ensure standardized assessment of sperm concentration, motility, and morphology.

### Human sperm sample collection and cohort definition

Semen samples were collected from 55 male participants aged 22-40 years who were recruited at the Department of Urology, the Second Affiliated Hospital of Army Medical University, Chongqing, China. All participants provided written informed consent, and the study was approved by the Medical Ethics Committee of the Second Affiliated Hospital of Army Medical University (Approval Number: 2025-135-01).

Participants were classified into two groups on the basis of the WHO 2021 criteria and clinical diagnosis: normozoospermic controls (n = 25): those with progressive motility (PR) ≥ 32%, normal sperm concentration and morphology, and no history of infertility, urogenital infection, varicocele, hormonal disorders, or exposure to gonadotoxic agents. Patients with asthenozoospermia (n = 30): those with a PR < 32% with otherwise normal concentration and morphology for which known genetic causes (e.g., Y chromosome microdeletions and CFTR mutations) were excluded.

Additional exclusion criteria for both groups included smoking, alcohol abuse (> 20 g/day), obesity (BMI > 30), fever within the past 3 months, or residence at high altitude (> 1,500 m).

Semen analysis was performed within 1 hour of liquefaction using computer-assisted sperm analysis (CASA). For Western blotting and molecular assays, sperm were purified by Percoll gradient centrifugation, and protein lysates were prepared as described. The clinical and demographic characteristics of the participants are summarized in Supplementary Table 4.

### Mice and rats

To establish the chronic hypoxia model, rodents (both rats and mice) were housed in a specialized hypobaric chamber that mimicked a high-altitude environment of 5,800 meters. In this setup, the ambient air retained a normal oxygen fraction (~21%), but the overall barometric pressure was lowered, thereby reducing the partial pressure of oxygen. Age-matched control groups were kept at a baseline elevation of 300 meters under standard normobaric conditions. Before the hypoxic intervention, all animals underwent an 8-week acclimatization period in the facility. They were provided with unrestricted access to water and standard chow. The vivarium environment was strictly regulated, maintaining a 12-hour light/12-hour dark photoperiod, a constant temperature range of 22-24 °C and humidity levels between 40% and 60%.

The generation of mice lacking *DNMT3L* specifically in the germline (project no: CKOAIP250808LL1) was commissioned from Cyagen Biosciences (Suzhou, China). This was achieved by utilizing a tamoxifen-dependent Cre-loxP recombination strategy. Specifically, *Stra8-iCreERT2* transgenic mice were mated with *DNMT3L*-floxed mice (C57BL/6J background), in which loxP sequences were inserted to flank exons 6 through 10 of the target gene. Offspring genotyping was conducted using polymerase chain reaction (PCR) assays.

Tamoxifen treatment protocol: To trigger targeted gene excision in post-meiotic germ cells, 8-week-old male mice harboring both the *Stra8-iCreERT2* and *DNMT3L flox/flox* alleles were administered tamoxifen (Sigma-Aldrich, Cat# T5648) via intraperitoneal injection for 5 successive days. The drug was freshly compounded by dissolving it in sterile corn oil (Aladdin, Cat# C116023) to yield a 10 mg/mL solution, which was thoroughly vortexed. The daily dosage was set at 1 mg per mouse (approximating 40 mg/kg based on a 25 g average body mass). To prevent degradation, the working solutions were shielded from light and kept at 4 °C, being used within 24 h of preparation. Following the last dose, the animals were kept in solitary confinement for 1 day to clear the unmetabolized drug before resuming normal housing and commencing the hypoxia regimen. This specific temporal induction window was chosen to selectively ablate *DNMT3L* in differentiating spermatocytes and post-meiotic stages, thereby circumventing the early meiotic block typically caused by global *Dnmt3l* deficiency^58^.

### Cell culture, hypoxia treatment and transfection

Murine spermatogonia (GC-1), pachytene spermatocytes (GC-2), and inner medullary collecting duct (IMCD3) cells were purchased from Procell Life Science & Technology (Wuhan, China). These cell lines were routinely maintained in Dulbecco's Modified Eagle Medium (DMEM) enriched with 10% fetal bovine serum (FBS). To simulate pathological hypoxia comparable to clinical conditions like varicocele or testicular torsion, cells were exposed to 1% O_2_, a level known to drop below physiological baselines (typically 1-2% O_2_ in the testis) 59. While 21% O_2_ served as the standard normoxic control to match conventional *in vitro* paradigms^60^-^62^, the hypoxic environment (comprising 1% O_2_, 5% CO_2_, and 94% N_2_) was precisely regulated at 37 °C within a modular hypoxia workstation (Invivo_2_, Baker Ruskinn). Crucially, to prevent artifactual reoxygenation, all downstream processing, including RNA and protein isolation, was strictly conducted within the hypoxic chamber.

For *in vitro* hypoxia modeling, GC-1 and GC-2 cells were initially plated at a density of 5 × 10^5^ cells per well in 35-mm 6-well plates and incubated under standard normoxic conditions for 24 hours. Following a single PBS wash, the cultures were relocated to the hypoxia workstation for a 36-hour duration. This specific 1% O_2_ exposure window was determined via preliminary experiments to optimally trigger hallmark hypoxic responses, such as HIF-1α accumulation and DNMT3L suppression, thereby faithfully recapitulating the transcriptional adaptations observed during chronic *in vivo* hypoxia.

Transient gene silencing of *Dnmt3l* and *Asxl2* was achieved using specific small interfering RNA (siRNA) duplexes. The targeted sequences were: *Dnmt3l* (sense: 5'-GGAUGACCAAGAGACAACUTT-3', antisense: 5'-AGUUGUCUCUUGGUCAUCCTT-3') and *Asxl2* (sense: 5'-GCCCAAAGCAGGUUCUAAUTT-3', antisense: 5'-AUUAGAACCUGCUUUGGGCTT-3'). A scrambled, non-targeting siRNA (sense: 5'-UUCUCCGAACGUGUCACGUTT-3', antisense: 5'-ACGUGACACGUUCGGAGAATT-3') functioned as the negative control. Cellular transfections were executed utilizing Lipofectamine 3000 reagent (Invitrogen) strictly following the vendor's protocol, with knockdown efficacy verified 48 hours post-transfection.

To achieve *in vivo* upregulation of ASXL2, we developed a recombinant adeno-associated virus serotype 9 (AAV9) construct. This vector harbored the complete murine ASXL2 coding sequence (accession NM_001270988), which was fused in-frame to a 3×Flag epitope and driven by the enhanced short elongation factor-1α (EFS) promoter. The synthetic ASXL2 gene, derived from NCBI reference data, was integrated into the pAAV2-EFS-pA plasmid backbone (Taitool Bioscience, Shanghai, China). This backbone features a truncated EFS promoter (~250 bp) and a compact synthetic polyadenylation signal (48 nt). The final assembled plasmid, named pAAV2-EFS-mASXL2-3×Flag-pA, possessed an insert length of roughly 4.3 kb. Subsequent viral packaging and purification, executed by Taitool Bioscience, yielded AAV9 particles with a robust titer exceeding 2.2 × 10^13^ vector genomes (v.g.)/mL.

For the ectopic expression of murine DNMT3L (accession NM_001081695.2), the corresponding coding region was ligated into a CMV-MCS-3×Flag-WPRE-BGH polyA vector. This specific plasmid utilizes a cytomegalovirus (CMV) immediate-early promoter for robust, constitutive transcription, coupled with a multiple cloning site (MCS) and a C-terminal 3×Flag tag to facilitate downstream detection and immunoprecipitation. To enhance mRNA stability and translational efficiency, the construct incorporates a woodchuck hepatitis virus posttranscriptional regulatory element (WPRE) alongside a bovine growth hormone (BGH) polyadenylation signal. Furthermore, the entire expression cassette is bordered by AAV2 inverted terminal repeats (ITRs), supplying the necessary cis-acting sequences for viral replication and packaging. Co-transfection of this recombinant plasmid, alongside the pHelper and pAAV-RC9 (serotype 9) packaging plasmids, was performed in AAV-293 cells. Viral assembly occurred over 48-72 hours, after which both cellular lysates and culture supernatants were collected. The crude viral preparations were subsequently concentrated and purified via iodixanol gradient or column chromatography to optimize purity and yield. Quantitative PCR (qPCR) targeting the viral genome confirmed final titers of ≥ 5 × 10^12^ vector genomes (v.g.)/mL.

To facilitate *in vivo* CEP162 upregulation, the complete murine CEP162 coding sequence was initially cloned into a pLVX lentiviral backbone driven by a potent constitutive promoter. High-titer lentiviral stocks were generated through transient co-transfection of HEK293T cells with the pLVX-CEP162 construct and standard packaging vectors, followed by supernatant collection at 72 hours, concentration, and purification. Nevertheless, for precise intratesticular administration, we opted for recombinant adeno-associated virus (AAV) delivery. Specifically, AAV serotype 9 (AAV9) vectors encoding CEP162 or a control sequence were microinjected directly into the seminiferous tubules via efferent duct cannulation, delivering a total dose of 2 × 10¹¹ vector genomes (vg) per testis to ensure efficient germ cell transduction. Notably, the AAV construct was deliberately designed without a GFP fusion tag to avoid spectral overlap during subsequent fluorescence-based sperm analyses (e.g., immunofluorescence). Verification of successful CEP162 overexpression in testicular germ cells was achieved 2-4 weeks post-injection through both immunofluorescence staining of testicular sections and Western blot analysis.

For AAV-mediated knockdown of murine Cep162 (accession NM_199316), we designed six distinct pre-miRNA sequences utilizing the BLOCK-iT™ RNAi Designer platform (https://rnaidesigner.thermofisher.com/rnaiexpress/). The open reading frame (ORF) was designated as the target region, with G/C content constrained to 30-70%, and subsequent BLAST searches against the Mus musculus database confirmed target specificity. A scrambled sequence (miR-NC: GTCTCCACGCGCAGTACATTT) served as the negative control. These validated miRNA sequences were subsequently cloned into the pAAV2-CAG-MasterRNAi155-EGFP-WPRE-pA backbone (Taitool Bioscience, Shanghai, China). Concurrently, a partial Cep162 cDNA fragment was integrated into a reporter plasmid (pAAV2-CMV_bGl-mCEP162-mCherry-3×Flag-WPRE-pA) to facilitate efficacy screening. Candidate miRNAs were co-transfected with the reporter construct into HEK293 cells, and their silencing efficiency was assessed by quantifying the attenuation of mCherry fluorescence alongside the reduction of FLAG-CEP162 protein levels via Western blot. The most potent candidate, miR-CEP162 (CACACCTTTACTGACTCTCAT), was ultimately selected for AAV serotype 9 (AAV2/9) packaging alongside the miR-NC control. Taitool Bioscience handled the production, purification, and titration of these recombinant viruses, yielding a final concentration of ≥ 1.1 × 10¹³ vector genomes (v.g.)/mL.

To establish stable overexpression of ASXL2 or DNMT3L in G2 cell lines, their respective coding sequences were individually subcloned into the Ubc-MCS-3×FLAG-SV40-puromycin lentiviral vector, a system that permits simultaneous transgene expression and puromycin selection. These recombinant lentiviral constructs were co-transfected into 293T packaging cells alongside a cocktail of essential packaging plasmids. Over a 48-72 hour period, newly assembled lentiviral particles were secreted into the culture medium. The harvested supernatants were subsequently subjected to ultracentrifugation for concentration and purification, thereby enhancing both viral purity and yield. Functional titers of the resulting viral preparations were quantified by counting fluorescently labeled transduced cells, consistently achieving concentrations of ≥ 1 × 10⁸ transduction units (TU)/mL.

While the physiological oxygen tension within the testis typically ranges from 1% to 2% O_2_^59^, we maintained a 21% O_2_ environment *in vitro* to serve as the normoxic baseline, ensuring compatibility with conventional hypoxia research models^60^-^62^. Conversely, the 1% O_2_ condition was specifically selected to replicate pathological hypoxia scenarios, such as local ischemia induced by varicocele or testicular torsion, which fundamentally differs from the chronic environmental hypoxia (11.7% O_2_) modeled in our *in vivo* murine experiments. Crucially, in diffusion-restricted cell culture systems, active cellular oxygen consumption ensures that the actual pericellular PO_2_ remains lower than the nominal incubator setting. Consequently, our 1% O_2_ incubation protocol likely generates a pericellular PO_2_ that accurately mirrors the severe hypoxic microenvironment associated with pathological male infertility. This methodological choice was substantiated by preliminary trials, which demonstrated robust activation of canonical hypoxia-responsive pathways—including HIF-1α stabilization, DNMT3L suppression, and CEP162 induction—within 36 hours at 1% O_2_, closely mirroring the transcriptional adaptations observed during chronic moderate hypoxia *in vivo*.

To perform *in vivo* genetic rescue of Rab1a signaling, we engineered recombinant AAV9 vectors expressing either wild-type murine Rab1a (accession NM_008996.4) or its constitutively active, GTP-locked mutant (Rab1a-Q70L) 63. The core expression cassette, comprising AAV2 ITRs, a CAG promoter, the Rab1a sequence, and a BGH polyA signal, was integrated into the pAAV-CAG-MCS-BGH polyA backbone (GeneChem, Shanghai, China). Viral packaging was achieved by co-transfecting AAV-293 cells with this construct, pHelper, and the pAAV-RC9 packaging plasmid. The resulting AAV9 particles were purified via iodixanol gradient ultracentrifugation, yielding a titer of 2.5 × 10¹² vg/mL. For the rescue experiments, 8-week-old male mice underwent in situ testicular microinjection via the efferent duct, receiving 10 μL of AAV9-CEP162 either alone or co-administered with AAV9-Rab1a-WT/Q70L (at 1.0 × 10¹² vg/mL). Four weeks post-injection, spermatozoa were harvested from the cauda epididymis, fixed, and analyzed via immunofluorescence with an anti-TEKT2 antibody to evaluate ciliary cargo delivery. Additionally, to evaluate microtubule integrity, isolated sperm flagella underwent Western blotting to quantify polymerized microtubule markers, specifically acetylated α-tubulin (Ac-Tub) and detyrosinated α-tubulin (Glu-Tub).

To functionally rescue flagellar architecture, AAV9-TEKT2 was co-administered with AAV9-CEP162 via intratesticular injection in mice. Subsequent evaluation of sperm progressive motility was conducted utilizing computer-assisted sperm analysis (CASA). Furthermore, ultrastructural examination of the sperm flagella was performed using transmission electron microscopy (TEM). Axonemal integrity was rigorously assessed by calculating the proportion of flagellar cross-sections exhibiting an intact, canonical “9×2+2” microtubule arrangement.

### Prokaryotic expression and purification

To generate an N-terminally His-tagged fusion protein, the 4,212-bp coding region of Cep162 was PCR-amplified using primers designed with NdeI and BamHI restriction sites. Following enzymatic digestion, the target fragment was ligated into the pET-28a(+) expression plasmid. The resulting construct was introduced into chemically competent Escherichia coli BL21(DE3) strains. Recombinant clones were cultured in Luria-Bertani (LB) broth containing 15 µg/mL kanamycin at 37 °C. Once the culture reached an optical density (OD600) of 0.6 to 0.8, protein synthesis was triggered by adding 1 mM isopropyl β-D-1-thiogalactopyranoside (IPTG), followed by a 16-hour incubation at 18 °C to maximize soluble yield. Bacterial pellets were collected via centrifugation (4,000 × g, 20 min, 4^o^C) and subsequently disrupted on ice using a sonicator after resuspension in a specialized lysis solution (50 mM NaH_2_PO_4_, 300 mM NaCl, 10 mM imidazole, pH 8.0) fortified with 1 mg/mL lysozyme and protease inhibitors. The resulting homogenate was cleared by high-speed centrifugation (12,000 × g, 30 min, 4 °C), and the clarified supernatant was mixed with Ni-NTA agarose resin for 1 hour at 4 °C under constant agitation. Following a wash step with 20 mM imidazole to eliminate nonspecific binding, the target protein was eluted using a higher concentration of imidazole (250 mM). The final purified fractions were assessed for purity and molecular weight consistency against theoretical predictions via SDS-PAGE coupled with Coomassie Brilliant Blue staining.

### Probe harmonization and representative probe selection for CEP162 in microarray datasets

For integrative analysis of CEP162 expression across public transcriptomic cohorts, platform-specific probe harmonization was performed prior to gene-level interpretation. For the GSE6969 series, which comprises multiple platform-specific subsets, only one platform was retained for primary analysis to avoid duplicate sample usage and cross-platform heterogeneity. The GPL570 (Affymetrix Human Genome U133 Plus 2.0 Array) subset was excluded because it shared sample identifiers with GSE6872 and therefore did not represent an independent dataset. Among the remaining Illumina-based subsets, GPL2700 (Sentrix HumanRef-8 Expression BeadChip) was selected a priori as the representative platform for GSE6969 owing to its broader transcriptome coverage and more comprehensive gene annotation relative to the earlier GPL2507 Sentrix Human-6 Expression BeadChip. Platform selection was performed independently of the CEP162 differential expression analysis.

For Affymetrix 3' IVT microarrays (e.g., GPL570), representative probe selection followed predefined, direction-independent biological criteria to minimize selection bias. Given the known 3'-end amplification bias inherent to Affymetrix 3' IVT chemistry, probe sets annotated to RefSeq exemplar sequences (protein-coding transcripts with NM_ accessions) were prioritized over those derived from consensus sequences or expressed sequence tag records. Among the probe sets matching the target transcript, the probe set mapping closest to the 3' terminus of the RefSeq reference transcript was designated the representative gene-level probe for visualization and effect-size extraction.

In GSE6872/GPL570, two CEP162 probe sets were identified: 206005_s_at and 206006_s_at. Sequence-level inspection confirmed that both probe sets exactly matched the CEP162 RefSeq transcripts, and NCBI BLASTN analysis against the human RefSeq RNA database did not reveal full-length exact non-CEP162 RefSeq RNA hits for either probe set; therefore, neither was treated as an off-target artifact. Applying the predefined RefSeq annotation and 3'-proximity criteria, 206006_s_at was selected as the representative CEP162 probe. This probe set is annotated to the RefSeq exemplar sequence NM_014895.4 and maps closest to the 3' end of the CEP162 transcript, with the nearest 25-mer probe located 88 nt from the 3' terminus. In contrast, 206005_s_at is annotated to the consensus sequence AK023613 and maps further upstream, with the nearest probe located 690 nt from the 3' terminus (Supplementary Figure 1).

The expression of the representative 3′-proximal probe 206006_s_at was significantly upregulated in infertile samples compared with that in fertile control samples (log_2_FC = 1.825, moderated t = 11.30, P = 2.20 × 10^-10^). The 5'-distal probe 206005_s_at showed significant differential expression in the opposite direction (log_2_FC = -3.416, moderated t = -7.69, P = 1.56 × 10^-7^). This probe-level discordance was retained and reported as a descriptive observation rather than being discarded, acknowledging platform-specific technical variability.

In GSE6969/GPL2700, CEP162 was represented by a single annotated probe (GI_7662445-S); therefore, multiprobe selection was not needed, and this probe was used directly for CEP162 expression analysis (log_2_FC = 0.337, moderated t = 1.48, P = 0.163).

Following probe harmonization, genes were mapped to official HGNC symbols and compared across the five retained datasets: GSE6872, GSE6969 (GPL2700), GSE241326, SRP418387, and SRP418442. To identify robust cross-dataset candidates while accounting for varying statistical power among individual studies, we implemented a directionality-based screening strategy. Specifically, genes exhibiting a positive log_2_-fold change value (log_2_FC > 0) across all five datasets were defined as directionally consistent upregulated candidate genes. This criterion served as a stringent cross-dataset consistency screen rather than requiring nominal significance in every individual cohort. Dataset-specific P values and adjusted P values were retained for comprehensive reporting.

To evaluate the overall statistical evidence for prioritized candidate genes, a cross-dataset effect-size meta-analysis was subsequently performed using the metafor framework in R. Given the methodological differences between the microarray and RNA-seq platforms, a random-effects model using the restricted maximum likelihood (REML) estimator was applied to pool log_2_FC effect sizes and calculate 95% confidence intervals. Between-study heterogeneity was quantified using the I² statistic. For CEP162, a random-effects meta-analysis revealed an overall positive effect across the five retained datasets (pooled log_2_FC = 0.698, 95% CI: 0.018-1.378, P = 0.044), although substantial between-study heterogeneity was detected (I² = 96.2%). Therefore, CEP162 was interpreted as a directionally consistent upregulated candidate with meta-analytic support rather than as a gene showing nominally significant upregulation in every individual dataset.

### Gene expression dataset analysis

Publicly available transcriptomic datasets were retrieved from the Gene Expression Omnibus (GEO) and NCBI BioProject repositories to investigate the expression dynamics of CEP162 and its candidate targets across various male infertility subtypes. Specifically, we curated microarray profiles from two independent teratozoospermia cohorts: GSE6872 and GSE6969 (BioProject PRJNA99311). For the GSE6872 dataset (Affymetrix Human Genome U133 Plus 2.0 Array, GPL570), raw CEL files underwent preprocessing via the robust multiarray average (RMA) algorithm within Bioconductor's affy package, encompassing background adjustment, quantile normalization, and probe-level summarization. Regarding GSE6969, we deliberately selected the GPL2700 platform (Sentrix HumanRef-8 Expression BeadChip) as the primary reference due to its superior transcriptomic coverage and annotation depth compared to alternative Illumina arrays, a decision made independently of any differential expression outcomes. These GPL2700 data were subsequently normalized using variance-stabilizing transformation (VST) coupled with quantile normalization via the lumi package. Additionally, microarray data for nonobstructive azoospermia (NOA) subjects (GSE241326, BioProject PRJNA1007692, Illumina HiSeq 2500) were processed through the same RMA pipeline in Bioconductor.

For asthenozoospermia investigations, we analyzed RNA-sequencing data from two distinct sources: (i) small RNA-seq profiles of seminal plasma exosomes (BioProject PRJNA925687, Illumina HiSeq 2500) and (ii) whole-sperm transcriptomes from patients with varying degrees of idiopathic asthenozoospermia (BioProject PRJNA924578, Illumina HiSeq 2500). Both sequencing datasets were subjected to uniform bioinformatic workflows, comprising adapter trimming, stringent quality control, alignment to the human reference genome, and transcript quantification, ultimately generating normalized abundance metrics (e.g., TPM or FPKM) for subsequent statistical evaluation.

To facilitate robust cross-cohort comparisons, transcript abundance or signal intensity values were evaluated against healthy control baselines. We established a cohort-specific mean expression threshold for each gene. Based on this metric, individual samples were dichotomized into either a “high-expression” cohort (values exceeding the cohort mean) or a “low-expression” cohort (values falling below the mean). This standardized stratification strategy ensured methodological consistency when assessing the transcriptional dysregulation of centrosome- and cilia-associated genes across diverse infertility phenotypes.

### Gene set enrichment analysis

In Supplementary Table** 2**, “Reactome GSEA results for the 24 commonly upregulated genes across five independent datasets,” we retained the following core columns for clarity and reproducibility: ID, description, set size, normalized enrichment score (NES), q value, and core enrichment. These parameters ensure comprehensive reporting of enrichment results while maintaining compatibility with standard GSEA output formats. For downstream interpretation, only pathways meeting both of the following criteria were considered significant: (1) a false discovery rate (FDR)-adjusted q value < 0.15 and (2) a normalized enrichment score (NES) > 1.3. This dual-threshold approach ensures that the reported pathways are not only statistically robust but also exhibit a strong biological effect size.

### Isolation and purity validation of sperm flagellar fractions and head fractions

The sperm flagella and heads were isolated using a modified mechanical shearing and differential centrifugation protocol. Briefly, mature sperm were collected from mouse cauda epididymides and washed in PBS. The cell pellets were resuspended in isolation buffer (100 mM NaCl, 10 mM Tris-HCl (pH 7.4), 1 mM EDTA, 0.5 mM DTT, and protease inhibitor cocktail) and subjected to vortexing (30 s at maximum speed) to shear the flagella from the heads. The suspension was centrifuged at 1,500 × g for 10 min at 4 °C to pellet the sperm heads and intact cells. The supernatant, containing detached flagellar fragments, was then subjected to ultracentrifugation at 100,000 × g for 60 min at 4 °C to pellet the flagella. The resulting head and flagellar pellets were lysed separately in RIPA buffer for downstream analysis.

To confirm the purity of the isolated subcellular fractions, Western blotting was performed using compartment-specific markers. The flagellar fraction was expected to be enriched in α-tubulin, a core axonemal structural protein, and COX IV, a mitochondrial marker localized to the midpiece sheath (which is retained in our flagellar preparations). Conversely, the sperm head fraction was expected to be enriched in SPAM1 (sperm plasma membrane-associated protein 1), a hyaluronidase that digests hyaluronic acid to enable cumulus penetration, and IZUMO1, a sperm head-specific protein essential for sperm-egg fusion. Critically, the flagellar fraction should lack SPAM1 and IZUMO1, and the head fraction should be devoid of α-tubulin and COX IV. The absence of cross-compartment markers confirmed minimal contamination and high purity of the isolated fractions.

### Cellular separation of free tubulin and polymerized tubulin

To isolate distinct tubulin pools, a 1 mL cell suspension was initially sedimented at 6,000 × g for 1 minute at ambient temperature. The resulting cellular pellet was then resuspended in 25 µL of TMMET buffer (comprising 20 mM Tris-HCl [pH 6.8], 140 mM NaCl, 1 mM MgCl_2_, 2 mM EGTA, 0.5% NP-40, and 4 mg/mL paclitaxel) and allowed to incubate for 5 minutes at room temperature to preserve microtubule stability. A subsequent high-speed centrifugation step (14,000 × g for 10 minutes) was employed to partition the mixture. The resulting supernatant, which contained the soluble, unpolymerized tubulin fraction, was carefully separated from the pellet, which was enriched with polymerized microtubule-associated proteins. Aliquots of equal volume from both fractions were then combined with 5× SDS-PAGE loading buffer, heat-denatured, and resolved via SDS-polyacrylamide gel electrophoresis. Following electrophoretic separation, proteins were transferred to a membrane for immunoblotting to evaluate the distribution of tubulin species between the cytosolic and cytoskeletal compartments. Finally, GAPDH, a standard soluble cytoplasmic protein, was utilized as a loading control to validate the purity of the soluble fraction.

### *In vitro* microtubule depolymerization assay

To assess the direct effect of TEKT2 on microtubule stability, a temperature-induced depolymerization assay was performed using purified porcine brain tubulin (2 mg/mL). Microtubules were polymerized in BRB80 buffer supplemented with 1 mM GTP at 37 °C for 30 min and subsequently stabilized with 20 µM paclitaxel. The stabilized microtubules were then diluted to 1 mg/mL and incubated with either 100 nM purified recombinant TEKT2 protein or vehicle control buffer for 15 minutes at 37 °C.

Depolymerization was initiated by rapidly diluting the reaction mixture 1:10 into prechilled BRB80 buffer (4 °C). Microtubule stability was quantified by monitoring the decrease in solution turbidity at 350 nm (OD_350_) over 30 minutes using a temperature-controlled spectrophotometer. The depolymerization kinetics were analyzed by comparing the half-time (t_1/2_) and final OD_350_ plateau between treatment groups, where a rightward shift in the curve and greater final absorbance value indicated TEKT2-mediated stabilization. All experiments were independently repeated at least three times.

### Microtubule regrowth assay

To evaluate microtubule nucleation capacity, cells were seeded onto sterile 8-mm glass coverslips (Thermo Fisher Scientific) housed within 3.5-cm culture dishes. Prior to any pharmacological intervention, a baseline coverslip was fixed to verify initial cellular morphology. To prevent the detachment of mitotic cells during subsequent steps, coverslips were carefully moved to fresh dishes containing the appropriate treatment media. Microtubule networks were completely dismantled by exposing the cells to 10 µM nocodazole in DMEM/F12 medium, enriched with either 10% fetal bovine serum (FBS) or a mixture of 5% horse serum (HS) and 2.5% FBS, for a duration of 1 hour at 37 °C.

After thoroughly washing away the nocodazole, the coverslips underwent a sequential recovery phase: they were first immersed in DMEM with 10% FBS for 5 minutes, or alternatively in DMEM with 5% HS plus 2.5% FBS for 10 minutes. Subsequently, the cells were returned to their standard complete culture medium and maintained at 37 °C for the specified recovery intervals.

At each recovery time point, cells expressing GFP-tagged α-tubulin (from the pEGFP-Tubulin plasmid, custom made by GeneChem Co., Ltd., Shanghai, China, encoding α-tubulin fused to EGFP) were fixed and costained for pericentrin (a centrosome marker) and α-tubulin (to label microtubules). A cell was scored as “regrowth positive” only if it displayed (i) a discrete, focused pericentrin punctum and (ii) a radial array of α-tubulin microtubules emanating from this focus, indicating functional microtubule nucleation from the centrosome. The percentage of such cells was quantified from at least 200 cells per condition across multiple biological replicates and reported as a measure of microtubule regrowth capacity, not centrosome structural integrity. Importantly, this assay measures microtubule nucleation and polymerization dynamics following depolymerization. To assess microtubule stability, we performed Western blotting for posttranslationally modified tubulin (acetylated and detyrosinated) and cold depolymerization assays.

### Cell viability assay

To evaluate cellular viability, a WST-8 colorimetric assay (Cell Counting Kit-8, Beyotime) was employed. Briefly, cells were plated into 96-well microplates at a density of 2 × 10⁴ cells per well and subsequently subjected to hypoxic stress. At the designated time points, the culture medium was supplemented with 10 μL of the WST-8 solution, and the plates were incubated for an additional 60 minutes prior to the termination of the experiment. The optical density of the generated formazan dye was then recorded at a wavelength of 450 nm utilizing a Multiskan GO microplate spectrophotometer (Thermo Scientific, MA, USA). The relative cell survival rate was ultimately calculated by normalizing the absorbance values of the hypoxia-treated groups against those of the untreated normoxic counterparts.

### Assessment of Sperm Kinematics via sperm class analysis (SCA) system

All experimental procedures involving animals received ethical approval from the Institutional Animal Care and Use Committee of Army Medical University, adhering to the guidelines set forth by the National Institutes of Health (NIH Publication No. 8023, revised 1978). The murine models were kept in a controlled vivarium environment featuring a 12-hour light/dark photoperiod, a stable ambient temperature ranging from 22 to 24^o^C, and humidity levels between 40% and 60%, with unrestricted access to standard chow and water. To establish the hypoxia model, male mice (starting at 10 weeks of age) were continuously housed in a hypobaric chamber that simulated a high-altitude environment of 5,800 meters, whereas the normoxic control counterparts were kept at a basal altitude of 300 meters.

To evaluate sperm quality, various kinematic metrics—specifically curvilinear velocity (VCL), straight-line velocity (VSL), average path velocity (VAP), straightness (STR), beat-cross frequency (BCF), and amplitude of lateral head displacement (ALH)—along with sperm concentration and motility, were recorded utilizing a computer-assisted sperm analysis (SCA) platform (MICROPTIC S.L., Barcelona, Spain). Prior to sample collection, the mice were deeply anesthetized through the inhalation of 4% isoflurane in pure oxygen for induction, and subsequently maintained at a 1.5-2% isoflurane concentration via a facial mask connected to a Harvard Apparatus Model 722 vaporizer. The adequacy of anesthesia was continuously verified by monitoring the pedal reflex and ensuring a respiratory rate of 60-80 breaths per minute, followed by euthanasia via anesthetic overdose. Following euthanasia, the epididymides were rapidly excised, weighed, and anatomically divided into the head, body, and cauda segments. Spermatozoa were liberated from the caudal epididymis by mincing the tissue in F12 medium supplemented with 0.5% BSA, which had been prewarmed to 37 °C, and allowing it to liquefy for 10 minutes. Subsequently, a 50-µL portion of the resulting sperm suspension was thoroughly mixed after a 1:50 dilution with PBS (pH 7.2), and the prepared sample was loaded into the SCA system for quantitative analysis.

### Assessment of epididymal sperm morphology

To evaluate the structural integrity of the epididymal spermatozoa, transmission scanning electron microscopy (TSEM) was employed. Initially, the specimens underwent primary fixation in a solution of 2.5% glutaraldehyde prepared in 0.1 M cacodylate buffer (pH 7.4; osmolarity ~320 mOsm/kg). This was followed by a secondary fixation step utilizing a mixture of 1% osmium tetroxide and 1.5% potassium ferrocyanide. Subsequently, the samples were dehydrated and dried via the critical point method employing liquid CO₂ in a Leica EM CPD300 apparatus (operating at a 31.1 °C critical point with a triple-purge protocol). Finally, morphological visualization was carried out utilizing a ZEISS CROSSBEAM 340 microscope fitted with a STEM3 detector. The imaging parameters were set to a 30 µm aperture, 5 kV acceleration voltage, and 50 pA probe current, with the system's calibration routinely validated using NIST SRM 2066 standards.

### Transmission electron microscopy and axonemal integrity scoring

The sperm samples were fixed in 2.5% glutaraldehyde in 0.1 M cacodylate buffer (pH 7.4), followed by postfixation with 1% osmium tetroxide. The samples were dehydrated through a graded ethanol series, embedded in epoxy resin, and sectioned into ultrathin (~70 nm) slices using an ultramicrotome. Sections were collected on copper grids, stained with uranyl acetate and lead citrate, and examined using a transmission electron microscope (e.g., Tecnai G2 Spirit, Thermo Fisher Scientific) at 80-120 kV. The integrity of the axoneme was assessed by counting the percentage of flagellar cross-sections that displayed a complete “9×2+2” microtubule arrangement (i.e., nine outer DMTs and two central singlet microtubules) under a transmission electron microscope. Sections with missing, fragmented, or disorganized outer doublets were scored as defective.

### Adhesion force measurements by AFM

To evaluate the surface mechanics of the sperm flagella, we utilized an Asylum MFP-3D atomic force microscope integrated with an Olympus IX51 inverted optical microscope for precise visual targeting. Force-distance mapping was conducted at scanning frequencies ranging from 0.3 to 2.0 Hz. The measurements employed silicon nitride (Si3N4) or silicon (Si) cantilevers attached with spherical probes (Novascan), specifically 10-µm glass, 10-µm gold-coated glass, and 12.5-µm polystyrene beads, possessing spring constants between 0.6 and 14 N/m. From the resulting force curves, two primary mechanical parameters were extracted: the sample modulus (Esample), which quantifies the intrinsic stiffness and deformation resistance of the flagellar material, and the reduced modulus (E0), which characterizes the overall elastic behavior of the probe-sample contact interface. Elevated E0 values correspond to increased interfacial rigidity and decreased deformability.

### Papanicolaou staining

Initially, air-dried sperm specimens underwent fixation in 95% ethanol for a duration of 15 minutes. This was followed by a descending hydration gradient, where the slides were sequentially immersed in 75% and 50% ethanol, and finally distilled water, with each step lasting 30 seconds. For nuclear visualization, the samples were treated with Harris hematoxylin for 5 minutes, briefly washed, and then differentiated using a 1% acid-alcohol solution for a few seconds. A subsequent 10-minute wash under running tap water facilitated the bluing process. Following nuclear staining, the slides were dehydrated by passing through 50%, 75%, and 95% ethanol for 30 seconds, 30 seconds, and 2 minutes, respectively. Cytoplasmic counterstaining was achieved by applying orange G for 1 minute, followed by two brief 15-second rinses in 95% ethanol. The samples were then exposed to EA36 (a mixture of light green and eosin) for 5 minutes. The staining procedure was concluded with a final clearing step involving two 15-second dips in 95% ethanol and two 45-second incubations in absolute ethanol, rendering the slides ready for microscopic mounting.

### Isolation of round and elongating spermatids

To isolate specific spermatogenic populations, testes were harvested from adult (8-week-old) male mice. Following decapsulation, the testicular tissues underwent a two-step enzymatic dissociation. Initially, the tissues were incubated in DMEM:F12 or PBS containing 25 μg/mL collagenase IV and 400 U/mL DNase I at 35^o^C for 5 minutes. Subsequently, 200 μg/mL trypsin and additional DNase I were introduced for a 4-minute agitation period at 35^o^C, after which fetal bovine serum (FBS) was added to halt enzymatic activity. The resulting single-cell suspensions were filtered through a 40-μm strainer, rinsed with FACS buffer, and subjected to red blood cell lysis using a commercial buffer (Beyotime). For fluorescence-activated cell sorting (FACS), the cells were labeled with Hoechst 33342 and dye780 (Invitrogen) for 25 minutes, utilizing 15 μL of Hoechst and 2 μL of dye780 per 4×10⁷ cells. Sorting was executed on a BD FACSAria instrument. Hoechst fluorescence was excited by a 375-nm laser, and emissions were captured in the “Ho Blue” (450/40 nm) and “Ho Red” (670 nm longpass) channels—the latter also capturing the dye780 signal via a 610-nm longpass dichroic mirror. Forward and side scatter profiles were recorded using a 488-nm laser. Based on their distinct DNA content signatures, round spermatids (1n haploid) and elongating spermatids were discriminated and sorted through a 70-μm nozzle at a rate of 2,000 to 3,500 events per second. Following the acquisition of at least 500,000 events for precise gating, the purified cells were deposited into polypropylene tubes pre-filled with 1 mL of Gey's balanced salt solution (GBSS) containing 5% FBS. Finally, cell viability and density were evaluated via trypan blue exclusion assays.

### Immunofluorescence staining

Cells cultured on glass coverslips underwent fixation using 4% paraformaldehyde and permeabilization with 0.1% Triton X-100, followed by three washes using the provided immunofluorescence washing buffer (Beyotime). To prevent non-specific background, the samples were treated with a custom blocking buffer comprising 5% goat serum, 1% BSA, and 0.1% Tween-20 for 1 hour at ambient temperature. For the dual-channel staining protocol, coverslips were first exposed to a primary rabbit polyclonal antibody targeting CEP162 (1:200, HPA030170, Sigma-Aldrich) for 1 hour. After washing, the samples were probed with an Alexa Fluor™ 488-labeled goat anti-rabbit secondary antibody (1:500, A-11008, Thermo Fisher) for another hour. Next, the specimens were incubated with either a mouse monoclonal anti-CEP250 antibody (1:200, 66814-1, Proteintech) or an anti-CP110 antibody (1:200, 66448-1-Ig, Proteintech) for 1 hour, which was then followed by an Alexa Fluor™ 555-conjugated goat anti-mouse secondary antibody (1:500, A-21422, Thermo Fisher) for 1 hour. Finally, nuclear counterstaining was performed using DAPI (C1005, Beyotime) for 5 minutes. The coverslips were then sealed with mounting medium, and fluorescence imaging was conducted using an Olympus IX81 laser scanning confocal microscope at a 400× magnification in randomly chosen visual fields.

### Targeted quantification of intracellular succinate by LC‒MS/MS

To modulate intracellular succinate levels, G2 cells were treated with 100 μM dimethyl fumarate (DMF; Sigma‒Aldrich, Cat# 242926), a cell-permeable prodrug of fumarate, to increase succinate oxidation via the mass action-driven promotion of succinate dehydrogenase forward flux, specifically the conversion of succinate to fumarate. DMF was dissolved in DMSO (stock: 100 mM) and diluted to the working concentration in culture medium immediately before use, with a final DMSO concentration ≤ 0.1% (v/v). Following treatment, the G2 cells were rapidly harvested and washed with ice-cold PBS, after which the metabolites were extracted using 80% methanol containing a stable isotope-labeled internal standard (succinate-d4). After centrifugation and drying under vacuum, the samples were reconstituted and analyzed on a Q Exactive HF-X mass spectrometer coupled to a Vanquish UHPLC system. Succinate was monitored in negative ionization mode using multiple reaction monitoring (MRM: m/z 117.0 transitioning to 73.0). Quantification was performed against a calibration curve constructed from authentic standards, and the data were normalized to the total protein content determined by a BCA assay.

### Dual-channel detection of CEP162 pre-mRNA and protein in hypoxic G2 cells by sequential immunofluorescence and smFISH

Cells were fixed with 4% PFA, permeabilized (0.5% Triton X-100), and blocked (5% goat serum, 1% BSA). The CEP162 protein was labeled with a rabbit anti-CEP162 antibody (1:200) and Alexa Fluor 555-conjugated goat anti-rabbit IgG (1:500, 561 nm excitation). After postfixation, smFISH was performed using the following CAL Fluor 488-labeled probes for CEP162 (NM_199316.2): intron-specific (5'-CGTGTTATGGCAAAGA TGAGAAAGACAAAGGATGA-3') and exon-specific (5'-TCAGAAACAACCAATTCATTAGCTTC TAAGCTGTCA-3'). Hybridization was carried out in 10% formamide, 2× SSC, and 10% dextran sulfate at 37 °C overnight. Washes were performed with 10% formamide/2× SSC (37 °C), 2× SSC (RT), and 1× SSC (RT). Nuclei were counterstained with DAPI, and ProLong Diamond was used for mounting. Images were acquired with an Olympus FV3000 confocal microscope (405, 488, and 561 nm laser irradiation).

### Actinomycin D chase assay to assess CEP162 mRNA stability

To determine whether hypoxia-induced changes in CEP162 mRNA abundance or its suppression by DNMT3L/ASXL2 overexpression result from altered transcription or posttranscriptional stability, we performed an actinomycin D chase assay. G2 cells transfected with control, DNMT3L-3×Flag, or ASXL2-3×Flag constructs were exposed to 1% O_2_ for 48 h and subsequently treated with ActD (5 μg/mL) to block new transcription and harvested at 0, 1, 2, 4, and 6 h. Total RNA was extracted with TRIzol, reverse transcribed, and quantified by qRT‒PCR using SYBR Green and primers specific to mature *CEP162* mRNA (forward: 5'-TCCTTATGGACAAAGCAGTGGTG-3' and reverse: 5'-CAAATCGGACTCGGTGGTAGAG-3'), with β-actin (forward: 5'-AGAGGGAAATCGTGCGTGAC-3' and reverse: 5'-CAATAGTGATGACCTGGCCGT-3') as a stable reference. CEP162 mRNA levels were normalized to that at t = 0 (100%) and fitted to the following exponential decay model to calculate the half-life (t_1/2_): remaining mRNA (%) = 100 × e^^{-kt}^.

### RNA extraction and real-time polymerase chain reaction

Cellular total RNA was purified utilizing TRIzol reagent (Sangon) following the manufacturer's protocol. Subsequently, 1 μg of the purified RNA served as the template for reverse transcription to synthesize cDNA, employing a TaKaRa RNA PCR kit (TaKaRa Bio). The quantitative real-time PCR (qRT-PCR) assays were executed in 96-well plates utilizing an MJ Research Light Cycler system. The reaction mixture contained SYBR Green (Bio-Rad) as the fluorescent dye and 1 μg of the synthesized cDNA. Target gene amplification was achieved using specific primer pairs, notably for *CEP162* (Forward: 5'-TCCTTATGGACAAAGCAGTGGTG-3'; Reverse: 5'-CAAATCGGACTCGGTGGTAGAG-3'). Relative transcript abundances were determined through standard curve quantification and subsequently normalized to the endogenous reference gene β-actin to correct for any discrepancies in initial RNA loading.

### Western blotting

Cellular proteins were harvested utilizing a Western and IP lysis buffer (Beyotime) fortified with 1 mM PMSF, while the separation of nuclear and cytosolic compartments was achieved via a specific fractionation kit (Beyotime) per the vendor's guidelines. Protein samples were resolved on 10% SDS-PAGE gels and subsequently electroblotted onto PVDF membranes (Beyotime). Following a blocking step, the membranes were probed overnight at 4 °C with the following primary antibodies: anti-TEKT1 (1:1,000; ab182777; Abcam), anti-TEKT2 (1:1,000; ab213369; Abcam), anti-ARHGEF18 (1:1,000; ab96520; Abcam), anti-CEP162 (1:1,000; HPA030170; Sigma‒Aldrich), and anti-β-actin (1:1,000; AA128; Beyotime). After extensive washing in TBST, the blots were exposed to HRP-tagged secondary antibodies (SA00001-2-100ul and SA00001-1-100ul; Beyotime). Target protein bands were ultimately visualized employing the NBT/BCIP chromogenic detection system (Beyotime).

### Coimmunoprecipitation

For co-immunoprecipitation (Co-IP) assays, cellular lysis was performed on ice for 1 hour utilizing a specialized buffer (20 mM Tris-HCl pH 7.5, 150 mM NaCl, 1% NP-40, 10% glycerol, 1 mM EDTA) fortified with a protease inhibitor cocktail (Beyotime, Shanghai, China). Following centrifugation, the clarified supernatants were harvested. To circumvent the interference of antibody heavy and light chains during subsequent Western blotting, the primary antibody intended for IP was covalently conjugated to Protein G agarose beads via disuccinimidyl suberate (DSS; Thermo Fisher Scientific, Cat# 21555) for 30 minutes at room temperature, and the reaction was subsequently quenched with 1 M Tris-HCl (pH 7.5). These functionalized beads were then incubated with the prepared cell lysates overnight at 4^o^C under gentle agitation. The following day, the bound immune complexes were isolated and subjected to four rigorous washes with ice-cold washing buffer (20 mM Tris-HCl pH 7.5, 150 mM NaCl, 0.1% NP-40, 10% glycerol, 1 mM EDTA, pre-chilled to 4.0 ± 0.5^o^C) prior to Western blot evaluation.

### Microtubule cosedimentation assay

To examine CEP162-microtubule interactions, 1 μM recombinant His-CEP162 (purified from *E. coli* BL21(DE3) via Ni-NTA) was incubated with 1 mg/mL polymerized porcine brain tubulin (2 mg/mL in PEM buffer: 80 mM PIPES pH 6.9, 2 mM MgCl_2_, 0.5 mM EGTA, 1 mM GTP, and 20 μM paclitaxel) for 30 min at 37°C under normoxic (21% O_2_) or hypoxic (1% O_2_) conditions, with hypoxic buffers preequilibrated for 2 h in a hypoxia workstation. Mixtures were ultracentrifuged at 100,000 × g for 30 min at 37 °C in a prewarmed rotor; the supernatant (unbound) and pellet (microtubule-bound) fractions were analyzed by 10% SDS‒PAGE and Western blotting using anti-CEP162 (1:1,000; HPA030170) and anti-α-tubulin (1:1,000; AF2827; Beyotime) antibodies. Binding was quantified as follows: % bound = [pellet/(pellet + supernatant)] × 100. All experiments were performed in triplicate, with hypoxia assays conducted entirely within the hypoxia workstation to maintain oxygen tension.

### [³H]GDP release assay

Nucleotide exchange reactions were performed in 100 μL of GEF buffer (50 mM Tris-HCl (pH 7.5), 100 mM NaCl, 1 mM DTT, 1 mM EDTA, 2 mM MgCl_2_, and 50 μg/mL BSA) containing 4 pmol of [³H]GDP-preloaded Rab1a-His and the indicated immunoprecipitates (control IgG, purified CEP162, TRAPPC9 IP, or CEP162 + TRAPPC9 IP). Reactions were initiated by the addition of 10 mM MgCl_2_ and incubated at 37 °C. At the indicated time points (0, 5, 10, 20, and 30 min), the reactions were terminated by the addition of 2 mL of ice-cold stop buffer (25 mM Tris-HCl, pH 8.0, 20 mM MgCl_2_). The samples were immediately filtered through 0.4 μm nitrocellulose membranes (Millipore) under vacuum and then washed three times with 5 mL of stop buffer. The membrane-bound radioactivity (representing remaining [³H]GDP-Rab1a) was quantified by liquid scintillation counting (PerkinElmer Tri-Carb). The amount of [³H]GDP released was calculated as the difference between the initial bound radioactivity and residual radioactivity at each time point.

### [³⁵S]GTPγS binding assay

Additionally, GTPγS binding assays were performed to directly measure GTP loading. Reactions (100 μL) contained 4 pmol of nucleotide-free Rab1a-His (prepared by EDTA treatment and Mg^2+^ reconstitution), 50 pmol of [³⁵S]GTPγS (PerkinElmer, ~10⁴ cpm/pmol), and the indicated immunoprecipitates in GEF buffer. The reaction systems were incubated at 37 °C for the indicated durations and then stopped and processed as described for the GDP release assay. Membrane-bound radioactivity (representing [³⁵S]GTPγS-Rab1a) was quantified by scintillation counting. Data are presented as the amount of [³⁵S]GTPγS incorporated into Rab1a over time.

### dCas9-mediated in situ chromatin capture coupled with 4D Label-Free proteomics

To elucidate the oxygen-sensitive transcriptional regulators governing CEP162 expression during spermiogenesis, we performed dCas9-based chromatin affinity purification (CAPTURE) in both round and elongating spermatids, in which stable coexpression of FB-dCas9 and BirA-V5 enabled locus-specific biotinylation upon lentiviral delivery of sgRNAs targeting the CEP162 promoter. Following formaldehyde crosslinking and sonication, chromatin-bound protein complexes were isolated from the targeted genomic regions via streptavidin bead-based affinity purification from both cell types under normoxic and hypoxic conditions, and these purified complexes were subjected to 4D label-free quantitative proteomics (LC‒MS/MS with ion mobility separation) to comprehensively identify and quantify the dynamic composition of epigenetic regulatory complexes.

### CUT&Tag-seq

The CUT&Tag sequencing was executed on primary round spermatids utilizing the CUT&Tag 4.0 High-Sensitivity Kit (N259-YH01, Novoprotein, Suzhou, China), adhering to established protocols^64^. In brief, thawed formaldehyde-crosslinked cells had their nuclei captured by concanavalin A-functionalized magnetic beads. Post-blocking, these bead-bound nuclei underwent sequential incubation with primary (1-2 hours) and secondary (0.5-1 hour) antibodies in a wash buffer comprising 20 mM HEPES (pH 7.5), 150 mM NaCl, 0.5 mM spermidine, and EDTA-free protease inhibitors at ambient temperature. Subsequent to washing, the beads were resuspended in high-salt wash buffer (300 mM NaCl) containing 12.5 nM pA(G)-Tn5 for 1 hour. Tagmentation was then induced by adding 10 mM MgCl_2_ and incubating for 1 hour at 37 °C. The enzymatic reaction was halted using a termination buffer alongside proteinase K, followed by a 2-hour incubation at 55 °C to reverse crosslinks and degrade proteins. Finally, the liberated DNA was purified and amplified for library construction. Comprehensive quality control metrics, including raw and clean data sizes alongside mitochondrial contamination rates, are detailed in **Supplementary Table 6**.

Raw sequencing data in FASTQ format, generated via Illumina paired-end sequencing, underwent rigorous quality control. Adapter sequences and low-quality bases were trimmed utilizing Cutadapt (v1.16) with specific parameters (--discard-trimmed -n 3 -e 0.1 -a AGATCGGAAGAGC -A) to yield high-quality clean reads. Subsequent alignment of these processed reads against the murine reference genome (GRCm38) was executed via Bowtie2 (v2.4.1) under default parameters, filtering for exclusively unique mappings. To detect significant enrichment regions, peak calling was conducted employing HOMER (v4.11.1), applying stringent criteria of a p-value < 0.0001 and a minimum fold enrichment of 4. Finally, the genomic locations of the nearest genes to each peak were annotated, and intersection analyses to visualize shared and distinct binding sites across experimental groups were carried out utilizing the VennDiagram package (v1.6.20) in the R environment.

### ChIP‒qPCR and Sequential ChIP‒qPCR

To assess protein-DNA interactions, chromatin immunoprecipitation assays were conducted following standard procedures. In short, cleared chromatin extracts were probed with specific primary antibodies against ASXL2 (PA5-70292; Thermo Fisher Scientific) or DNMT3L (ab251178; Abcam), while normal rabbit IgG (ab172730; Abcam) served as the background control. To perform sequential ChIP (re-ChIP) experiments, the initial immune complexes were released from the beads via a 30-minute incubation with 10 mM dithiothreitol (DTT) at 37 °C under mild agitation. The eluted material was then subjected to a secondary immunoprecipitation step utilizing a different antibody. Subsequent to DNA purification, quantitative real-time PCR (qPCR) was employed to measure the abundance of the target sequences alongside the corresponding input samples. Target locus occupancy was expressed as a percentage of the total input DNA (% input). For the calculation of fold enrichment, the specific immunoprecipitation signals were standardized against the background IgG signals. The specific primer pair utilized for amplifying the CEP162 promoter was as follows: forward, 5'-TGGCACCTGTTAGCTCAATG-3'; reverse, 5'-TCTCCCTCTCCGTGTATGTG-3'.

### Methylated DNA Immunoprecipitation followed by quantitative real-time PCR

Genomic DNA was sheared by sonication to an average fragment size of 200-1000 bp, and 5.5 μg of fragmented DNA was denatured at 95 °C for 10 min, followed by immediate cooling on ice for 5 min. The DNA was then immunoprecipitated overnight at 4 °C with 2.5 µl of a monoclonal anti-5-methylcytosine antibody (clone MC-57, Diagenode) in IP buffer (50 mM NaH2PO4 (pH 7.0), 0.7 M NaCl, and 0.4 mM Triton X-100). The antibody-bound DNA was captured using Protein A/G PLUS-Agarose beads, washed multiple times, and eluted after digestion with proteinase K. The methylated DNA was purified by phenol‒chloroform extraction and ethanol precipitation. Input DNA (nonimmunoprecipitated) was saved prior to immunoprecipitation for normalization. Quantitative real-time PCR was performed on both MeDIP-enriched and input DNA using gene-specific primers designed to target CpG-rich regions near transcription start sites or within exons. The relative enrichment of methylated DNA was quantified as the fold enrichment using the following formula: fold enrichment = 2^(Ct[Input] - Ct[MeDIP]), with normalization to the input DNA and negative control regions.

### Electrophoretic mobility shift assays (EMSAs)

To investigate the direct binding of DNMT3L to the *Cep162* promoter, a specific DNA fragment (rn6; chr8:94,920,191-94,920,220) was PCR-amplified and subsequently labeled at the 3' end with biotin utilizing the Biotin 3' End Labeling Kit (GS008, Beyotime Biotechnology) according to the vendor's guidelines. This probe sequence (5'-CTCAATGCCATCTTCTCACCCAGTCTCTCC-3') encompasses a conserved regulatory element situated 222 to 251 base pairs downstream of the rat *Cep162* transcriptional start site (NM_001277060.1). For the binding reaction, 10 nM of the biotinylated oligonucleotide was incubated with 10 μg of purified recombinant DNMT3L protein in the supplied binding buffer (GS009, Beyotime Biotechnology) for 20 minutes at ambient temperature. The resulting protein-DNA complexes were separated via electrophoresis on a 15% native polyacrylamide gel (1 mm thickness) and subsequently blotted onto Hybond™-N+ nylon membranes (FFN13, Beyotime Biotechnology). Following UV-mediated crosslinking to immobilize the DNA, the shifted bands were visualized employing the LightShift Chemiluminescent EMSA detection system (GS009, Beyotime Biotechnology).

### Statistical analysis

To process the CUT&Tag peak profiles, we applied normalization procedures to adjust for sequencing depth and technical biases, subsequently applying a logarithmic transformation to stabilize variance and approximate a normal distribution. Flow cytometry data were subjected to background correction and normalization using z score transformation where applicable. Throughout the manuscript, all quantitative measurements are expressed as mean ± standard deviation (SD). Comprehensive statistical details, including exact sample sizes (n) and the designation of biological versus technical replicates, are specified within the respective figure legends to ensure transparency. To evaluate statistical differences, we employed two-tailed unpaired Student's t-tests for two-group comparisons, while one-way or two-way repeated-measures ANOVAs coupled with Tukey's multiple comparison tests were utilized for multi-group or repeated-measure analyses. Statistical significance thresholds, adjusted for family-wise error rates, are denoted as follows: ns, not significant; *P < 0.05; **P < 0.01; ***P < 0.001; and ****P < 0.0001. All statistical computations and graphical representations were executed using GraphPad Prism software (version 9.0).

## Supplementary Material

Supplementary figures and tables.

## Figures and Tables

**Figure 1:**
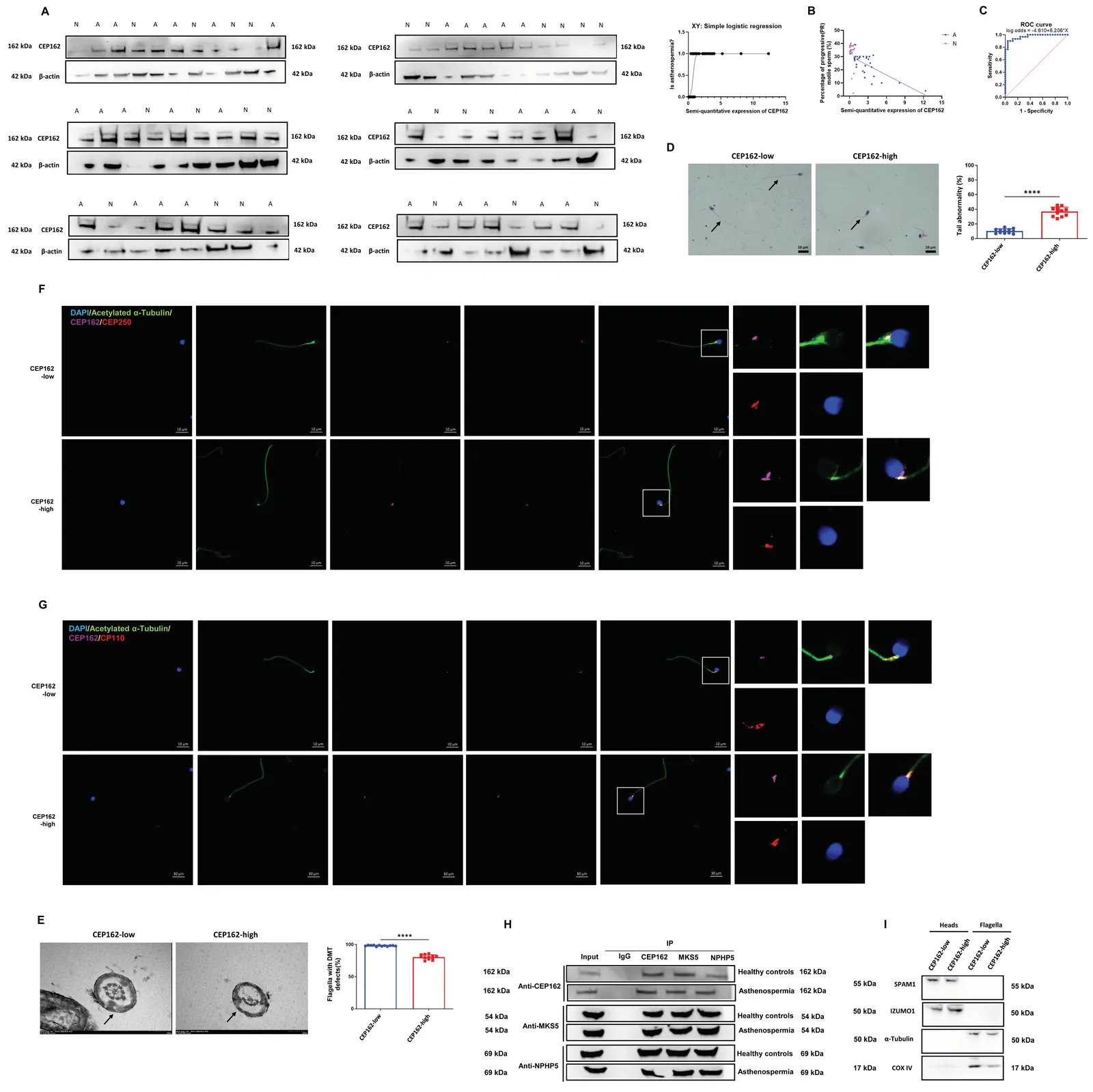
** CEP162 expression in patients with clinical asthenozoospermia is elevated. (A)** Immunoblot showing increased CEP162 expression in sperm from men with asthenozoospermia (A) compared with that in normozoospermic (N) controls. The samples are labeled accordingly (N = normozoospermia, A = asthenozoospermia). Simple logistic regression analysis demonstrating the association between semiquantitative CEP162 expression levels and the likelihood of asthenozoospermia. **(B)** Scatter plot showing the correlation between semiquantitative CEP162 expression and the percentage of progressive motile sperm (PR) in human sperm samples. Each point represents an individual patient (n = 55) color- and shape-coded by diagnosis according to the 2021 WHO criteria: purple triangles, normozoospermic controls (N, n = 25); blue circles, asthenozoospermic patients (A, n = 30). The regression line is shown in black. **(C)** ROC curve analysis demonstrating the diagnostic performance of CEP162 expression in distinguishing asthenozoospermic patients from healthy controls. The area under the curve (AUC) indicates high accuracy, with the logistic regression model showing strong predictive power for identifying asthenozoospermia patients on the basis of CEP162 levels. **(D)** Representative Papanicolaou-stained micrographs of human sperm from normozoospermic (CEP162-low) and asthenozoospermic (CEP162-high) individuals. In the asthenozoospermic/CEP162-high panel, the arrow indicates a sperm with a bent or angulated flagellum, a common morphological defect observed in this group. In the normozoospermic/CEP162-low panel, the arrow highlights a representative sperm with normal flagellar morphology for comparison. Scale bar = 10 μm. Quantitative comparison of flagellar malformation rates between the CEP162-high (n = 28) and CEP162-low (n = 27) groups. The tail abnormalities scored included bent, coiled, short, and absent flagella. **(E)** Transmission electron micrographs of sperm flagellar cross-sections, with defect rates of axonemal doublet microtubules (DMTs) quantified from ≥200 cross-sections per group by a blinded observer and stratified post hoc into CEP162-high (n = 28) and CEP162-low (n = 27) groups on the basis of CEP162 expression. Dual immunofluorescence staining revealed the colocalization of CEP162 with the proximal centriolar marker CEP250 **(F)** and the distal centriolar marker CP110 **(G)** in normozoospermic control sperm; acetylated α-tubulin was used to label the axoneme, and DAPI was used to stain the nuclei. **(H)** Coimmunoprecipitation of human sperm lysates revealed that CEP162 associates with MKS5 and NPHP5; IgG served as a negative control. **(I)** WB of compartment-specific markers in CEP162-low vs. CEP162-high head fractions (isolated by density gradient centrifugation). Head markers: SPAM1 and IZUMO1; α-tubulin and COX IV are flagellar markers. All the data are presented as the means ± SDs. In (A-C), n = 55 human donors (25 normozoospermic controls, 30 asthenozoospermic patients); normalization was not performed; correlation: Pearson; diagnostic model: logistic regression; significance: *P* = 0.0302. In (D-E), flagellar defect rates were determined from ≥ 200 sperm per group across the CEP162-high (n = 28) and CEP162-low (n = 27) cohorts; unpaired two-tailed Student's t test; ****P < 0.0001. (F-G) show representative images derived from 3 independent experimental replicates per donor, with n = 5 normozoospermic donors. Localization was assessed by visual inspection of a total of ≥50 sperm from all the donors combined. No formal statistical analyses were performed for these qualitative imaging assays. (H-I) show representative images from a single independent experiment. No formal statistical testing was applied to this single replicate.

**Figure 2:**
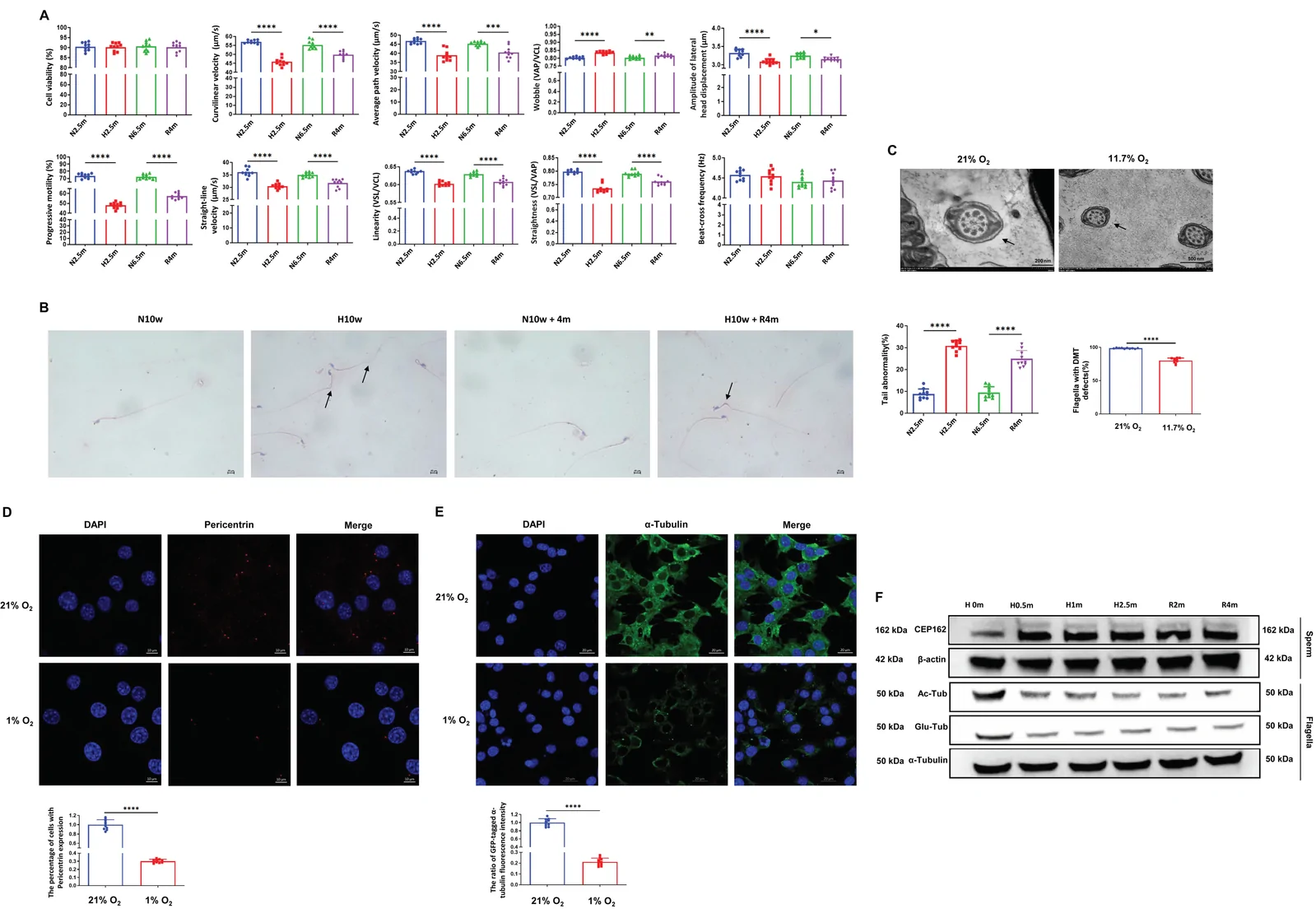
** Defects in hypoxia-induced sperm motility are correlated with CEP162 overexpression in a murine model. (A)** Sperm viability and motility parameters after 10 weeks of exposure to 11.7% O_2_ followed by 4 months of reoxygenation, as assessed by computer-assisted sperm analysis (CASA; Microptic S.L., Barcelona, Spain). Group labels indicate treatment conditions and duration: N = normoxia (21% O_2_), H = hypoxia (11.7% O_2_), R = reoxygenation; numbers denote months (e.g., H2.5m = 2.5 months of hypoxia). The parameters included progressive motility, curvilinear velocity (VCL), straight-line velocity (VSL), average path velocity (VAP), straightness (STR), beat-cross frequency (BCF), amplitude of lateral head displacement (ALH), linearity (LIN), and wobble (WOB). **(B)** Assessment of sperm tail abnormalities by Papanicolaou staining in mice exposed to 10 weeks of hypoxia (11.7% O_2_) or hypoxia followed by 4 months of reoxygenation. The arrow indicates a sperm with a bent or angulated flagellum. Quantitative comparison shows the percentage of sperm exhibiting structural flagellar defects, including bent, coiled, short, or absent flagella. **(C)** Ultrastructural analysis of the sperm flagellar axonemal DMT defects by transmission electron microscopy (TEM). Scale bars: 200 nm (left panel) and 500 nm (right panel). **(D)** Representative immunofluorescence images showing impaired microtubule regrowth in G2 cells following nocodazole washout under 1% O_2_. The percentage of G2 cells with focused pericentrin foci surrounded by radial α-tubulin asters was used as a functional readout of centrosome-mediated microtubule nucleation efficiency. **(E)** Quantification of microtubule aster size and GFP-α-tubulin fluorescence intensity in G2 cells, reflecting the kinetics of microtubule polymerization following nocodazole washout. **(F)** Western blot analysis of CEP162, acetylated α-tubulin (Ac-Tub), and detyrosinated α-tubulin (Glu-Tub) in mouse sperm under hypoxia (0, 0.5, 1, and 2.5 months) and posthypoxia reoxygenation (2.5 months of hypoxia followed by 2 or 4 months of reoxygenation). Ac-Tub and Glu-Tub levels, normalized to total α-tubulin levels, serve as biochemical indicators of microtubule stability. All the data are presented as the means ± SDs. In (A), n = 10 biologically independent mice per group; each data point represents one mouse; two-way ANOVA with Tukey's post hoc test; *P < 0.05, **P < 0.01, ***P < 0.001, ****P < 0.0001. In (B), n = 10 mice per group; ≥200 sperm scored per mouse; two-way ANOVA with Tukey's post hoc test; ****P < 0.0001. In (C-E), n = 10 fields from 3 biological replicates (microtubule regrowth); TEM defect rate: n = 10 mice, ≥200 cross-sections per group; unpaired two-tailed Student's t test; ****P < 0.0001. (F) shows a representative image from a single independent experiment. Ac-Tub and Glu-Tub signals were normalized to the total α-tubulin signal. No formal statistical testing was applied to this single replicate.

**Figure 3:**
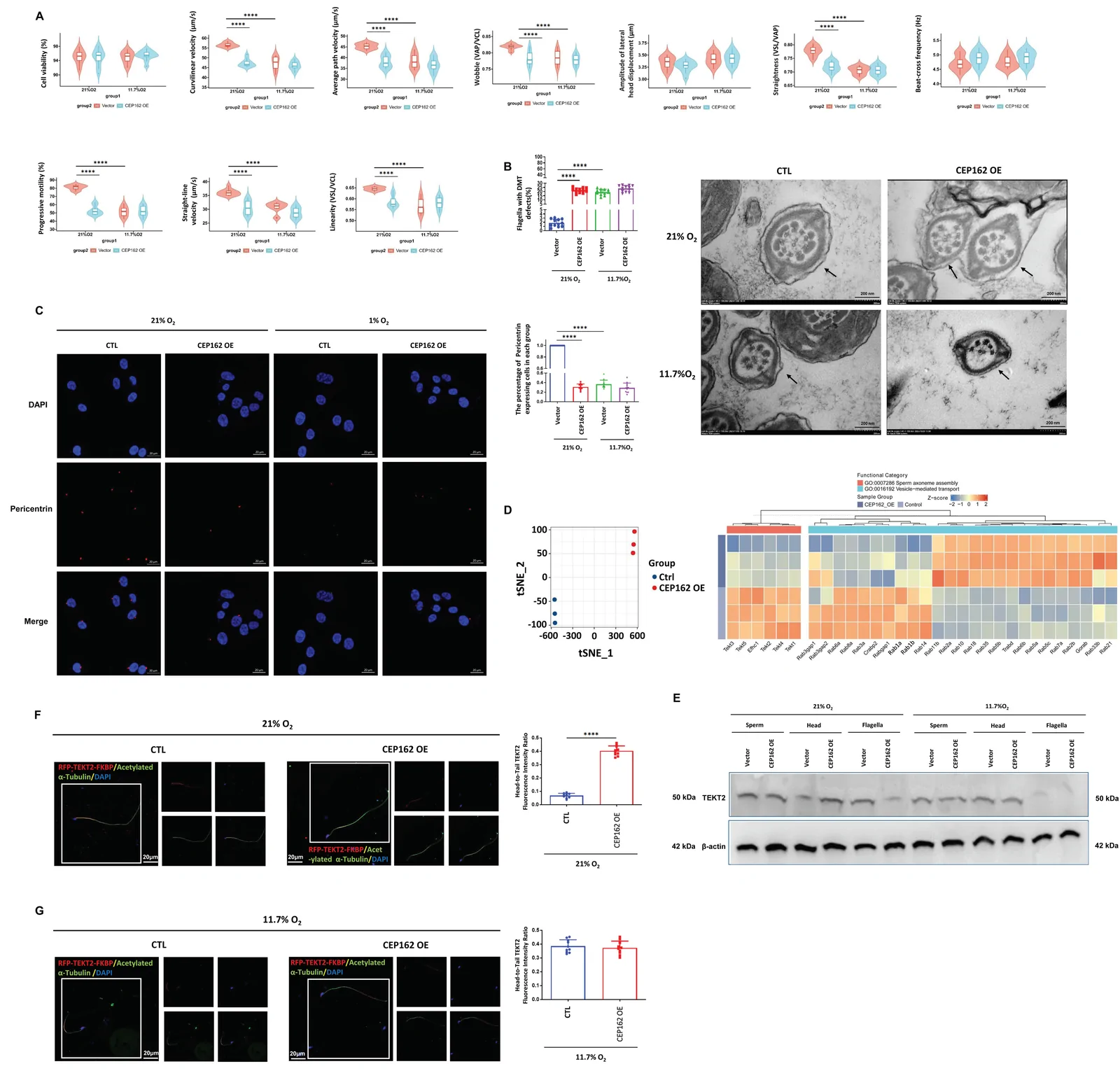
** Overexpressing CEP162 reduces sperm motility in mice. (A)** Sperm viability and kinematic analysis following CEP162 overexpression under normoxia (21% O_2_) and hypoxia (11.7% O_2_). The sperm motility parameters, including progressive motility, VCL, VSL, VAP, STR, BCF, ALH, LIN, and WOB, were quantified using computer-assisted sperm analysis (CASA; Microptic S.L., Barcelona, Spain).** (B)** Ultrastructural analysis of sperm flagella by transmission electron microscopy. Representative micrographs show transverse sections of the flagellum, including the midpiece (with a mitochondrial sheath), principal piece (with a fibrous sheath), and end piece (showing axonemal termination). All scale bars, 200 nm. The lower panel shows the quantification of axonemal DMT defects (%).** (C)** Microtubule regrowth following nocodazole washout in CEP162-overexpressing G2 cells. The percentage of pericentrin-expressing cells was calculated by dividing the number of pericentrin-positive cells by the total number of cells in each respective group. **(D)** Targeted proteomic profiling of CEP162-overexpressing sperm flagella. Heatmap showing Z score-normalized expression of selected TEKT family members involved in sperm axoneme assembly and Rab GTPases across three biological replicates. t-SNE visualization of the correlations among TEKT1, TEKT2, TEKT3, TEKT4, TEKT5, EFHC1, Rab1a, RAB1b, and RAB14 expression in the sperm flagella of control and CEP162-overexpressing mice. **(E)** Western blot analysis of TEKT2 protein expression in whole sperm and flagella isolated from CEP162-overexpressing mice maintained under normoxia (21% O_2_) or hypoxia (11.7% O_2_) for 10 weeks. Note: This Western blot detected endogenous TEKT2 (~50 kDa); the RFP-TEKT2-FKBP fusion protein (~87 kDa) was used exclusively for immunofluorescence localization in Panels F-G. **(F-G)** Mice were injected with recombinant AAV9 vectors encoding RFP-TEKT2-FKBP (~87 kDa) and maintained under **(F)** 21% O₂ (normoxia) or **(G)** 11.7% O₂ (hypoxia) for 10 weeks. Sperm were collected from the cauda epididymis and subjected to triple-label immunofluorescence for RFP (a fusion protein), acetylated α-tubulin (an axoneme marker), and DAPI (a nuclear counterstain). Imaging confirmed the flagellar localization of each RFP-tagged construct, with spatial resolution of the sperm head and flagellum enabled by DAPI counterstaining. Scale bar: 20 μm. Quantification of the head-to-tail TEKT2 fluorescence intensity ratio in the indicated groups. All the data are presented as the means ± SDs. In (A-C), n = 12 biologically independent mice (A-B) or n = 12 fields from 3 biological replicates (C); the data were normalized to the mean of the control group where indicated; two-way ANOVA with Tukey's post hoc test; ****P < 0.0001. In (D), n = 3 biological replicates; proteomic data are shown as Z scores. Differential protein expression was determined by two-tailed Student's t test with FDR correction (thresholds: *P* < 0.05, |log₂FC| > 1). (E) shows a representative image from a single independent experiment. The TEKT2 signal was normalized to that of β-actin (whole sperm or flagella). No formal statistical testing was applied to this single replicate. In (F-G), n = 10 biologically independent mice; ≥100 sperm scored per mouse for immunofluorescence analysis; the head-to-tail TEKT2 fluorescence intensity ratio was quantified using ImageJ; unpaired two-tailed Student's t test; ****P < 0.0001.

**Figure 4:**
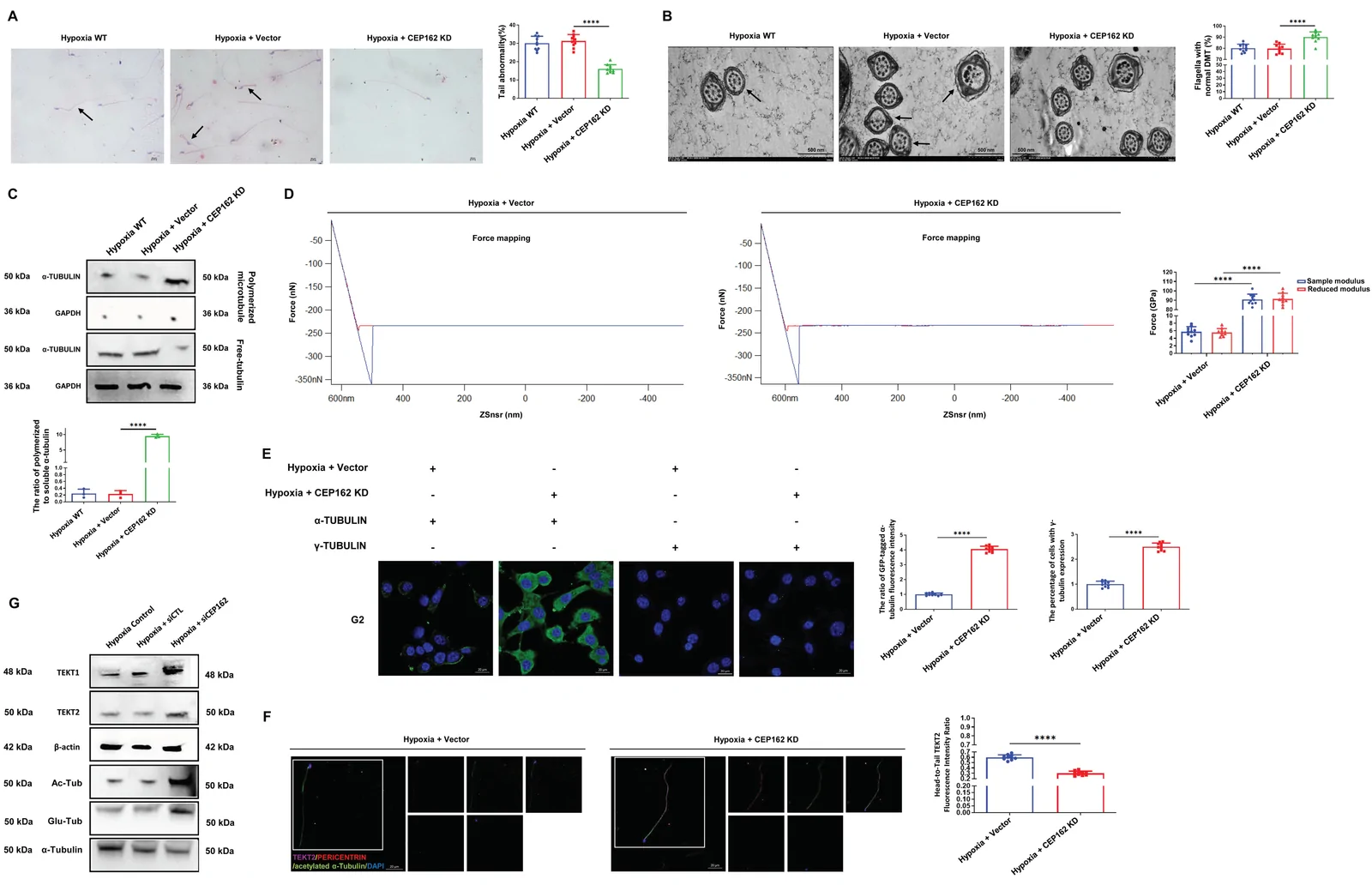
** CEP162 knockdown rescues hypoxia-induced defects in sperm flagella in mice. (A)** Papanicolaou staining was performed to observe sperm flagellar morphology in CEP162-knockdown mice exposed to 11.7% oxygen. Representative images are shown, and the percentage of sperm with abnormal tail morphology was quantified. Scale bar, 10 μm. **(B)** TEM images of sperm flagellar centrioles in CEP162-knockdown mice under hypoxic conditions. Representative cross-sectional TEM images of sperm flagella from mice with CEP162 knockdown after exposure to 11.7% oxygen. Scale bars, 500 nm. **(C)** Western blot analysis was performed to assess the levels of free tubulin and polymerized microtubules in sperm flagella isolated from *CEP162*-knockdown mice. The cell lysates were divided into soluble (S) and insoluble (P) fractions, which were enriched in free tubulin and polymerized microtubules, respectively. Immunoblotting was carried out using antibodies against α-tubulin and GAPDH to evaluate the distribution of tubulin species across the fractions. GAPDH, a soluble cytoplasmic protein, was used as a loading control for the soluble fraction. The ratio of polymerized to soluble α-tubulin was quantified from three biological replicates using ImageJ. **(D)** Representative adhesion force curves from force mapping measurements of flagella using silica tips. Force mapping enabled statistical analysis of flagellar adhesion through random sampling across the flagellar surface (scanning range: 40-60 µm). The x-axis (ZSnsr, Z-sensor) indicates the cantilever displacement relative to the sample surface. The red and blue traces represent the approach and retraction curves, respectively, showing probe-sample interactions during AFM scanning. The bar graph summarizes the sample and reduced modulus of the flagellar surface (n = 10). **(E)** Microtubule regrowth assay in *CEP162*-knockdown G2 cells following nocodazole washout under 1% O_2_. Representative images show the ratio of GFP-α-tubulin fluorescence intensity, reflecting the extent of microtubule polymerization. The percentage of cells exhibiting focused γ-tubulin puncta with associated radial α-tubulin asters was quantified as a functional readout of microtubule nucleation and regrowth. **(F)** Immunofluorescence analysis of TEKT2 localization and its colocalization with pericentrin in spermatozoa from CEP162-knockdown mice following exposure to hypoxia. Scale bar, 20 μm. **(G)** Western blot analysis of TEKT1, TEKT2, Ac-Tub, and Glu-Tub expression in sperm flagella from CEP162-knockdown mice exposed to hypoxia (11.7% O_2_). All the data are presented as the means ± SDs. In (A-B, D-E), n = 10 biologically independent mice (A, B, D) or n = 10 fields from 3 biological replicates (E); ≥200 sperm points were scored per group for morphology/TEM; one-way ANOVA with Tukey's post hoc test (A, B); two-way ANOVA with Tukey's post hoc test (D); unpaired two-tailed Student's t test (E); ****P < 0.0001. (C) The ratio of polymerized to soluble α-tubulin was quantified from 3 independent biological replicates using ImageJ; the data are presented as the means ± SDs; unpaired two-tailed Student's t test; ****P < 0.0001. In (F), n = 10 mice; colocalization assessed by visual inspection in ≥100 cells; unpaired two-tailed Student's t test; ****P < 0.0001. (G) shows a representative image from a single independent experiment. TEKT1 and TEKT2 signals were normalized to that of β-actin; Ac-Tub and Glu-Tub signals were normalized to that of total α-tubulin. No formal statistical testing was applied to this single replicate.

**Figure 5:**
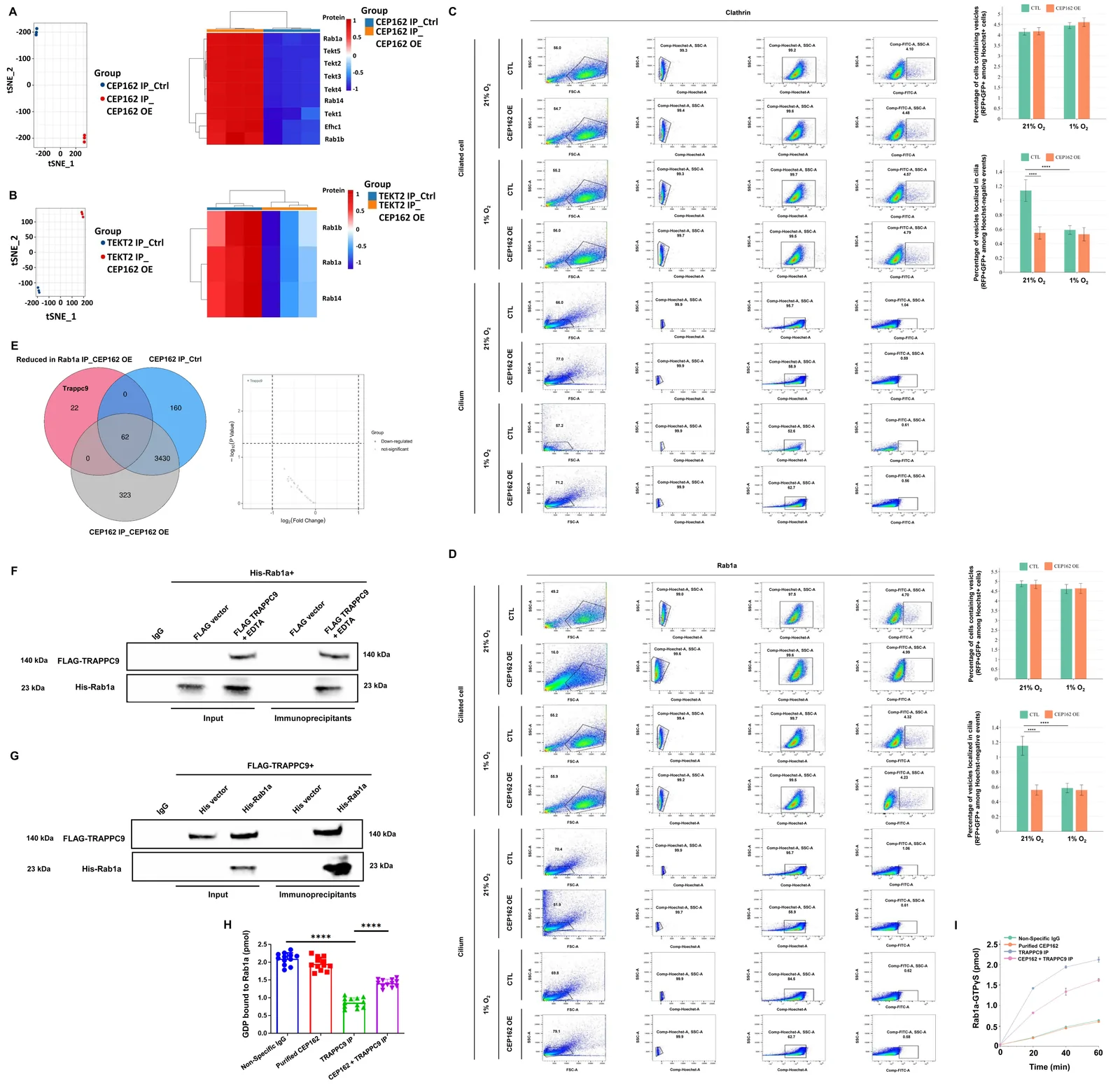
** Cep162 overexpression disrupts ciliary TEKT protein distribution and Rab1a-mediated vesicular trafficking by impairing TRAPPC9-dependent GTP loading. (A)** Heatmap showing the levels of TEKT1, TEKT2, TEKT3, TEKT4, TEKT5, EFHC1, Rab1a, RAB1b, and RAB14 immunoprecipitated (IP) with CEP162 in spermatogenic cells from control and CEP162-overexpressing mice. t-SNE visualization of the correlations among these proteins in the CEP162 IP samples. **(B)** Heatmap showing the levels of Rab1a, RAB1b, and RAB14 IP with TEKT2 in spermatogenic cells from control and CEP162-overexpressing mice. t-SNE visualization of the correlations among Rab1a, RAB1b, and RAB14 in the TEKT2 IP samples.** (C)** Flow cytometry was used to quantify the proportion of Clathrin and SSTR3 double-positive cells and cilia in IMCD3 cells overexpressing CEP162. Representative data show the proportion of cells containing vesicles (RFP+GFP+ among Hoechst+ events) and the proportion of vesicles localized in cilia (RFP+GFP+ among Hoechst-negative events). The fusion pore of the vesicle marker AP2 was labeled with GFP, while the ciliary marker SSTR3 was labeled with RFP. **(D)** Flow cytometry was used to quantify Rab1a and SSTR3 double-positive cells and cilia in CEP162-overexpressing IMCD3 cells. Representative data show the percentage of vesicle-containing cells (RFP^+^GFP^+^ among Hoechst^+^ cells) and cilia-localized vesicles (RFP^+^GFP^+^ among Hoechst^-^ cells). Rab1a (a vesicle recycling marker) was labeled with GFP, and SSTR3 (a ciliary marker) was labeled with RFP. **(E)** After CEP162 was overexpressed in spermatogenic cells, the proteins whose expression was reduced according to Rab1a immunoprecipitation (RAB1A IP_CEP162 OE) were intersected with those identified in control and CEP162-overexpressing CEP162 immunoprecipitates (CEP162 IP_Ctrl and CEP162 IP_CEP162 OE), as determined by Venn diagram analysis. This intersection highlights proteins that are reduced in the RAB1A interactome upon CEP162 overexpression but are absent from both CEP162 IP groups, thereby excluding direct CEP162 binders and identifying indirect regulators of RAB1A, such as TRAPPC9. **(F)** Lysates from G2 cells expressing the indicated proteins were incubated with GTPγS, GDP, or EDTA (to chelate Mg^2+^ and induce “nucleotide-free” conditions^24^), and Rab1a-His coimmunoprecipitated with FLAG-TRAPPC9 only in the EDTA-treated sample. **(G)** Coimmunoprecipitation of FLAG-TRAPPC9 with Rab1a-His under nucleotide-free conditions. **(H)** TRAPPC9-stimulated guanine nucleotide exchange activity was measured using a purified Rab1a-His fusion protein preloaded with [³H]GDP and quantified by monitoring the time-dependent release of [³H]GDP from Rab1a in nonspecific IgG, purified CEP162, TRAPPC9, and CEP162 + TRAPPC9 immunoprecipitates (n = 12). **(I)** Nucleotide exchange activity measured by the time-dependent binding of [35S]GTPγS to Rab1a in immunoprecipitates. Nonspecific IgG, purified CEP162, TRAPPC9 IP, and CEP162 + TRAPPC9 IP were tested; the systems were incubated at 37°C for the indicated durations, after which nitrocellulose filter binding was performed to quantify the incorporation of GTPγS. All the data are presented as the means ± SDs. In (A-B, E), n = 3 biological replicates; proteomic data are shown as Z scores or raw intensities. In (C, D), n = 12 biological replicates (independent IMCD3 cultures); two-way ANOVA with Tukey's post hoc test; ****P < 0.0001. (F, G) show representative images from a single independent experiment. No formal statistical testing was applied to this single replicate. In (H-I), n = 12 (L) or n = 10 (M) time-course replicates; the data were normalized to the baseline; two-way ANOVA; ****P < 0.0001.

**Figure 6:**
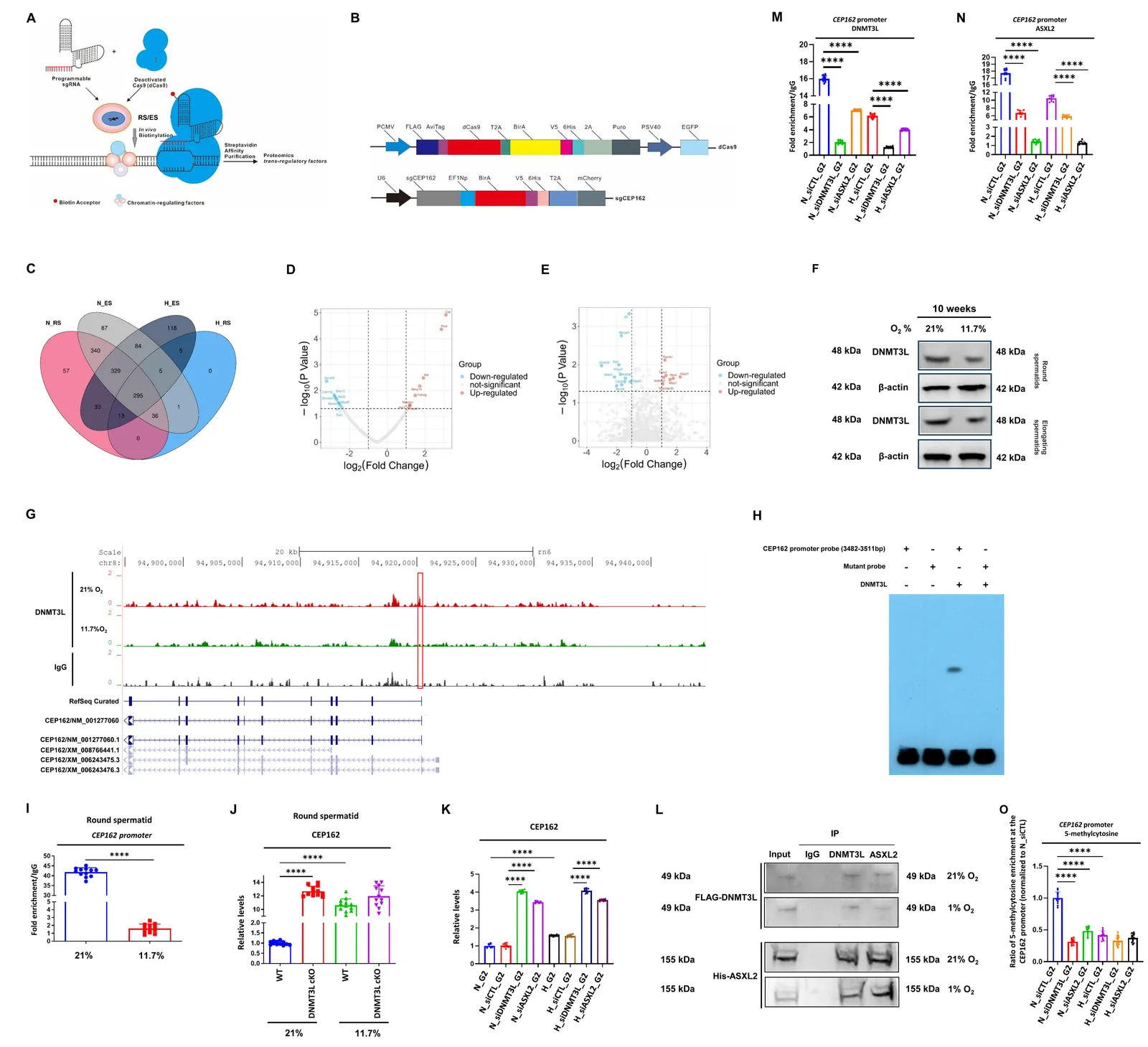
** Hypoxia induces CEP162 expression through the epigenetic silencing of DNMT3L and ASXL2. (A)** Schematic of the dCas9-mediated capture of chromatin interactions at the CEP162 promoter region. This figure depicts the experimental strategy for identifying proteins that are differentially enriched at the CEP162 promoter under specific conditions (e.g., hypoxia). The approach uses dCas9-mediated chromatin capture: a programmable sgRNA targets the CEP162 promoter, recruits dCas9 to the locus, undergoes *in vivo* biotinylation, and is purified via streptavidin affinity. Purified complexes were analyzed by proteomics to identify interacting trans-regulatory factors, providing insights into the epigenetic and transcriptional regulation of CEP162 expression. **(B)** The CAPTURE system for dCas9-mediated chromatin interaction capture consists of two main components: (1) FB-dCas9, a fusion protein containing functional domains for targeting and purification, and (2) target-specific sgRNAs designed to guide dCas9 to the desired genomic locus. The FB-dCas9 construct includes multiple tags and modules for efficient expression, localization, and purification, while the sgRNA is engineered to specifically target the region of interest. Together, these components enable the precise recruitment of dCas9 to target DNA, facilitating the capture and identification of interacting proteins and chromatin regulatory factors. **(C)** Venn diagram of proteins differentially enriched at the CEP162 promoter region in RS and ES under normoxic (N) and hypoxic (H) conditions. Volcano plot of differentially enriched proteins with |log₂(FC)| ≥ 1 identified by dCas9-mediated chromatin capture in **(D)** RS and **(E)** ES. Proteins with |log₂(FC)| ≥ 1 and P < 0.05 are highlighted in red (upregulated) or blue (downregulated), while proteins without significant changes are shown in gray. **(F)** Western blot analysis of DNMT3L protein expression in round and elongating spermatids exposed to 21% and 11.7% oxygen for 10 weeks. **(G)** CUT&Tag-seq analysis of DNMT3L binding to the CEP162 promoter in RS under 21% O_2_ (red) and 11.7% O_2_ (green). **(H) An** EMSA was performed to evaluate the binding of DNMT3L to the CEP162 promoter region at specific regions: +222 to +251 bp relative to the transcription start site (TSS). **(I)** Chromatin immunoprecipitation (ChIP) followed by quantitative PCR (qPCR) analysis of DNMT3L binding to the CEP162 promoter in round spermatids from rats exposed to 21% or 11.7% oxygen for 10 weeks. **(J)** qPCR analysis of CEP162 mRNA expression levels in round spermatids from WT and DNMT3L-cKO mice exposed to 21% or 11.7% oxygen for 10 weeks. **(K)** qPCR analysis of the mRNA levels of CEP162 in control and DNMT3L/ASXL2-deficient G2 cells. **(L)** Co-IP analysis of the interaction between FLAG-DNMT3L and HIS-ASXL2 in G2 cells transfected with the respective plasmids and exposed to 21% or 1% oxygen. ChIP‒qPCR analysis of DNMT3L **(M)** and ASXL2 **(N)** binding to the CEP162 promoter in G2 cells following treatment with control siRNA, DNMT3L siRNA, or ASXL2 siRNA. **(O)** 5-Methylcytosine (5-mC) enrichment at the CEP162 promoter in G2 cells under normoxia (N) or hypoxia (H) following knockdown of DNMT3L or ASXL2. All the data are presented as the means ± SDs. In (A-E), n = 3 biological replicates for CAPTURE proteomics; data are shown as log_2_ fold change and P values; no additional statistical testing was performed beyond proteomic analysis. (F) Representative image from a single independent experiment. The DNMT3L signal was normalized to that of β-actin. No formal statistical testing was applied to this single replicate. (G, H, L) Representative images from a single independent experiment. No formal statistical testing was applied to this single replicate. In (I, K, M-N), n = 12 biological replicates (spermatids or independently transfected G2 cell cultures); mRNA or ChIP signals were normalized to that of β-actin (qPCR) or input DNA (ChIP); unpaired two-tailed Student's t test (I) or two-way ANOVA with Tukey's post hoc test (K, M, N); ****P < 0.0001. In (J), n = 12 mice per genotype; CEP162 mRNA expression was normalized to that of β-actin; two-way ANOVA with Tukey's post hoc test; ****P < 0.0001. In (O), n = 12 biological replicates per group, normalized to the N_siCTL control (set as 1.0); one-way ANOVA with Tukey's post hoc test; ****P < 0.0001 for comparisons between N_siCTL and H_siCTL, N_siCTL and N_siDNMT3L, and N_siCTL and N_siASXL2.

**Figure 7:**
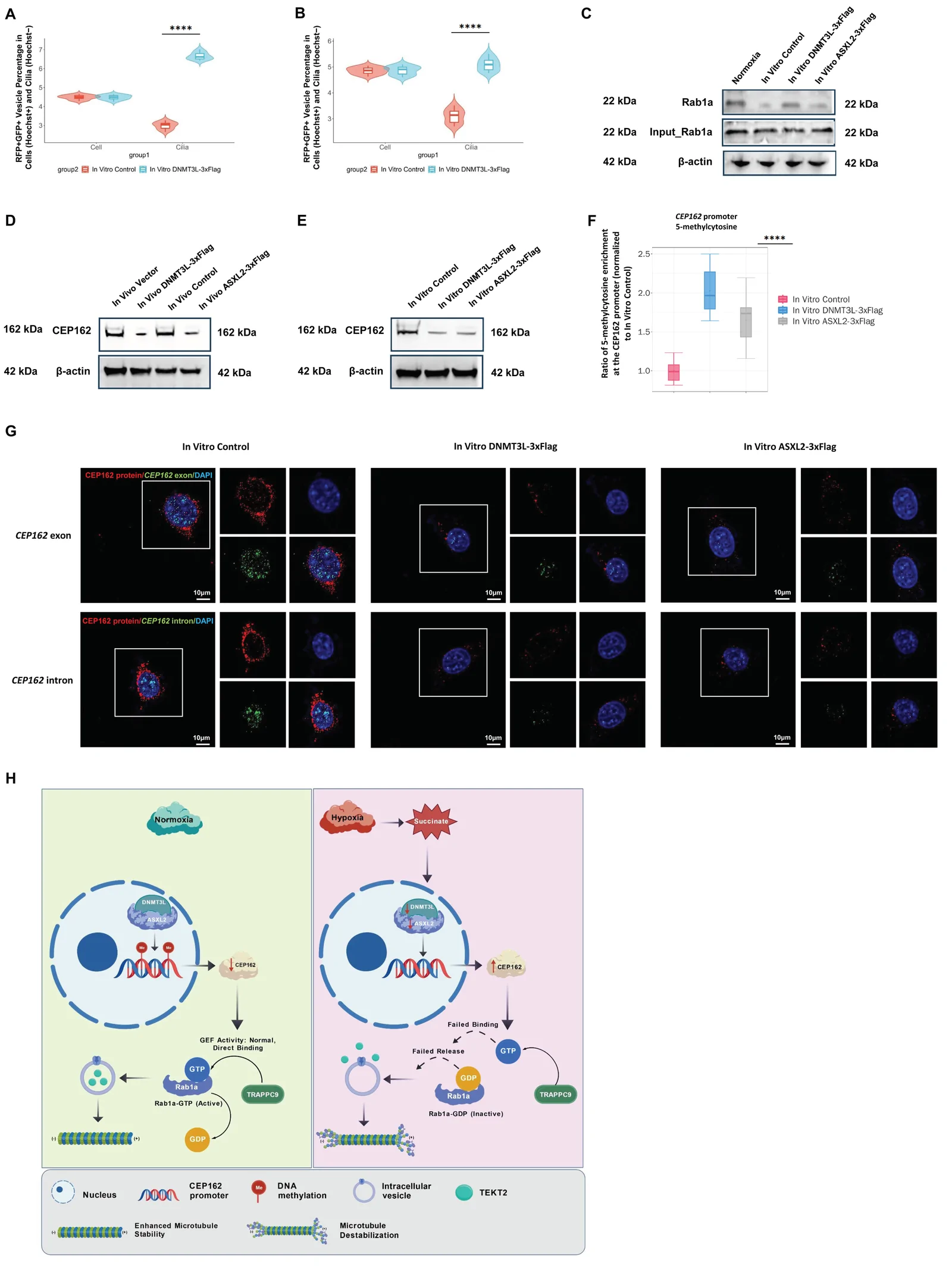
** DNMT3L and ASXL2 ameliorate hypoxia-induced vesicular trafficking defects by epigenetically silencing CEP162 expression and activating Rab1a. (A)** Flow cytometry quantification of Clathrin and SSTR3 double-positive IMCD3 cells and cilia under hypoxia. The data show the proportion of vesicle-containing cells (RFP^+^GFP^+^ among Hoechst^+^) and cilia-localized vesicles (RFP^+^GFP^+^ among Hoechst^-^ cells) in the *in vitro* control and *in vitro* DNMT3L-3×Flag groups. Clathrin (a vesicle fusion pore marker) was labeled with GFP, and SSTR3 (a ciliary marker) was labeled with RFP. **(B)** Flow cytometry analysis of the colocalization of Rab1a (GFP) and SSTR3 (RFP) in cilia under hypoxia. Quantification of vesicle-positive cells and ciliary vesicle localization in control and DNMT3L-3×Flag-expressing cells revealed altered vesicle trafficking upon DNMT3L overexpression. **(C)** Rab1a activation was assessed by a GST-TRAPPC9 (Rab1a-binding domain) pull-down assay under hypoxic conditions. Lysates from G2 cells transfected with *in vitro* control, *in vitro* DNMT3L-3×Flag, or *in vitro* ASXL2-3×Flag were incubated with GST-TRAPPC9 bound to glutathione-Sepharose beads to specifically isolate active Rab1a-GTP. Western blotting of both the pulled-down and input fractions was performed using an anti-Rab1a antibody. **(D)** Western blot analysis of CEP162 protein levels in round spermatids from *in vivo* control, vector, DNMT3L-3×Flag, and ASXL2-3×Flag mice exposed to hypoxia. **(E)** CEP162 protein expression was analyzed by Western blotting in G2 cells under hypoxia following *in vitro* control, DNMT3L-3×Flag, or ASXL2-3×Flag treatment. **(F)** 5-mC enrichment at the CEP162 promoter was analyzed by ChIP‒qPCR in G2 cells under hypoxia in the *in vitro* control, DNMT3L-3×Flag, and ASXL2-3×Flag groups. **(G)** IF/smFISH detection of CEP162 pre-mRNA (intron and exon probes) and the CEP162 protein in G2 cells under hypoxia in the *in vitro* control, DNMT3L-3×Flag, and ASXL2-3×Flag groups.** (H)** Schematic model illustrating the mechanism by which hypoxia disrupts sperm flagellar development through the DNMT3L/ASXL2-mediated epigenetic regulation of CEP162. Under normoxic conditions, the DNMT3L-ASXL2 complex binds to the CEP162 promoter, promoting DNA methylation and transcriptional repression, resulting in low CEP162 expression, which allows TRAPPC9 to facilitate GTP loading onto Rab1a; active Rab1a-GTP drives vesicle trafficking and supports vesicle docking and recycling at the flagellar base, ensuring the efficient delivery of axonemal components such as TEKT2 for proper microtubule assembly and structural integrity. Under hypoxic conditions, oxygen deprivation inhibits succinate dehydrogenase activity, leading to intracellular succinate accumulation. This metabolic shift competitively inhibits α-KG-dependent dioxygenases, resulting in functional impairment and transcriptional downregulation of the DNMT3L-ASXL2 complex, subsequent CEP162 promoter hypomethylation, and CEP162 overexpression. The aberrant accumulation of CEP162 impairs TRAPPC9-mediated Rab1a activation, consequently disrupting vesicular transport and ultimately compromising microtubule organization and flagellar formation. All the data are presented as the means ± SDs. In (A-B), n = 12 biological replicates (independent IMCD3 cell cultures); one-way ANOVA with Tukey's post hoc test; ****P < 0.0001. (C) Representative image from a single independent experiment. The Rab1a-GTP signal was normalized to the total Rab1a signal (Input). No formal statistical testing was applied to this single replicate. (D-E) Representative image from a single independent experiment. The CEP162 signal was normalized to that of β-actin. No formal statistical testing was applied to this single replicate. In (F), n = 12 biological replicates (independent G2 cell transfections); 5-mC enrichment was normalized to input DNA; one-way ANOVA with Tukey's post hoc test; ****P < 0.0001. (G) Representative results of n = 3 independent experiments; pre-mRNA and protein expression was visualized by smFISH/IF.
