# Supplementary material for: Hypoxia Reprograms a Ciliary Scaffold into a Metabolic-Epigenetic Trafficking Hub via a Succinate-Driven DNMT3L-ASXL2 Axis
Source: Int J Biol Sci. 2026 Oct 9;22(15):8707–38. doi: 10.7150/ijbs.137355 (PMC13686052; doi:10.7150/ijbs.137355)
Supplement: Supplementary file 1 [file ijbsv22p8707s1.pdf]

Supplementary Figures for

Hypoxia Reprograms a Ciliary Scaffold into a Metabolic–Epigenetic Trafficking Hub via a Succinate-Driven DNMT3L–ASXL2 Axis

Jun Yin[1,2,3†*], Zhifeng Zhong[4†], Mengjie Zhang[1,2,3†], Debao Li[5], Aiping Wang[6], Qianying Huang[7], Qinghua Zhang[8*], Bing Ni[1,2,3*], Wei He[9*]

[1] Department of Pathophysiology, Army Medical University, Chongqing 400038, China

[2] Key Laboratory of Extreme Environmental Medicine, Ministry of Education of China, Chongqing 400038, China

[3] Key Laboratory of High Altitude Medicine, PLA, Chongqing 400038, China

[4] Department of High Altitude Operational Medicine, College of High Altitude Military Medicine, Army Medical University, Chongqing 400038, China

[5] Department of Immunology, School of Basic Medical Sciences, Southern Medical University, Guangzhou 510515, China

[6] Department of Frigid Zone Medicine, College of High Altitude Military Medicine, Army Medical University, Chongqing 400038, China

[7] The Clinical Research Center, Southwest Hospital, Army Medical University, Chongqing 400038, China

[8] Department of Obstetrics and Gynecology, Daping Hospital, Army Medical University, Chongqing 400042, China.

[9] Reproductive Medical Center, Southwest Hospital, Army Medical University, Chongqing 400038, China

† These authors contributed equally.

* To whom correspondence should be addressed:

Jun Yin, Tel: +86-23-68771745; email: yinjun7155@sina.com; ORCID ID: 0000-0002-7541-1088

Qinghua Zhang: +86-23-68729211; email: zhangqh@tmmu.edu.cn

Bing Ni, Tel: +86-23-68771740; email: nibing@tmmu.edu.cn; ORCID ID: 0000-0002-4297-

5346
Wei He, Tel: +86-23-13608356955; email: anyhewei@tmmu.edu.cn

This document includes the following:

Figures S1 to S7

Supplementary Figure 1

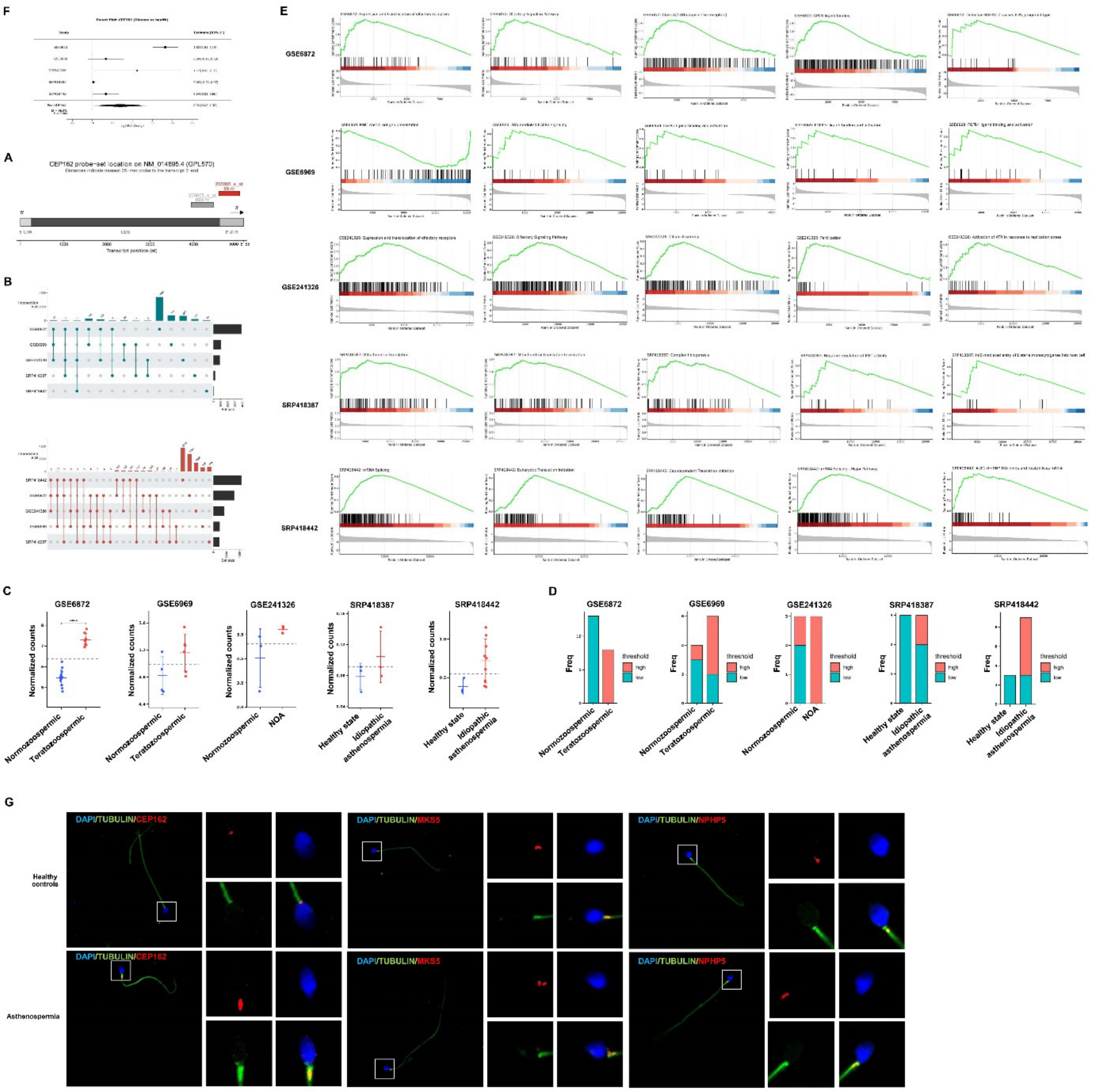

Supplementary Figure 1. Analysis of transcriptomic data from healthy and asthenospermic/NOA/teratozoospermic individuals across multiple studies. (A) CEP162 probe set selection on GPL570 (Affymetrix U133 Plus 2.0). Schematic of the CEP162 RefSeq transcript NM_014895.4 (5,015 nt) showing two probe sets: 206006_s_at (selected representative, red; annotated to NM_014895.4, nearest probe 88 nt from the 3′ end) and 206005_s_at (alternative, gray; annotated to AK023613, 690 nt from the 3′ end). **(B)** UpSet plots of differentially expressed genes across five male infertility cohorts. Overlap of nominally upregulated (top) and

downregulated (bottom) genes (nominal $P < 0.05$) in GSE6872, GSE6969, GSE241326, SRP418387, and SRP418442. Vertical bars indicate intersection sizes; connected dots denote contributing datasets. The right horizontal bars show the total number of differentially expressed genes per cohort. **(C)** CEP162 expression analysis across five independent cohorts. Transcriptomic data were obtained from GSE6872 (Affymetrix; normozoospermia, n = 13; teratozoospermia, n = 8), GSE6969 (Illumina; normozoospermia, n = 9; teratozoospermia, n = 14), GSE241326 (RNA-seq; normozoospermia, n = 3; nonobstructive azoospermia, n = 3), SRP418387 (Illumina RNA-seq; control, n = 3; asthenozoospermia, n = 3), and SRP418442 (Illumina RNA-seq; control, n = 3; asthenozoospermia, n = 9). The expression values were normalized using platform-specific methods (RMA for microarrays and TPM/FPKM for RNA-seq). **(D)** Patient stratification by CEP162 expression across male infertility cohorts. Patients were stratified into high- and low-expression groups on the basis of the mean CEP162 expression levels across five independent transcriptomic datasets: teratozoospermia (GSE6872: n = 13 controls, n = 8 cases; GSE6969: n = 9 controls, n = 14 cases; $\log_2$(Cy5/Cy3) normalized), nonobstructive azoospermia (GSE241326: n = 3 controls, n = 3 cases; count normalized), and idiopathic asthenozoospermia (SRP418387: n = 3 controls, n = 3 cases; and SRP418442: n = 3 controls, n = 9 cases; RNA-seq). **(E)** Reactome GSEA of commonly upregulated genes. Pathway enrichment was assessed across CEP162 upregulated datasets. **(F)** Meta-analysis of CEP162 expression across five male infertility cohorts. Forest plot showing $\log_2$ fold-change estimates for CEP162 in GSE6872, GSE6969, GSE241326, SRP418387, and SRP418442. The microarray datasets used representative probes (CEP162: GSE6872, 206006_s_at; and GSE6969, GI_7662445-S), and the RNA-seq datasets used gene-level normalized expression. Pooled effect size (random-effects model), 95% confidence interval, heterogeneity (I^2), and pooled P value are indicated. **(G)** Immunofluorescence staining images of human sperm from healthy controls and patients with asthenozoospermia. The localization of CEP162, MKS5, and NPHP5 was assessed using specific antibodies. Acetylated α-tubulin (axonemal marker) and DAPI (nuclear counterstain) were used as structural references. Scale bar, 5 μm.

All the data are presented as the mean ± SD.

(A-B) Bioinformatic schematics and descriptive visualizations. Panel (A) illustrates probe-set positioning, and panel (B) displays the overlap of differentially expressed genes across cohorts

using UpSet plots. No formal statistical hypothesis testing was applied to these descriptive panels.

(C) GSE6872 (n = 13 controls; n = 8 patients), GSE6969 (n = 9 controls; n = 14 patients), GSE241326/SRP418387 (n = 3 per group), and SRP418442 (n = 3 controls; n = 9 patients). The microarray data were normalized using platform-specific methods (RMA or dChip), and the RNA-seq data were processed through standard pipelines (TPM/FPKM). Statistical significance was assessed by an unpaired two-tailed Student's t test (GSE241326 and SRP418387) or one-way ANOVA with Tukey's post hoc test (GSE6872, GSE6969, and SRP418442); expression stratification (high vs. low) was based on cohort-wide mean expression; no additional statistical testing was applied for stratification.

(D) Descriptive patient stratification based on cohort-wide mean expression levels (high vs. low). No additional statistical hypothesis testing was applied to this categorical stratification.

(E) Only pathways with NES > 1.3 and FDR q < 0.15 were retained. The combined top 5 Reactome pathways are shown for datasets meeting the relevant thresholds (GSE6969: none retained); the full results are shown in Supplementary Table 2.

(F) A meta-analysis was performed using a random-effects model to pool $\log_2$ fold-change estimates. Statistical significance is indicated by the pooled *P* value, and between-study heterogeneity is quantified by the I^2 statistic, as detailed in the respective forest plot.

(G) Representative images from ≥3 independent immunofluorescence experiments; localization was assessed by visual inspection of ≥50 sperm per group from n = 5 healthy controls and n = 5 patients with asthenozoospermia; no quantitative statistical analysis was performed.

Supplementary Figure 2

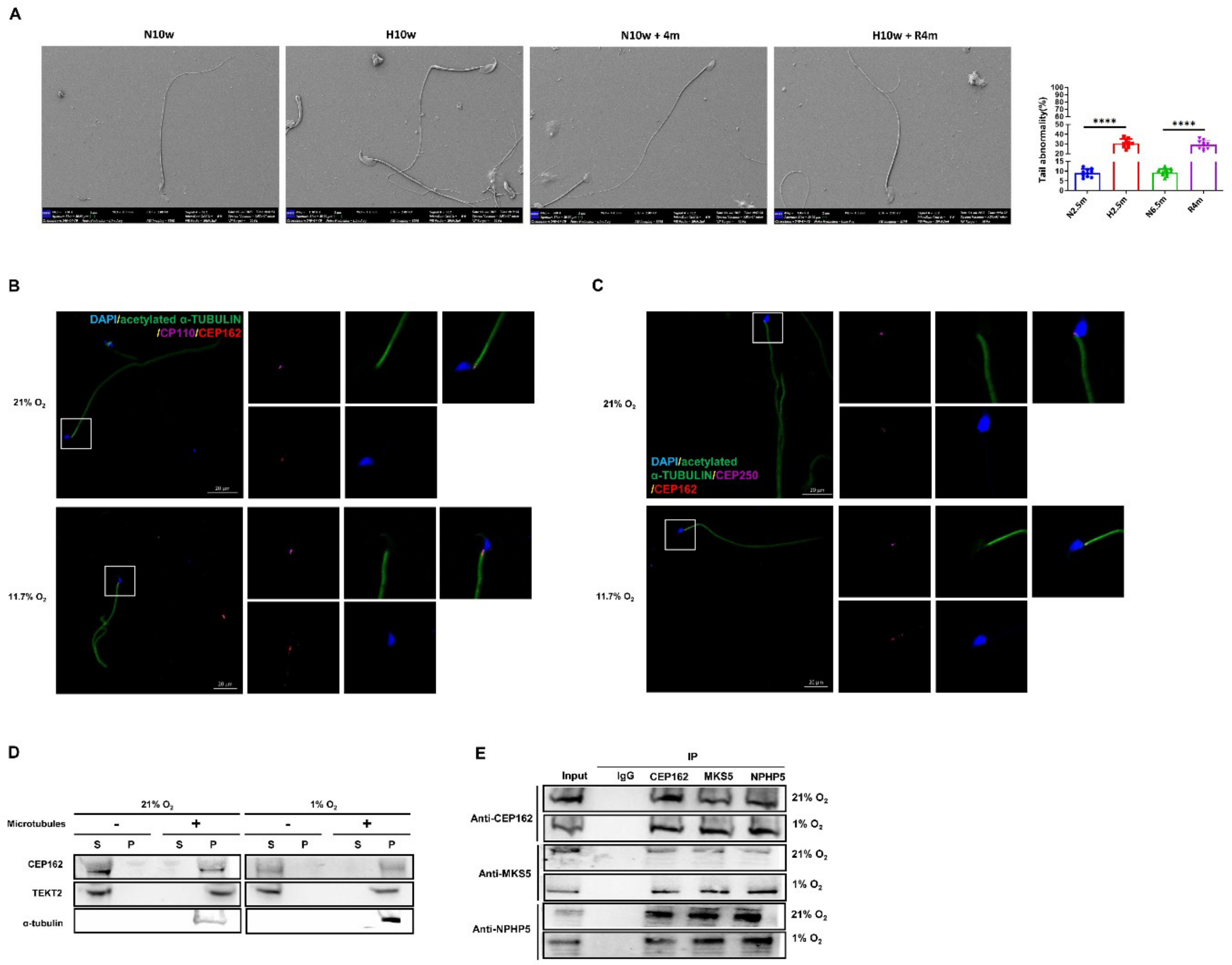

Supplementary Figure 2. Cep162 is associated with microtubules and the transition zone proteins MKS5 and NPHP5 and is localized to the basal body of the sperm flagella under both normoxia and hypoxia. (A) Scanning electron microscopy (SEM) images of sperm with flagellar abnormalities from mice exposed to chronic hypoxia (10 weeks) and posthypoxia recovery (4 months of reoxygenation). **(B)** Immunofluorescence analysis of CEP162 colocalization with **(B)** CEP250 and **(C)** CP110 in sperm. Flagella (acetylated α-tubulin) and nuclei (DAPI) are shown for orientation. **(D)** A microtubule cosedimentation assay was performed to examine the interaction between CEP162 and α-tubulin under 21% O_2 and 1% O_2 conditions. **(E)** Analysis of CEP162 interactions with MKS5 and NPHP5 by coimmunoprecipitation under 21% O_2 and 1% O_2 conditions. All the data are presented as the mean ± SD.

In (A), n = 10 biologically independent mice per group; ≥100 sperm scored per mouse; two-way ANOVA with Tukey's post hoc test; ****P < 0.0001.

In (B–C), n = 3 independent experiments; colocalization was assessed in ≥50 sperm per group; quantification was not performed.

(D–E) Representative images from a single independent experiment. No formal statistical testing was applied to this single replicate.

Supplementary Figure 3a

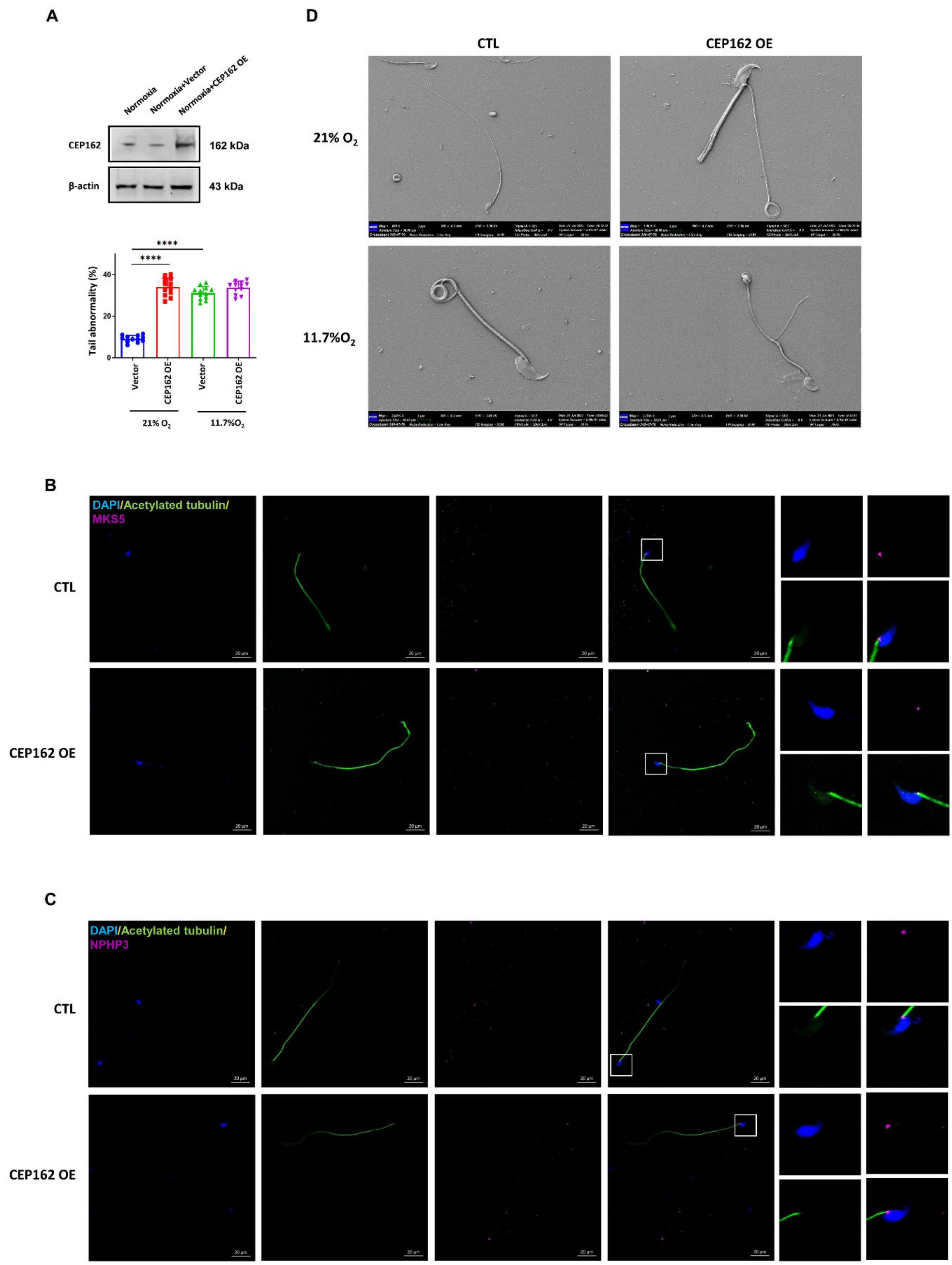

Supplementary Figure 3a(A-D). CEP162 overexpression disrupts sperm flagellar structure and microtubule organization in vivo. (A) Western blot analysis of CEP162 protein levels in spermatogenic cells isolated from the testes of CTL- and CEP162-overexpressing mice. Immunofluorescence staining of MKS5 **(B)** and NPHP3 **(C)** at the ciliary/flagellar base in control

(CTL) and CEP162-overexpressing (CEP162 OE) cells. **(D)** Morphological analysis of sperm flagellar abnormalities in CEP162-overexpressing mice under hypoxic conditions by scanning electron microscopy. The histogram represents the results of the quantitative assessment of sperm tail abnormalities.

All the data are presented as the mean ± SD.

(A) Representative image from a single independent experiment; protein signals were normalized to the loading control (β-actin). No formal statistical testing was applied to this single replicate.

In (B), n = 12 biologically independent mice per group; ≥200 sperm were scored per mouse; representative images are shown; no statistical analysis was performed.

In (C), n = 12 biologically independent mice per group; ≥200 sperm were scored per mouse; representative images are shown; no statistical analysis was performed.

In (D), n = 12 biologically independent mice per group; ≥200 sperm were scored per mouse; two-way ANOVA with Tukey's post hoc test; ****P < 0.0001.

Supplementary Figure 3b

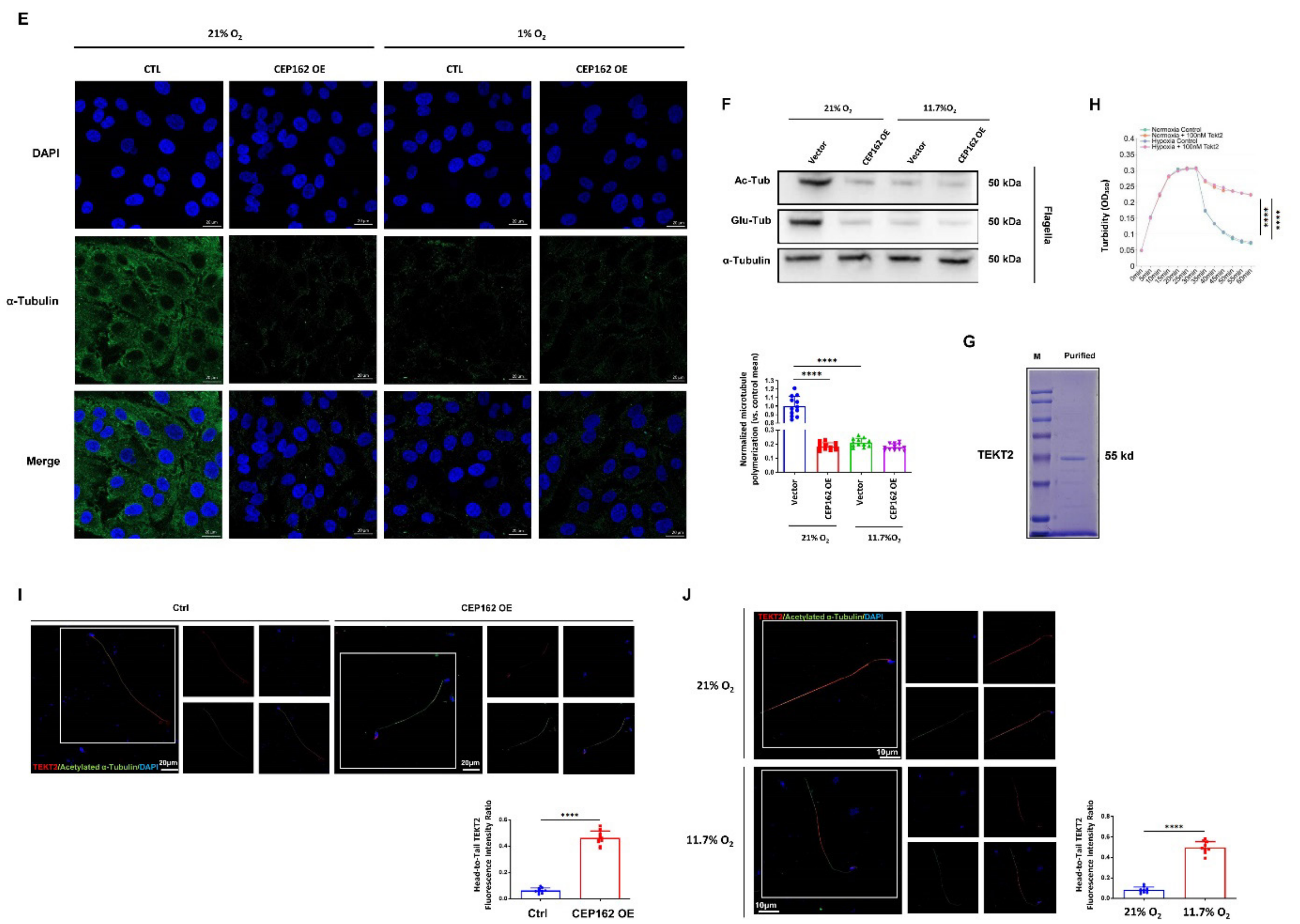

Supplementary Figure 3b(E-J). CEP162 overexpression disrupts microtubule stability and ciliary protein trafficking, whereas recombinant TEKT2 stabilizes microtubules in vitro. (E) Microtubule regrowth following nocodazole washout in CEP162-overexpressing G2 cells. GFP–α-tubulin fluorescence intensity, reflecting microtubule regrowth or polymerization, was quantified. **(F)** Western blot showing Ac-Tub and Glu-Tub levels in whole sperm and flagella isolated from CEP162-overexpressing mice after 10 weeks of normoxia (21% O_2) or hypoxia (11.7% O_2). The decreased levels of Ac-Tub and Glu-Tub in the flagella suggest that CEP162 induced the destabilization of axonemal microtubules. α-Tubulin was used as a loading and normalization control. **(G)** SDS–PAGE analysis of recombinant tekt2 protein expression and purification. Lane M: protein molecular weight marker; Lane “Purified”: purified tekt2 protein following Ni-NTA affinity chromatography, demonstrating high purity with a single band at ~55 kDa. **(H)** Real-time turbidity assay (OD_{350}) results to monitor microtubule depolymerization upon a shift from 37°C to 4°C under normoxia (21% O_2) or hypoxia (1% O_2) with or without 100 nM purified TEKT2. Quantification of the ciliary fluorescence intensity of TEKT2 from confocal z-stacks using ImageJ. **(I)** Representative immunofluorescence images of endogenous TEKT2 in sperm from control and CEP162-overexpressing (CEP162 OE) mice. Acetylated α-tubulin and DAPI indicate the axoneme and nuclei, respectively. Scale bar, 20 μm. Quantification of the head-to-tail TEKT2 fluorescence intensity ratio. **(J)** Immunofluorescence analysis of TEKT2 distribution in the sperm heads and flagella of mice following 10 weeks of hypoxia exposure (n = 10). Scale bars, 5 μm.

All the data are presented as the mean ± SD.

In (E), n = 12 fields from 3 biological replicates; GFP–α-tubulin intensity normalized to the mean of the control; two-way ANOVA with Tukey’s post hoc test; ****$P < 0.0001$.

(F–G) Representative images from a single independent experiment; protein signals were normalized to the loading control (total α-tubulin). No formal statistical testing was applied to this single replicate.

In (H), n = 3 biological replicates, with each replicate measured in technical triplicate at each time point. The final OD_{350} and depolymerization slope were used as readouts of microtubule stability; ****$P < 0.0001$ for comparisons between Normoxia Control vs. Normoxia + TEKT2 and between Hypoxia Control vs. Hypoxia + TEKT2 (two-way ANOVA with Tukey’s post hoc test).

In (I), n = 12 biologically independent mice; ≥100 sperm scored per mouse for immunofluorescence analysis; head-to-tail TEKT2 fluorescence intensity ratio quantified using ImageJ; unpaired two-tailed Student’s t test; ****$P < 0.0001$.

In (J), n = 10 biologically independent mice; ≥100 sperm scored per mouse for immunofluorescence analysis; head-to-tail TEKT2 fluorescence intensity ratio quantified using ImageJ; unpaired two-tailed Student’s t test; ****$P < 0.0001$.

Supplementary Figure 4a

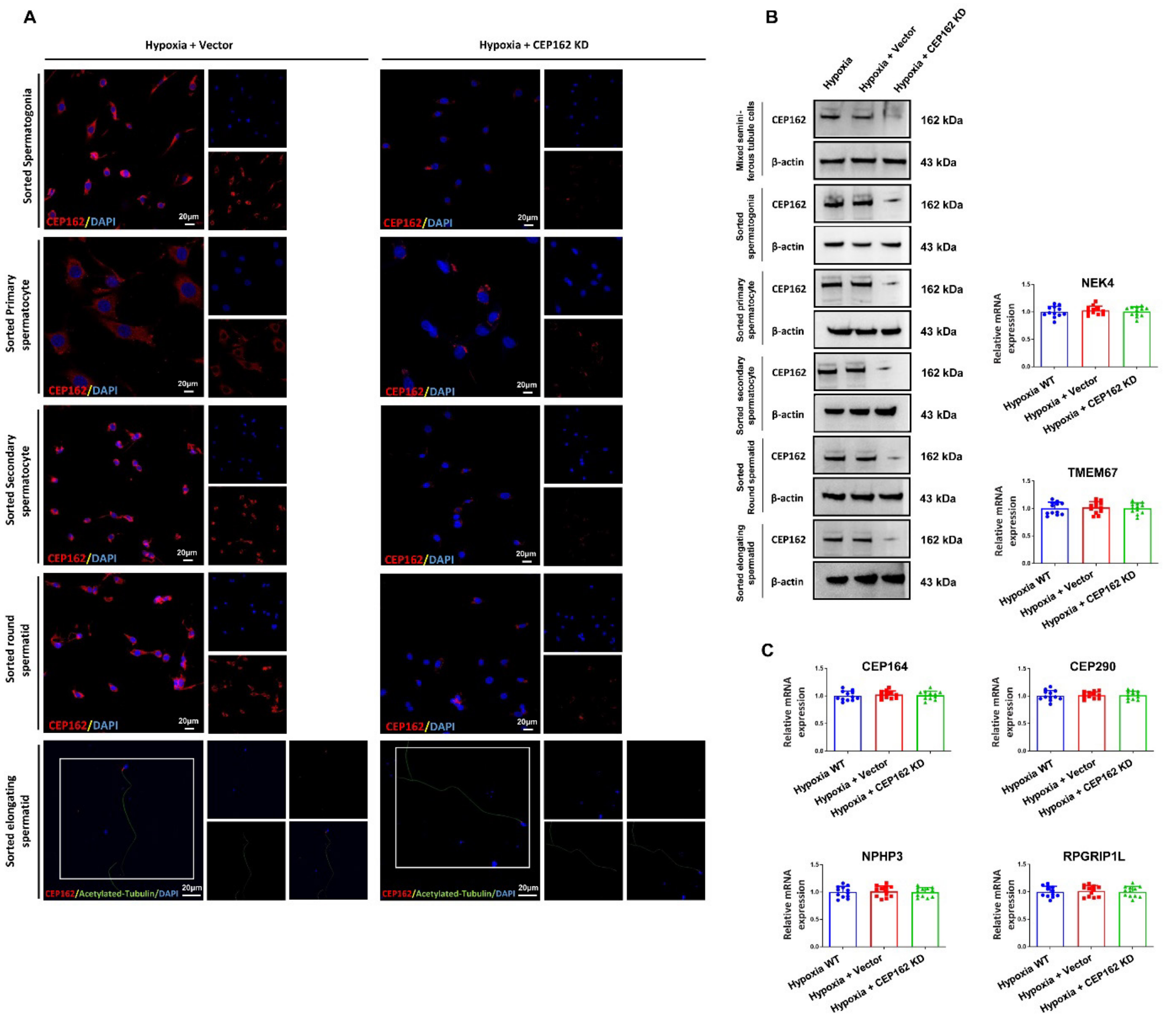

Supplementary Figure 4a(A-C). CEP162 knockdown under hypoxia reduces its expression and modulates related ciliary/centrosomal gene transcription in spermatogenic cells. (A) Immunofluorescence staining showing CEP162 protein expression in spermatogenic cells from mouse testes from the Hypoxia + Vector and Hypoxia + CEP162 KD groups.

(B) Western blot analysis of CEP162 protein levels in spermatogenic cells isolated from the testes of mice in the Hypoxia + Vector and Hypoxia + CEP162 KD groups. **(C)** Quantification of the relative mRNA expression levels of CEP164, CEP290, NEK4, NPHP3, RPGRIP1L, and TMEM67 in spermatogenic cells from the Hypoxia + Vector and Hypoxia + CEP162 knockdown (KD) groups.

All the data are presented as the mean ± SD.

(A) Representative images from n = 3 biologically independent mice; protein expression was assessed by visual inspection in ≥50 cells per group; no formal statistical analyses were performed for these qualitative imaging assays.

(B) Representative image from a single independent experiment; protein signals were normalized to that of the loading control (β-actin). No formal statistical testing was applied to this single replicate.

In (C), n = 12 biologically independent samples (spermatogenic cells isolated from independently treated mice); mRNA levels normalized to those of β-actin and expressed relative to those of the Hypoxia + Vector control; unpaired two-tailed Student’s t test; ****$P < 0.0001$.

Supplementary Figure 4b

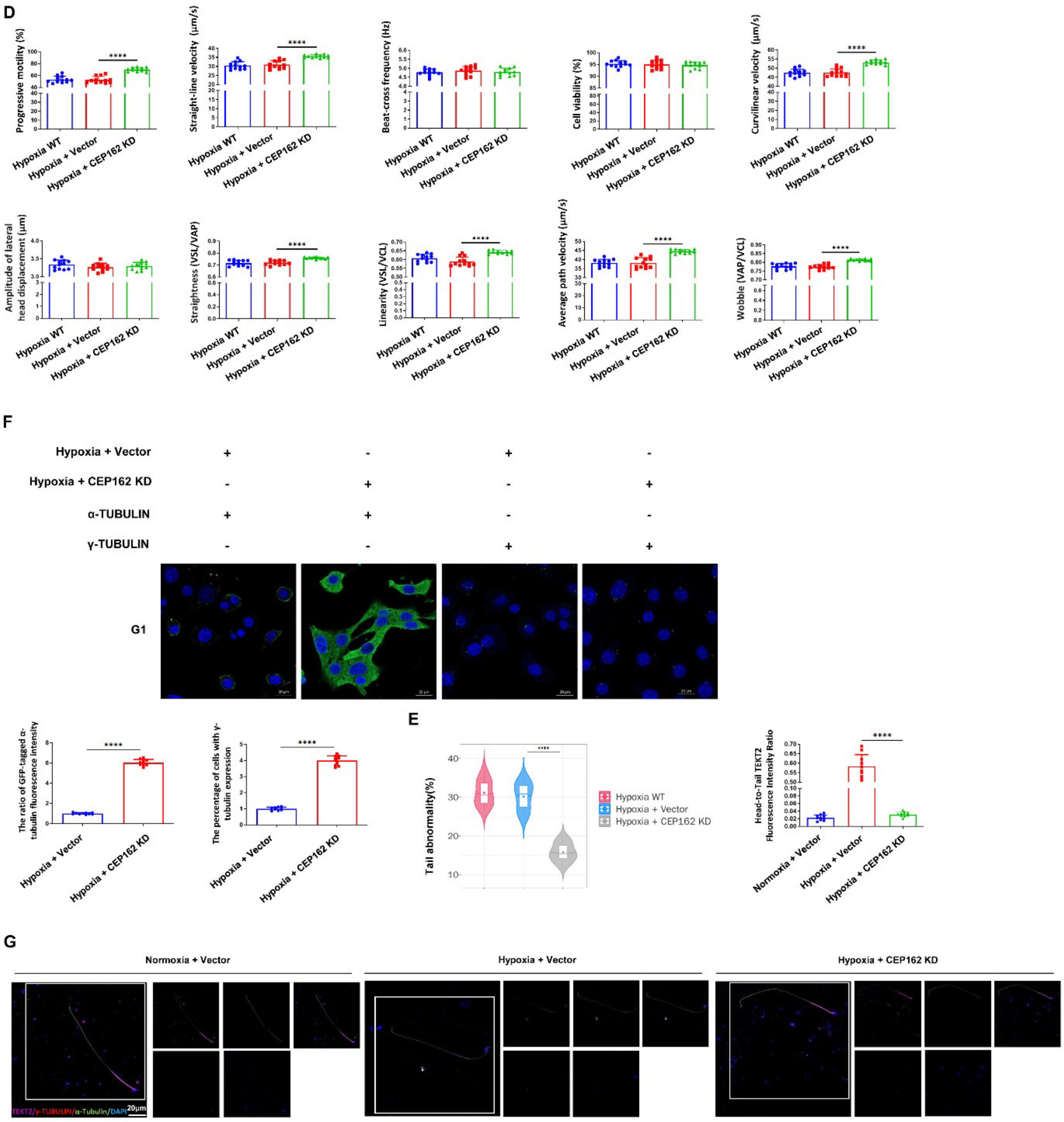

Supplementary Figure 4b(D-G). CEP162 knockdown ameliorates hypoxia-induced defects in sperm motility, restores flagellar morphology, and improves microtubule dynamics and TEKT2 localization. (D) Sperm viability and kinematic parameters in Cep162-knockdown mice under 11.7% O_2 were assessed using computer-assisted sperm analysis (CASA). Motility metrics, including progressive motility, VCL, VSL, VAP, STR, BCF, ALH, LIN, and WOB, were quantified. **(E)** Scanning electron microscopy was used to examine sperm flagellar morphology and quantify tail abnormalities in *Cep162*-knockdown mice exposed to 11.7% oxygen. **(F)** Microtubule regrowth assay in CEP162-knockdown G1 cells after nocodazole washout under 1% O_2. Representative images showing the GFP–α-tubulin fluorescence intensity ratio, indicating the degree of microtubule polymerization. The percentage of cells with focused γ-tubulin foci surrounded by radial α-tubulin asters was quantified as a measure of functional microtubule regrowth. Scale bar = 40

μm. **(G)** Immunofluorescence analysis of TEKT2 localization and its colocalization with γ-tubulin in spermatozoa from CEP162-knockdown mice following hypoxia. Scale bar, 40 μm.

All the data are presented as the mean ± SD.

In (D), n = 12 biologically independent mice; each data point represents one mouse; unpaired t test; ****P < 0.0001.

In (E), n = 10 mice; ≥200 sperm scored per mouse; unpaired t test; ****P < 0.0001.

In (F), n = 10 fields from 3 biological replicates; microtubule regrowth was normalized to the vector mean from the control; unpaired two-tailed t test; ****P < 0.0001.

(G) Representative results of n = 3 independent experiments.

Supplementary Figure 5

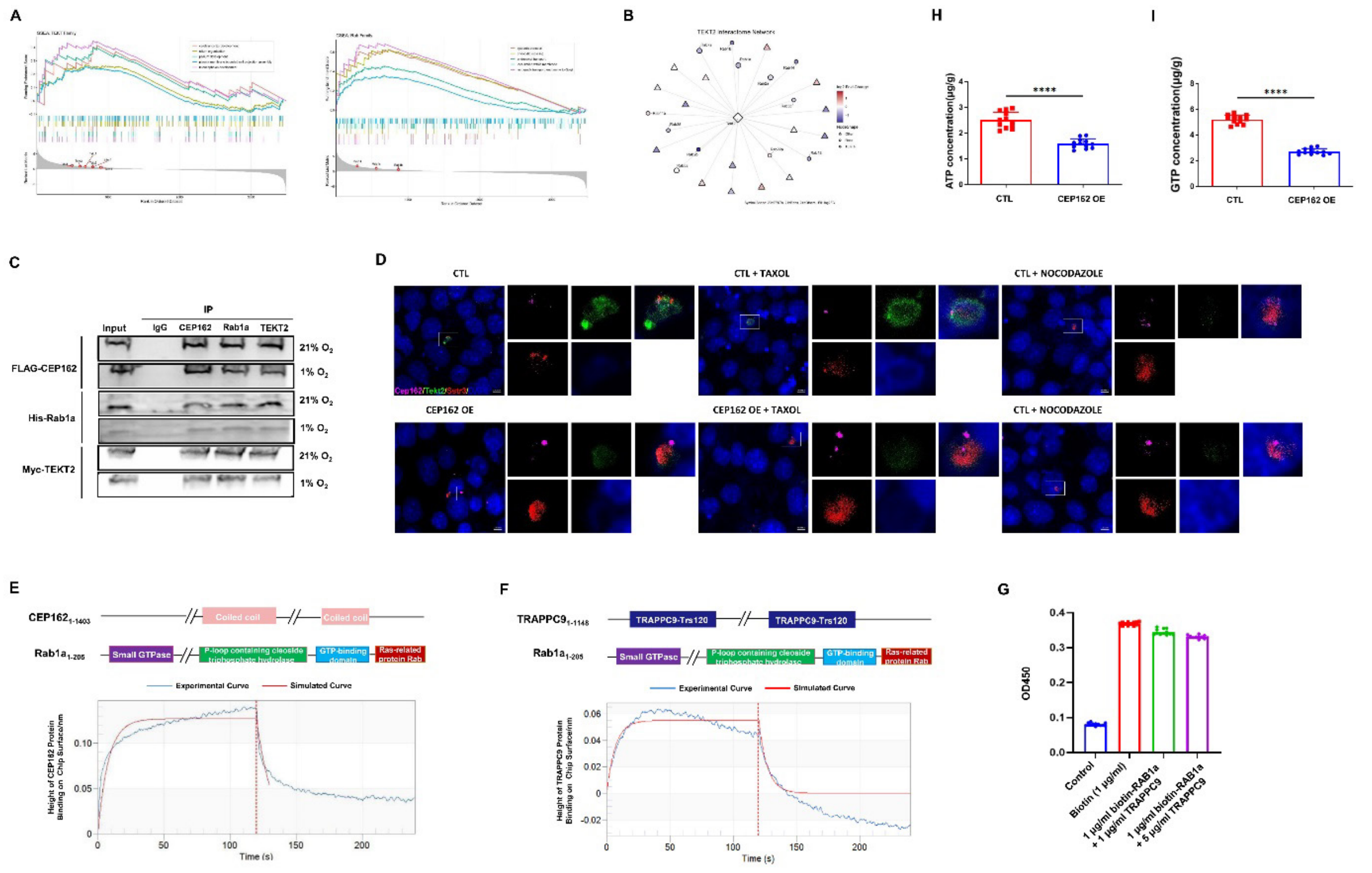

Supplementary Figure 5. Cep162 overexpression disrupts microtubule stability and reduces ciliary ATP and GTP levels. (A) Targeted GSEA of ciliary assembly and endosomal transport pathways. GSEA profiles show the enrichment scores and ranked positions of TEKT family members (Tekt1–5 and Efhc1) and Rab GTPases (Rab1a, Rab1b, and Rab14) within the global proteome. The top panels display the running enrichment score; the bottom panels indicate the rank positions of individual proteins in the ordered list. **(B)** TEKT2-centered protein–protein interaction (PPI) network derived from TEKT2 immunoprecipitates coupled with mass spectrometry. Node shapes denote protein classes: TEKTs (diamonds), Rabs (circles), and other interactors (triangles). Node colors indicate the $\log_2$ fold change values in binding abundance between the CEP162-overexpression group and the control group. **(C)** FLAG-CEP162, His-Rab1a, and Myc-TEKT2 plasmids were individually transfected into G2 cells. Coimmunoprecipitation (Co-IP) assays were performed to examine the interactions among the FLAG-CEP162, His-Rab1a, and Myc-TEKT2 fusion proteins. **(D)** Microtubule regrowth and the localization of TEKT2 in cilia were analyzed in IMCD3 cells overexpressing CEP162 with or without taxol or nocodazole treatment. Immunofluorescence staining was performed using an anti-TEKT2 antibody (green) and an anti-CEP162 antibody (purple), along with DAPI (blue), to visualize microtubules, ciliary proteins, and nuclei, respectively. Scale bars represent 5 μm**.** Representative traces are shown. A biolayer interferometry assay was used to analyze the binding affinity of Rab1a for **(E)** CEP162 and **(F)** TRAPPC9. The representative figure shows sensorgrams of the purified Rab1a protein immobilized on a biosensor chip. **(G)** A competitive ELISA was performed to assess the binding affinity between CEP162

(coated antigen) and biotinylated proteins (biotin-Rab1a), with TRAPPC9 as a potential competitor. **(H)** ATP levels in the cilia of CEP162-overexpressing IMCD3 cells were quantified by liquid chromatography–mass spectrometry. **(I)** Ciliary GTP levels in CEP162-overexpressing IMCD3 cells were measured by liquid chromatography–mass spectrometry.

All the data are presented as the mean ± SD.

In (A–B) n = 3 biological replicates (independently isolated sperm flagella and proteomic runs); volcano plot and GSEA based on label-free quantification (LFQ) intensity values; significance thresholds: $|\log_2(\text{fold change})| \geq 1$ and $P < 0.05$ (two-tailed t test with the Benjamini–Hochberg correction); GSEA FDR q value < 0.05.

(C) Representative image from a single independent experiment. No formal statistical testing was applied to this single replicate.

(D) Representative of n = 3 independent experiments; images used for illustrative purposes only.

(E–F) Representative sensorgrams from biolayer interferometry (BLI) assays analyzing the binding of Rab1a to CEP162 (E) and TRAPPC9 (F). Data are derived from n = 3 biologically independent experiments, with each sample measured in technical triplicates. Owing to the illustrative nature of the kinetic traces, formal statistical testing was not applied to the representative curves.

(G) Representative competitive ELISA curves for assessing the binding affinity between CEP162 and biotinylated Rab1a in the presence of TRAPPC9. The data are derived from 12 biologically independent experiments, with each sample measured in technical triplicates.

In (H–I), n = 12 biological replicates (independent ciliary isolations); unpaired two-tailed t test; $^{****}P < 0.0001$.

Supplementary Figure 6

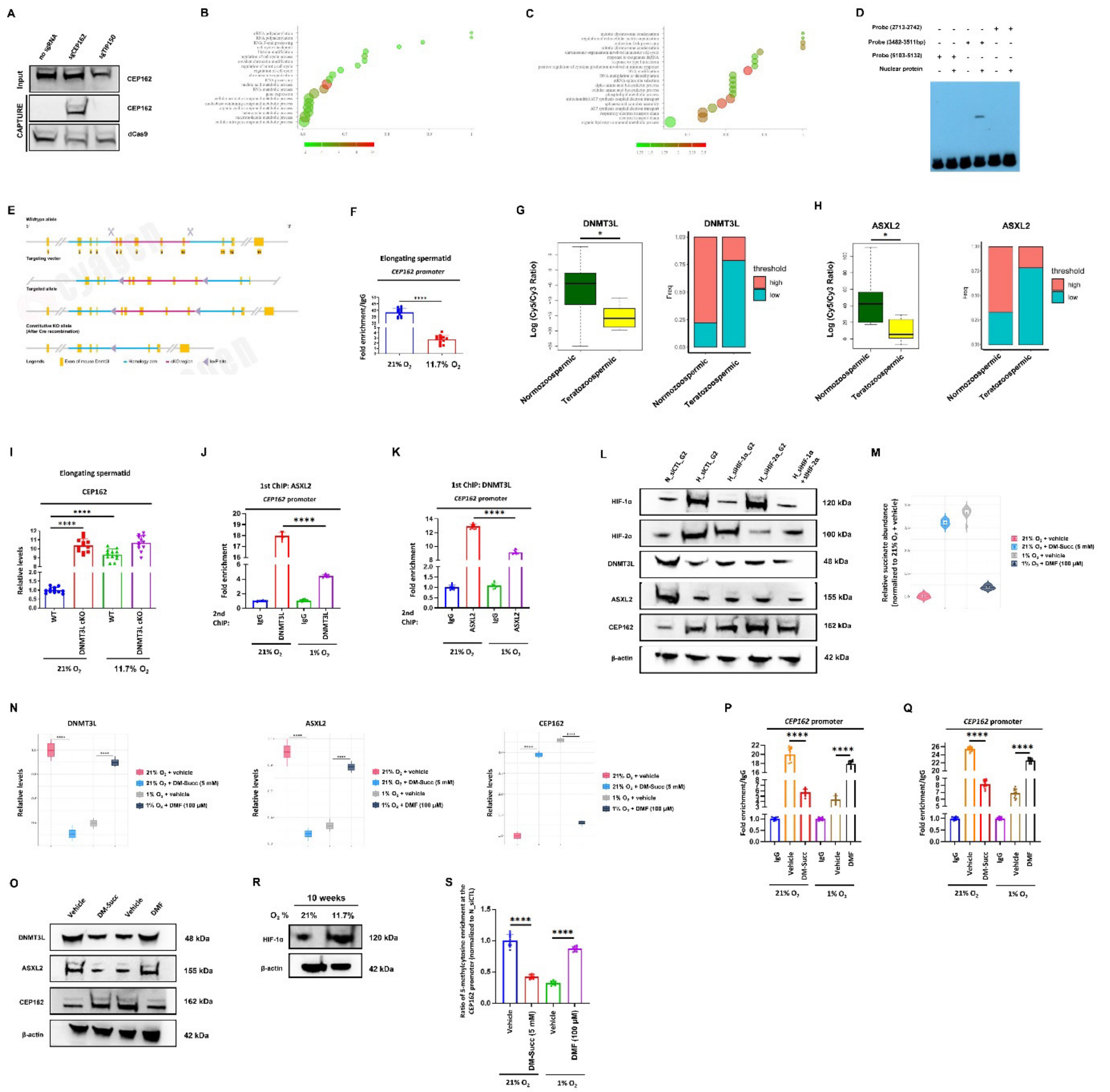

Supplementary Figure 6. Characterization of the role of DNMT3L in regulating CEP162 under hypoxia and validation of the DNMT3L conditional knockout model. (A) Western blot analysis of CEP162 enrichment under dCas9 and/or sgCEP162 conditions. **(B)** GO analysis of shared differentially expressed genes ($|\log_2 FC| \geq 1$) in N_RS and H_RS. **(C)** GO enrichment of common differentially expressed genes ($|\log_2 FC| \geq 1$) in N_ES and H_ES. **(D)** EMSAs were used to assess the binding of nuclear proteins to the DNMT3L-binding probe within the CEP162 promoter, spanning +222 to +251 bp relative to the TSS, along with two negative control probes spanning −392 to −363 bp and −2373 to −2338 bp. **(E)** Schematic diagram depicting the targeting strategy used to generate spermatogenic cell-specific conditional *Dnmt3l*-knockout (cKO) mice. Exons 6 to 10 of the mouse DNMT3L gene are flanked by loxP sites in the conditional knockout allele. Cre recombinase-mediated excision of the floxed region results in a frameshift and a null allele, leading to loss of Dnmt3l function. **(F)** ChIP–qPCR analysis of DNMT3L binding to the CEP162 promoter in ES from

rats exposed to 21% or 11.7% oxygen for 10 weeks. Normalized log_2(Cy5/Cy3) expression of DNMT3L **(G)** and ASXL2 **(H)** in normozoospermic controls (n = 9) and individuals with teratozoospermia (n = 14) retrieved from the GEO dataset GSE6969. Teratozoospermic patients were subdivided into “high” or “low” expression groups for each gene relative to the respective mean transcript level within the case cohort. **(I)** qPCR analysis of CEP162 mRNA expression levels in ES from WT and DNMT3L cKO mice exposed to 21% or 11.7% oxygen for 10 weeks (n = 12). Sequential ChIP–qPCR analysis of **(J)** DNMT3L and **(K)** ASXL2 cooccupancy at the CEP162 promoter in G2 cells under normoxic and hypoxic conditions (n = 12). The first and second ChIP antibodies are indicated in the panel titles and x-axis labels, respectively. **(L)** Western blot analysis of HIF-1α, HIF-2α, DNMT3L, ASXL2, and CEP162 protein expression in G2 cells transfected with control siRNA (N_siCTL_G2 or H_siCTL_G2) or siRNAs targeting HIF-1α (H_siHIF-1α_G2), HIF-2α (H_siHIF-2α_G2), or both HIF-1α and HIF-2α (H_siHIF-1α + siHIF-2α_G2). **(M)** Intracellular succinate accumulation in G2 cells under metabolic perturbation. Cells were treated with 21% O_2 + vehicle, 21% O_2 + DM-Succ (5 mM dimethyl succinate), 1% O_2 + vehicle, or 1% O_2 + DMF (100 μM dimethyl fumarate). **(N)** qPCR analysis of the relative mRNA expression levels of DNMT3L, ASXL2, and CEP162 in G2 cells under the same treatment conditions as in **(N)**. **(O)** Western blot analysis of DNMT3L, ASXL2, and CEP162 protein expression in G2 cells treated with 21% O_2 + vehicle, 21% O_2 + DM-Succ (5 mM), 1% O_2 + vehicle, or 1% O_2 + DMF (100 μM). **(P)** ChIP–qPCR analysis of DNMT3L binding to the CEP162 promoter in G2 cells under the following conditions: 21% O_2 + vehicle, 21% O_2 + DM-Succ (5 mM), 1% O_2 + vehicle, and 1% O_2 + DMF (100 μM). **(Q)** ChIP–qPCR analysis of ASXL2 binding to the CEP162 promoter in G2 cells under the following conditions: 21% O_2 + vehicle, 21% O_2 + DM-Succ (5 mM), 1% O_2 + vehicle, and 1% O_2 + DMF (100 μM). **(R)** Western blot analysis showing HIF-1α protein expression in spermatogenic cells from mice exposed to 21% O_2 or 11.7% O_2 for 10 weeks. **(S)** Methylated DNA immunoprecipitation followed by qPCR (MeDIP–qPCR) analysis of 5-methylcytosine (5-mC) enrichment at the CEP162 promoter in G2 cells under the indicated metabolic interventions.

All the data are presented as the mean ± SD.

(A, D) Representative images from a single independent experiment. No formal statistical testing was applied to this single replicate.

(B–C) Gene Ontology (GO) enrichment analysis of shared differentially expressed genes. Statistical significance was determined using the hypergeometric test, and P values were adjusted for multiple comparisons using the Benjamini–Hochberg false discovery rate (FDR) method. Only GO terms with an FDR-adjusted $P < 0.05$ were considered significantly enriched.

(E) Schematic diagram depicting the targeting strategy; no statistical testing was applied.

In (F, I, J–K), n = 12 biological replicates (spermatids or independent G2 cell cultures); qPCR/ChIP signals were normalized to that or β-actin (mRNA) or input DNA (ChIP); two-way ANOVA with Tukey’s post hoc test or one-way ANOVA with Tukey’s post hoc test (K); ****$P < 0.0001$.

In (G-H), n = 9 (controls) and n = 14 (patients) from GSE6969; data were subjected to log_2 transformation (Cy5/Cy3); unpaired two-tailed Student’s t test; *$P < 0.05$.

(L) Representative image from a single independent experiment; protein signals were normalized to that of the loading control (β-actin). No formal statistical testing was applied to this single replicate.

In (M–N), n = 12 biological replicates (independent G2 cell cultures); intracellular succinate (M) and qPCR signals (N) were normalized to those in the vehicle control under 21% O_2; protein levels (O) were normalized to that of β-actin; one-way ANOVA with Tukey's post hoc test; $^{****}P < 0.0001$.

(O) Representative image from a single independent experiment; protein signals were normalized to that of the loading control (β-actin). No formal statistical testing was applied to this single replicate.

In (P–Q), n = 12 biological replicates (independent G2 cell cultures); ChIP signals were normalized to input DNA; one-way ANOVA with Tukey's post hoc test; $^{****}P < 0.0001$.

(R) Representative image from a single independent experiment; protein signals were normalized to that of the loading control (β-actin). No formal statistical testing was applied to this single replicate.

In (S), n = 12 biological replicates per group, normalized to the vehicle control under 21% O_2 (set as 1.0); one-way ANOVA with Tukey's post hoc test; $^{****}P < 0.0001$.

Supplementary Figure 7

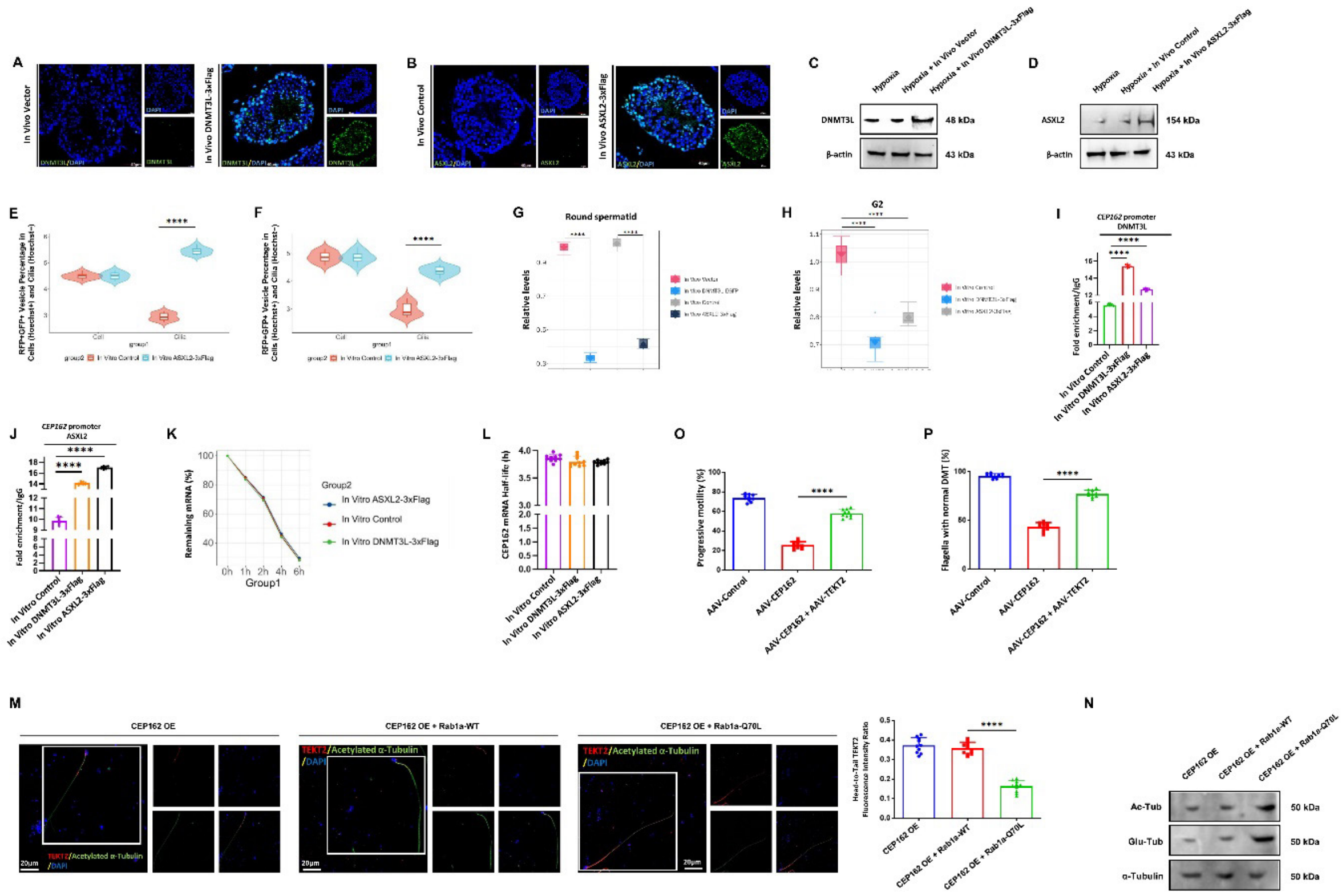

Supplementary Figure 7. ASXL2 activates CEP162 transcription to restore vesicular trafficking under hypoxia. (A) Immunofluorescence analysis of DNMT3L protein expression in the seminiferous tubules of mouse testes from the In Vivo Vector and In Vivo DNMT3L-3×Flag groups. **(B)** Immunofluorescence analysis of ASXL2 protein expression in the seminiferous tubules from the testes of mice in the In Vivo Control and In Vivo ASXL2-3×Flag groups. **(C)** Western blot analysis of DNMT3L protein levels in spermatogenic cells isolated from the testes of mice in the In Vivo Vector and In Vivo DNMT3L-3×Flag groups. The 3×FLAG epitope adds ~3.5 kDa; the fusion protein migrates at ~48 kDa on a standard 10% SDS–PAGE gel. **(D)** Western blot analysis of ASXL2 protein levels in spermatogenic cells isolated from the testes of mice in the In Vivo Control and In Vivo ASXL2-3×Flag groups. **(E)** Flow cytometry was used to assess the colocalization of Clathrin and SSTR3 in IMCD3 cells under hypoxia. The proportions of vesicle-positive cells (RFP^+GFP^+ among $Hoechst^+$ cells) and cilia-targeted vesicles (RFP^+GFP^+ among $Hoechst^-$ cells) were quantified in the In Vitro Control and In Vitro ASXL2-3×Flag groups. Clathrin (GFP) was used to label clathrin-coated vesicle fusion pores, and SSTR3 (RFP) was used to label the primary cilium. **(F)** Rab1a (GFP) and SSTR3 (RFP) double-positive populations were analyzed by flow cytometry to evaluate ciliary vesicle trafficking. Compared with the control, the overexpression of ASXL2-3×Flag altered the distribution of recycling vesicles to cilia, indicating a role in hypoxia-regulated

ciliary transport. **(G)** CEP162 mRNA levels in round spermatids under hypoxia were assessed in the mice in the In Vivo Control, In Vivo Vector, In Vivo DNMT3L-EGFP, and In Vivo ASXL2-3×Flag groups by quantitative RT–PCR. **(H)** Quantitative RT–PCR analysis of CEP162 mRNA expression in G2 cells overexpressing DNMT3L-3×Flag or ASXL2-3×Flag under hypoxic conditions relative to that in the In Vitro Control group. ChIP–qPCR analysis of **(I)** DNMT3L and **(J)** ASXL2 binding to the CEP162 promoter in G2 cells under hypoxia in the In Vitro Control, In Vitro DNMT3L-3×Flag, and In Vitro ASXL2-3×Flag groups. **(K)** Kinetics of CEP162 mRNA decay following transcriptional inhibition. G2 cells from the In Vitro Control, In Vitro DNMT3L-3×Flag, and In Vitro ASXL2-3×Flag groups were treated with actinomycin D (5 μg/mL) to block new RNA synthesis. mRNA levels of mature CEP162 were quantified by qPCR using exon-specific primers at the indicated time points. **(L)** Quantification of the CEP162 mRNA half-life across experimental groups. **(M)** Immunofluorescence analysis of endogenous TEKT2 localization in sperm flagella from the indicated groups (CEP162 OE, CEP162 OE + Rab1a-WT, and CEP162 OE + Rab1a-Q70L). Scale bar, 20 μm. Quantification of the head-to-tail TEKT2 fluorescence intensity ratio across the indicated groups. **(N)** Western blot analysis of the expression of polymerized microtubule markers, including acetylated α-tubulin (Ac-Tub) and detyrosinated α-tubulin (Glu-Tub), normalized to total α-tubulin expression. **(O)** Computer-assisted sperm analysis (CASA) quantification of progressive motility in the indicated groups. **(P)** Quantification of the percentage of flagellar cross-sections displaying normal axonemal doublet microtubules (DMTs).

All the data are presented as the mean ± SD.

(A–B) Representative images from 5 biologically independent mice per group. Each sample was assessed in 3 independent immunofluorescence experiments, with a total of ≥50 fields visualized across all samples; no formal statistical analyses performed for these qualitative imaging assays.

(C–D) Representative image from a single independent experiment; protein signals were normalized to that of the loading control (β-actin); no formal statistical testing was applied to this single replicate.

In (E–F, G–J), n = 12 (E, F, G, H) or n = 10 (I, J) biological replicates (independent cell cultures or mouse samples); qPCR/ChIP signals normalized to that of β-actin (mRNA) or input DNA (ChIP); two-way ANOVA with Tukey’s post hoc test; ****$P < 0.0001$.

In (K–L), n = 10 biological replicates; mRNA decay curves were fitted to a one-phase exponential decay model; the half-life calculated from the slope; two-way ANOVA with Tukey’s post hoc test.

In (M), n = 12 biologically independent mice; ≥100 sperm were scored per mouse for immunofluorescence analysis; the head-to-tail TEKT2 fluorescence intensity ratio was quantified using ImageJ; one-way ANOVA with Tukey's post hoc test; ****$P < 0.0001$.

In (O), n = 12 biologically independent mice; each data point represents one mouse; one-way ANOVA with Tukey's post hoc test; ****$P < 0.0001$.

In (P), n = 10 biologically independent mice; ≥200 flagellar cross-sections were scored per group by a blinded observer; one-way ANOVA with Tukey's post hoc test; ****$P < 0.0001$.
